# Seasonal dynamics of leaf macro- and micronutrients in three commercial avocado varieties at Mojo and Melkassa, Ethiopia

**DOI:** 10.1371/journal.pone.0362564

**Published:** 2026-11-12

**Authors:** Zeyede Aregahegn, Edossa Etissa, Israel Bekele, Dejene Abera

**Affiliations:** 1 Ethiopian Institute of Agricultural Research, Melkassa Agricultural Research Center, Adama, Ethiopia; 2 Department of Chemical Sciences, Ariel University, Ariel, Israel; University of Duhok, IRAQ

## Abstract

Seasonal leaf nutrient data needed to guide fertilization are scarce for Ethiopian avocado orchards. We measured total nitrogen (TN), Ca, Cu, Fe, Mn and Zn in leaves of tagged Hass, Fuerte and Ettinger trees at the Melkassa Agricultural Research Center (February–July) and of Hass trees on three farms at Mojo (February–June), and described the soils of both sites. Data were analysed with linear mixed models, Tukey’s test and repeated-measures correlation. Mean leaf TN (1.13–1.41%) was below the sufficiency range in all varieties and did not differ significantly between months, varieties or sites, and leaf Zn was below 20 mg/kg in most samples. Leaf Ca increased from 0.79–1.00% in February to 1.61–1.65% in July in all varieties at Melkassa. Cu, Fe, Mn and Zn followed variety-specific seasonal patterns, and Hass leaves at Mojo contained more Mn, Cu and Zn than those at Melkassa. Within trees, Mn was positively correlated with Ca in all varieties and with Cu and Zn in Hass and Fuerte. Soils at both sites were neutral to alkaline (pH 7.24–8.50), very low to low in TN (0.05–0.18%) and mostly very low to low in DTPA-extractable Zn and Fe, in line with the low leaf N and Zn. Nitrogen and zinc supply may limit both orchards, leaf Fe should be monitored on these alkaline soils, and leaf samples for diagnosis should be taken at a fixed time of year because Ca and micronutrients change strongly with season.

## Introduction

Avocado is well adapted to several agroecological zones of Ethiopia and is grown for both domestic and export markets [1,2]. Avocado cultivation can raise farmers’ incomes, make better use of land and support food security in growing regions. The fruit is rich in essential nutrients and is recommended for combating malnutrition in Ethiopia [1]. The avocado tree can grow to 10–12 m and, in its natural subtropical to tropical climates, can bear fruit for much of the year; it is highly productive [1].

Hass, Fuerte and Ettinger are the main commercial avocado cultivars in Ethiopia. They are valued for their adaptability, yield, fruit quality and contribution to smallholder incomes and the country’s growing avocado trade [1,2]. Hass has a long fruiting season, Fuerte supports consistent production when planted with Hass, and Ettinger forms vigorous, early-maturing trees with good fruit quality [3].

Macronutrient (K, Ca, Mg, N, P, S) and micronutrient (Fe, Mn, Cu, Zn, B, Mo) concentrations in fruit-tree leaves vary across phenological stages (flowering, fruit development, fruit maturity and post-harvest) [4,5]. Leaf analysis is therefore used to judge whether trees receive enough of each nutrient at a given time of year [6,7]. Successful avocado establishment also depends on soil conditions, such as adequate organic matter and a suitable pH, and on appropriate fertilization, irrigation and mulching [8].

Leaf nutrient status is closely linked to photosynthesis and therefore to the tree’s demand for nutrients [7,9]. Nitrogen is needed for amino acids, proteins and chlorophyll [10]; magnesium is part of chlorophyll; calcium helps the tree resist *Phytophthora* and fruit rots; sulfur is needed for protein synthesis; and boron is important for fruit set [11–13]. Micronutrients such as Fe, Mn, Zn, Cu and Mo are required for normal growth and enzyme function [14,15]. Analysis of macro- and micronutrients in avocado leaves supports better fertilizer timing and rates, sustainable nutrient management, fruit quality, disease and stress management and, ultimately, profitability [7,16].

The avocado tree needs a balanced supply of nutrients for high yield, and deficiencies of nitrogen, potassium and zinc reduce fruit yield [7,9]. In Ethiopia, studies on avocado and soil have mainly examined how avocado trees in smallholder agroforestry systems change soil properties [17,18], whereas information on the nutrient status of the avocado trees themselves is limited. Knowing how leaf nutrient concentrations change during the season is needed to set an optimal fertilization schedule, but such seasonal data are not available for avocado orchards in central Ethiopia. The objectives of this study were therefore (i) to quantify seasonal changes in leaf total nitrogen (TN), Ca, Cu, Fe, Mn and Zn in Hass, Fuerte and Ettinger at the Melkassa Agricultural Research Center (MARC) and in Hass at Mojo, (ii) to examine the relationships among these leaf nutrients, and (iii) to describe the soil properties of the orchards at both sites as a basis for interpreting the leaf results. We hypothesized, first, that leaf nutrient concentrations change during the season and that this seasonal pattern differs between varieties, and second, that the soil N, Fe and Zn status is reflected in the leaf concentrations of these nutrients.

## Materials and Methods

### Study sites

The study was carried out at two sites in East Shewa Zone, Oromia Region, Ethiopia (Fig 1). At Mojo, sampling was restricted to Hass trees on three commercial farms in Lume Woreda (8°39′N, 39°5′E; 1,788–1,825 m above sea level). The Mojo catchment receives a mean annual rainfall of about 947 mm and has a mean annual air temperature of about 20.4 °C [19]. The Hass trees at Mojo were planted in 2021. Leaf sampling at Mojo started about one year after planting, at the flush stage in February, and continued until June; it was stopped when the fruit reached maturity, about 18 months after planting. The Mojo orchards are managed by the farm owners with practices similar to those at MARC. At MARC in Adama Woreda (8°24′N, 39°21′E; 1,550 m above sea level; 17 km from Adama and about 107 km from Addis Ababa), Hass, Fuerte and Ettinger trees were sampled in the avocado experimental orchard, which was planted in 2001 at 6 m × 6 m spacing [20]. MARC lies in the semi-arid Central Rift Valley, with a mean annual rainfall of about 768 mm, mean maximum temperatures of 26.4–30.1 °C and mean minimum temperatures of 10.4–16.4 °C [21]. At both sites, more than 80% of the annual rainfall falls in the main rainy season (*kiremt*, June to September), and a short rainy season (*belg*) occurs from February to May [19,22]. The MARC trees are furrow-irrigated, and all MARC trees receive the same agronomic management. The map of the study area (Fig 1) was prepared in ArcGIS version 10.7.1 (Esri, Redlands, CA, USA) by placing the two sites on the 2023 administrative boundary map of Ethiopia (national, regional, zonal and woreda boundaries); all layers were projected to UTM zone 37N.

**Fig 1:**
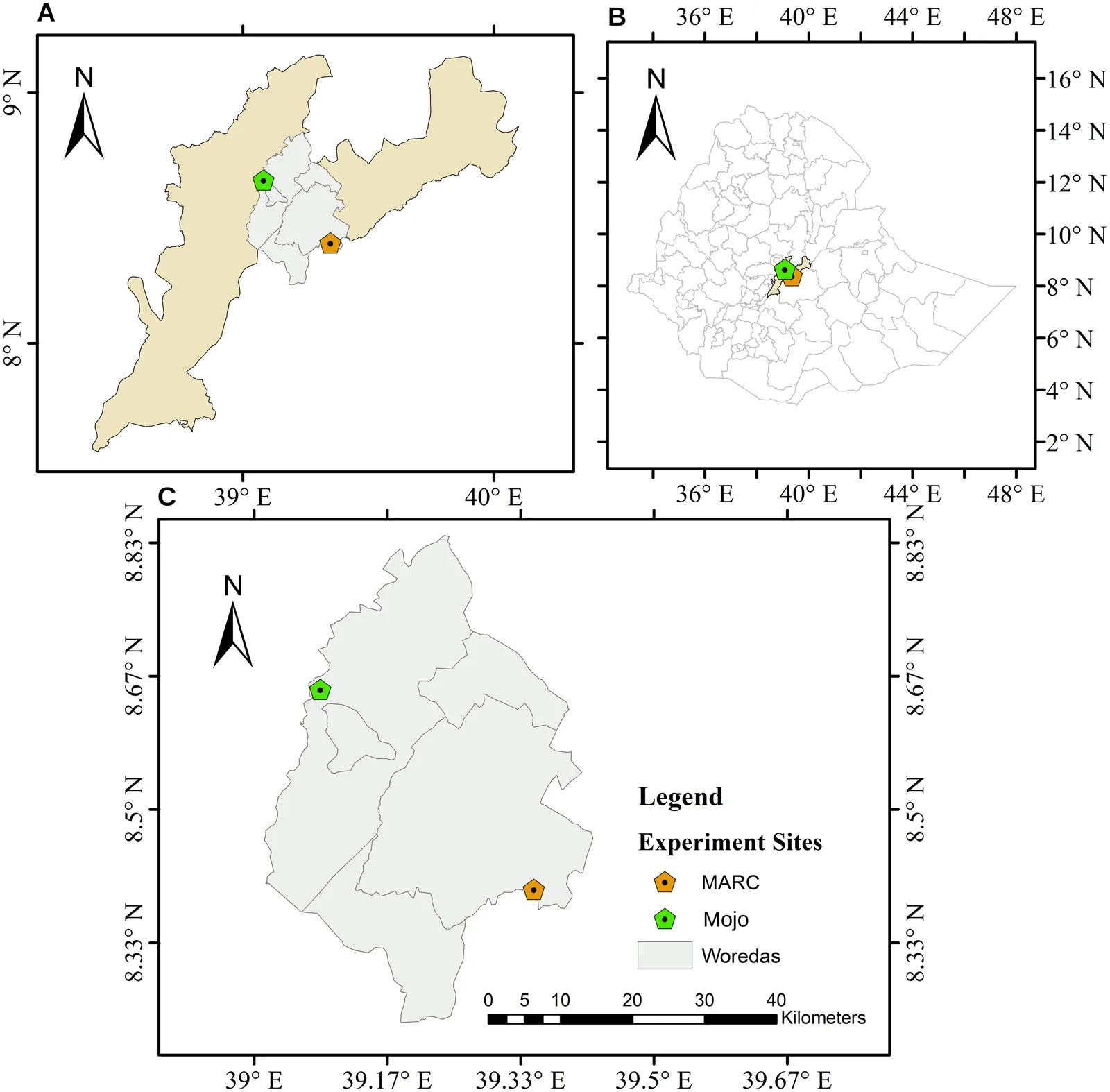
Map of the study area. (A) East Shewa Zone (beige) with the woredas of the study area (grey); (B) location of the study area in Ethiopia; (C) the study area with the two study sites: the commercial Hass farms at Mojo (Lume Woreda, green) and the avocado orchard of the Melkassa Agricultural Research Center (MARC, Adama Woreda, orange). The map was created by the authors in ArcGIS 10.7.1 using the 2023 administrative boundaries of Ethiopia.

Sampling at MARC was carried out in the Center’s own experimental orchard with the approval of the Melkassa Agricultural Research Center, Ethiopian Institute of Agricultural Research (EIAR). Leaf and soil samples on the privately owned farms at Mojo were collected with the oral consent of the farm owners, who volunteered to take part in the study and are familiar with the research activities of MARC and EIAR. No other permits were required because the work was done on cultivated land and did not involve protected areas or protected or endangered species. The study did not involve human participants or animals; therefore, approval by an ethics committee was not required.

### Sampling of avocado leaves

Sampling trees were selected in mid-January by horticulture and soil fertility researchers of MARC. At Mojo, three Hass trees were randomly selected and tagged on each of three farms (nine trees in total), taking into account the flowering stage of the trees and their position in the farm landscape. Only Hass could be sampled at Mojo because the farmers grow only Hass, which they prefer for both export and local markets; the three varieties were therefore compared at MARC, where nine trees of each variety (Hass, Fuerte and Ettinger; 27 trees in total) were randomly selected and tagged. Thus, 9 trees at Mojo and 27 trees at MARC were sampled in each sampling month. Sampling started at the flush stage in February and the same tagged trees were sampled in February, March, May, June and July of the 2022 production season; no samples were collected in April. Mojo was not sampled in July because the fruit had reached maturity. On each occasion, healthy, fully expanded leaves were taken from the same tagged, non-shaded branches in the middle of the canopy [23], so the sampled leaves aged as the season progressed. On each occasion, about 16 leaves (four from each of four canopy directions) were collected from each tree and combined into one composite sample, which was analysed in three replicates; the mean of the replicates was used as the value for that tree. The tree is therefore the experimental unit of this study. Samples were collected following standard procedures [6,24] and transported to the MARC Soil, Plant and Water Analysis Laboratory for the analysis of TN, Ca, Cu, Fe, Mn and Zn. A few samples were not available (one Hass tree at MARC in February, another in March, and two Fuerte trees at MARC in July), and Mn and Zn were not determined for one further Fuerte tree in July. The data set therefore contains 36 observations for Hass at Mojo and 43, 43 (42 for Mn and Zn) and 45 observations for Hass, Fuerte and Ettinger at MARC (S1 Dataset).

### Leaf sample preparation and analysis

After collection, the leaves were washed with distilled water, air-dried, ground with an electric grinder and sieved through a 0.5 mm sieve. A 0.5 g portion of the ground, homogenized sample was weighed into an acid-washed porcelain crucible and dry-ashed in a muffle furnace at 500 °C for 5 h, following the procedures described by Estefan et al. [25] and Wogi et al. [24]. The ash was dissolved in 2 N HCl and made up to 100 mL for the determination of Ca, Cu, Fe, Mn and Zn. Leaf TN was determined by the Kjeldahl method after digestion with concentrated H_2_SO_4_ and a catalyst mixture [25,26].

### Soil sampling and analysis

Soil data were used to describe the soils of the orchards at both sites. At Mojo, samples were collected in December 2021, before leaf sampling started, from the Hass orchards of the three farms used for leaf sampling: one point on farms 1 and 2 and two points (P1 and P2) on farm 3, each at 0–20 and 20–50 cm depth. No pre-planting soil data were available for these farms. At MARC, samples were collected in 2021 at three points (P1–P3) in the avocado orchard, at the same depths (0–20 and 20–50 cm). The samples of both sites were analysed in the same laboratory with the same methods [24,25,27]: the samples were air-dried and sieved, pH and electrical conductivity (EC) were measured in a 1:2.5 soil:water suspension, organic carbon (OC) by the Walkley–Black method [28], total nitrogen (TN) by the Kjeldahl method [26], cation exchange capacity (CEC) by the 1 M ammonium acetate (pH 7) method [29], particle-size distribution by the Bouyoucos hydrometer method [30], and Fe, Cu, Zn and Mn after extraction with diethylenetriaminepentaacetic acid (DTPA) at a soil:solution ratio of 1:2 [31].

### Apparatus and equipment

A benchtop pH meter and an electrical conductivity meter were used to measure soil pH and EC, respectively. A flame atomic absorption spectrophotometer (FAAS; Agilent 200 Series AA, USA) was used for multi-element analysis of Cu, Fe, Mn and Zn in soil extracts and of Ca, Cu, Fe, Mn and Zn in leaf digests. A Kjeldahl digestion and distillation unit and a digital burette were used to determine total Kjeldahl nitrogen in soil and leaf samples. A programmable furnace (Nabertherm, Germany) was used to ash the leaf samples.

### Reagents and standards

All reagents and chemicals were analytical-grade. Certified 500 mg/L stock solutions (Agilent Technologies, USA) of Cu, Mn, Fe and Zn were used to prepare the standard series. 2 N HCl (Aldrich, A.C.S. Reagent, Germany) was used to dissolve the ash of the leaf samples. Deionized water and DTPA reagent [a mixture of 0.005 M DTPA, 0.1 M triethanolamine (TEA) and 0.01 M CaCl_2_, pH 7.3] were used to prepare and dilute the micronutrient standards for soil analysis, and 5% HNO_3_ was used to prepare the series of standards for leaf analysis. A lanthanum solution was added to the Ca standards and leaf sample solutions as a releasing agent to suppress phosphate interference.

### Calibration

An intermediate standard solution of 50 mg/L was prepared for each metal by diluting 10 mL of the 500 mg/L Agilent certified reference stock solution to 100 mL with 5% HNO_3_. From this intermediate solution, working standards (including a blank) were prepared to construct calibration curves for each element in each analytical batch: 0, 0.4, 0.8, 1.2, 1.6 and 2.0 mg/L of Fe, Cu, Mn and Zn for soil extracts, 0, 2, 4, 6, 8 and 10 mg/L of Fe, Cu, Mn and Zn for leaf digests, and 0, 2, 5, 7 and 9 mg/L of Ca for leaf digests.

### Quality assurance and quality control

The instrumental conditions of the FAAS for each element are shown in Table 1. Concentrations in solution are expressed in mg/L and concentrations in soil and leaf dry matter in mg/kg (TN and Ca in leaves, and OC and TN in soil, in %).

**Table 1:** Instrumental conditions of the flame atomic absorption spectrophotometer (Agilent 200 Series AA) used for each element.

Metals	Wavelength	Lamp current	Slit width	Optimum working range
	nm	mA	nm	mg/L
Mn	279.5	5	0.2	0.02–50
Cu	324.7	4	0.5	0.03–10
Zn	213.9	5	1.0	0.01–20
Fe	248.3	5	0.1	0.06–15
Ca	422.7	10	0.5	0.01–3.00

### Recovery analysis

Matrix effects were evaluated by spiking soil and leaf extracts with standard solutions of the analytes and calculating the recovery (Eq 1):


$$\begin{array}{c} \%Recovery=\;\frac{C_{s}-\;C_{unsp}}{C_{added}}\;\times \text{1}\text{0}\text{0} \end{array}$$ (1)

where C_s_ is the concentration of the spiked sample, C_unsp_ is the concentration of the unspiked sample and C_added_ is the added concentration.

For Cu, Mn, Fe and Zn, 20 mL of soil or leaf extract was spiked with 2 mL of an 8 mg/L standard solution. For Ca, 20 mL of leaf extract was spiked with 2 mL of a 50 mg/L Ca standard solution.

### Limit of detection (LOD) and limit of quantification (LOQ)


$$\begin{array}{c} LOD=\frac{\text{3}\times \delta }{S} \end{array}$$ (2)

$$\begin{array}{c} LOQ=\frac{\text{1}\text{0}\times \delta }{S} \end{array}$$ (3)

where LOD is the limit of detection, LOQ is the limit of quantification, δ is the standard deviation of the blank measurements and S is the slope of the calibration curve. The linearity of the calibration curves, the limits of detection and quantification and the spike recoveries are given in Tables 2 and 3.

**Table 2:** Calibration and quality-control data for the soil analysis.

Metals	CIS	CWS	CCCC	LOD	LOQ	Recovery
	mg/L					%
Mn	50	0, 0.4, 0.8, 1.2, 1.6, 2.0	0.9996	0.055	0.168	96.48
Cu	50	0, 0.4, 0.8, 1.2, 1.6, 2.0	0.9999	0.026	0.078	96.93
Zn	50	0, 0.4, 0.8, 1.2, 1.6, 2.0	0.9999	0.027	0.081	95.90
Fe	50	0, 0.4, 0.8, 1.2, 1.6, 2.0	0.9998	0.040	0.122	----------

CIS = concentration of intermediate standard, CWS = concentration of working standards, CCCC = correlation coefficient of calibration curve, LOD = limit of detection, LOQ = limit of quantification. CIS and CWS are in mg/L; LOD and LOQ are expressed on a sample basis (mg/kg dry weight). Recovery of Fe in soil extracts was not determined.

**Table 3:** Calibration and quality-control data for the leaf analysis (abbreviations as in Table 2).

Metals	CIS	CWS	CCCC	LOD	LOQ	Recovery
	mg/L					%
Mn	50	0, 2, 4, 6, 8, 10	0.9999	0.132	0.399	104.07
Cu	50	0, 2, 4, 6, 8, 10	0.9999	0.002	0.005	95.80
Zn	50	0, 2, 4, 6, 8, 10	0.9999	0.220	0.667	96.19
Fe	50	0, 2, 4, 6, 8, 10	0.9997	0.221	0.668	102.42
Ca	50	0, 2, 5, 7, 9	0.9998	0.210	0.636	95.49

### Statistical analysis

The tagged tree was the experimental unit. For each tree and sampling month, the mean of the replicate analyses of the composite leaf sample was used, and all statistics were calculated from these tree-level values, not from monthly means. Values reported by the laboratory as below the detection limit (ND) occurred mainly for Cu in February and March (47 of 167 Cu values, 10 of 167 Fe values and 5 of 166 Zn values; S1 Dataset). For the statistical analysis, ND values were set to half of the lowest concentration quantified for that element (0.20 mg/kg for Cu, 0.50 mg/kg for Fe and 0.44 mg/kg for Zn), a common substitution for values below the reporting limit [32]. To check that the results did not depend on this choice, the analyses of Cu, Fe and Zn were repeated with ND values set to a very low value (zero for Cu and one-tenth of the lowest quantified concentration for the log-transformed Fe and Zn) and to the lowest quantified concentration; all conclusions were the same, except that the month effect on Fe at Mojo, which was not significant in the main analysis, varied around the 0.05 level (P = 0.045–0.069; S1 Table).

Seasonal and varietal differences at MARC were analysed with a linear mixed model with variety, sampling month and their interaction as fixed effects and tree as a random effect. The random tree effect accounts for the repeated sampling of the same trees. Seasonal differences in Hass at Mojo were analysed with sampling month as a fixed effect and farm and tree (nested within farm) as random effects. Because only three farms were sampled, the farm variance was small and was estimated as zero for Fe, in which case the model was fitted with tree only; treating farm as a fixed effect gave the same P values for month (S1 Table). Hass trees at the two sites were compared for the four months sampled at both sites (February, March, May and June), with site, month and their interaction as fixed effects and tree as a random effect. Models were fitted by restricted maximum likelihood with the lme4 package [33], and fixed effects were tested with type III F-tests using Kenward–Roger degrees of freedom [34] in the lmerTest package [35]. The full F-test results are given in S1 Table.

Model assumptions were checked with normal Q–Q plots and the Shapiro–Wilk test of the residuals and with Levene’s test for homogeneity of variance. TN, Fe and Zn were right-skewed and were analysed after natural-log transformation, and Ca, Cu and Mn on the original scale; the same scale was used for each nutrient in all analyses so that the results are comparable (residual checks in S1 Table). Because a few very high Fe and Zn values remained after transformation, the F-tests for Fe and Zn were repeated with the aligned rank transform [36], which does not assume normally distributed residuals; every effect that was significant in the mixed models was also significant in this analysis (S1 Table). In months in which Cu was below the detection limit in all trees of a group (Mojo in February, Hass at MARC in March and Fuerte at MARC in February and March), all substituted values were equal, and the Cu tests mainly show that Cu was undetectable in these months. Means were separated with Tukey’s honestly significant difference (HSD) test on estimated marginal means (emmeans package [37]) at P < 0.05. At MARC, months were compared within each variety when the month effect or the variety × month interaction was significant, varieties were compared within each month when the interaction was significant, and season means of the varieties were compared when the variety effect was significant. Results are presented as arithmetic means ± standard error (SE) of the trees (n = 6–9 per variety and month).

Because each tree was sampled repeatedly, relationships between leaf nutrients were assessed with repeated-measures correlation (r_rm_) [38]. This method estimates the common within-tree association between two nutrients and does not treat repeated samples from the same tree as independent observations. r_rm_ was calculated separately for Hass at Mojo and for each variety at MARC, using the same transformations as above, and the P values within each correlation matrix were adjusted with the Benjamini–Hochberg false discovery rate procedure [39]. Pearson correlations calculated on all tree × month observations are given in S2 Table for comparison.

Soil data are presented as values for each sampling point and as mean ± SE for each depth (n = 3 farms at Mojo, with the two points of farm 3 averaged first, and n = 3 points at MARC). Each soil property was rated against standard classes: pH, OC and EC as given in the Ethiopian guide [24], CEC and TN after Landon [40], and DTPA-extractable micronutrients after Lindsay and Norvell [31]. Because the two sites lie in different agroecological settings and their soils were not sampled under a common design, the soils were described for each site and not compared statistically. All analyses and figures were done in R version 4.3.3 [41] with the ggplot2 package [42]. The R scripts and the data are provided as S1 Code and S1 Dataset.

## Results

### Soil properties of the study sites

The Mojo soils were neutral to mildly alkaline (pH 7.24–7.85; Table 4), and CEC was high (30.7–39.8 cmol(+)/kg) at all farms. Total N was very low to low (0.05–0.12%). Organic carbon was low on farm 1 (0.70% and 0.64% at 0–20 and 20–50 cm) and in the subsoil at point P1 of farm 3 (1.10%), and medium elsewhere (1.56–1.74%). The soils were non-saline (EC 98.8–218.0 µS/cm). DTPA-extractable Cu was medium to high (0.62–0.90 mg/kg), Mn was low to medium (0.54–2.62 mg/kg), Zn was very low (0.09–0.42 mg/kg) at all sampling points, and Fe was below the detection limit in all samples (very low). Cu and Mn were higher in the 0–20 cm than in the 20–50 cm layer at all four sampling points. The soils were mainly clay loam, with clay texture at point P2 of farm 3.

**Table 4:** Soil properties of the commercial Hass orchards at Mojo.

Parameter	Farm 1		Farm 2		Farm 3 (P1)		Farm 3 (P2)		Mean ± SE (3 farms)		Rating
**Depth (cm)**	**0–20**	**20–50**	**0–20**	**20–50**	**0–20**	**20–50**	**0–20**	**20–50**	**0–20**	**20–50**	
pH	7.64	7.72	7.73	7.83	7.85	7.24	7.37	7.61	7.66 ± 0.04	7.66 ± 0.12	Neutral–mildly alkaline
EC (µS/cm)	132.2	117.9	178.2	218.0	98.8	142.9	132.7	188.1	142.1 ± 18.7	167.1 ± 28.9	Non-saline
CEC (cmol(+)/kg)	34.1	37.8	37.0	39.8	30.7	31.4	34.0	35.8	34.5 ± 1.3	37.1 ± 1.8	High
OC (%)	0.70	0.64	1.56	1.62	1.74	1.10	1.56	1.57	1.30 ± 0.30	1.20 ± 0.29	Low–medium
TN (%)	0.06	0.05	0.10	0.09	0.12	0.08	0.09	0.09	0.090 ± 0.013	0.077 ± 0.013	Very low–low
C:N ratio	10.8	12.3	15.7	17.2	14.1	13.6	17.6	17.8	14.1 ± 1.7	15.1 ± 1.5	
Cu (mg/kg)	0.90	0.79	0.90	0.73	0.74	0.66	0.72	0.62	0.84 ± 0.06	0.72 ± 0.05	Medium–high
Fe (mg/kg)	ND	ND	ND	ND	ND	ND	ND	ND	ND	ND	Very low
Mn (mg/kg)	2.08	0.54	1.66	0.88	2.49	1.54	2.62	0.78	2.10 ± 0.26	0.86 ± 0.18	Low–medium
Zn (mg/kg)	0.09	0.12	0.28	0.22	0.42	0.25	0.29	0.24	0.24 ± 0.08	0.19 ± 0.04	Very low
Sand (%)	25	22	31	29	24	25	15	15	25.2 ± 3.3	23.7 ± 2.7	
Clay (%)	31	38	27	33	32	33	49	49	32.8 ± 4.0	37.3 ± 2.3	
Silt (%)	44	40	42	38	44	42	36	36	42.0 ± 1.2	39.0 ± 0.6	
Textural class	CL	CL	L/CL	CL	CL	CL	C	C			

Values are from one sample per sampling point and depth; farm 3 was sampled at two points (P1 and P2). Mean ± SE of the three farms (the two points of farm 3 averaged). Ratings follow the Ethiopian guide [24] for pH, EC and OC, Landon [40] for CEC and TN, and Lindsay and Norvell [31] for Cu, Fe, Mn and Zn. EC = electrical conductivity; CEC = cation exchange capacity (cmol(+)/kg, equivalent to meq/100 g); OC = organic carbon; TN = total nitrogen; C:N = ratio of OC to TN; ND = below the detection limit; CL = clay loam; L/CL = loam to clay loam; C = clay.

The MARC soils were mildly to strongly alkaline (pH 7.72–8.50; Table 5), non-saline (EC 220–320 µS/cm) and coarser in texture than the Mojo soils (loamy sand to loam, with 49–84% sand). CEC ranged from medium to very high (20.3–40.8 cmol(+)/kg) and was lowest in the sandy topsoil at P1. Total N was very low to low (0.08–0.18%), and organic carbon was low to medium (1.24–1.66%). DTPA-extractable Cu (3.09–4.55 mg/kg) and Mn (6.43–8.28 mg/kg) were very high, Fe was low (2.28–2.65 mg/kg), and Zn was very low at P1 and P2 (trace to 0.38 mg/kg) and medium at P3 (1.38–1.44 mg/kg).

**Table 5:** Soil properties of the MARC avocado orchard. Values are from one sample per sampling point and depth (three points, P1–P3, in the orchard); mean ± SE of the three points. Ratings as in Table 4. EC = electrical conductivity; CEC = cation exchange capacity; OC = organic carbon; TN = total nitrogen; C:N = ratio of OC to TN; Tr = trace (below the quantification limit); LS = loamy sand; SCL = sandy clay loam; SL = sandy loam; L = loam. ^a^ Trace values were set to half the lowest quantified value (0.095 mg/kg) to calculate the mean.

Parameter	P1		P2		P3		Mean ± SE (3 points)		Rating
**Depth (cm)**	**0–20**	**20–50**	**0–20**	**20–50**	**0–20**	**20–50**	**0–20**	**20–50**	
pH	8.50	8.50	7.79	7.82	7.72	8.06	8.00 ± 0.25	8.13 ± 0.20	Mildly–strongly alkaline
EC (µS/cm)	250	320	220	250	240	240	237 ± 9	270 ± 25	Non-saline
CEC (cmol(+)/kg)	20.3	32.1	40.8	37.6	23.4	30.3	28.2 ± 6.4	33.3 ± 2.2	Medium–very high
OC (%)	1.51	1.29	1.41	1.24	1.66	1.52	1.53 ± 0.07	1.35 ± 0.09	Low–medium
TN (%)	0.12	0.08	0.18	0.13	0.15	0.13	0.150 ± 0.017	0.113 ± 0.017	Very low–low
C:N ratio	12.6	16.1	7.8	9.5	11.1	11.7	10.5 ± 1.4	12.4 ± 1.9	
Cu (mg/kg)	3.15	3.80	3.18	3.09	4.55	4.55	3.63 ± 0.46	3.81 ± 0.42	Very high
Fe (mg/kg)	2.65	2.37	2.28	2.42	2.65	2.51	2.53 ± 0.12	2.43 ± 0.04	Low
Mn (mg/kg)	8.28	6.43	7.50	6.45	6.80	7.31	7.53 ± 0.43	6.73 ± 0.29	Very high
Zn (mg/kg)	0.38	0.19	Tr	Tr	1.44	1.38	0.64 ± 0.41^a^	0.56 ± 0.41^a^	Very low–medium
Sand (%)	84	62	66	62	49	51	66.3 ± 10.1	58.3 ± 3.7	
Clay (%)	6	22	12	14	8	14	8.7 ± 1.8	16.7 ± 2.7	
Silt (%)	10	16	22	24	43	35	25.0 ± 9.6	25.0 ± 5.5	
Textural class	LS	SCL	SL	SL	L	L			

### Mojo site: Seasonal changes in Hass leaf nutrients

At Mojo, leaf TN (1.30–1.52%) and Ca (1.09–1.27%) did not change significantly between February and June (Table 6; Fig 2). Cu was below the detection limit in all trees in February and then remained at 9.7–11.0 mg/kg from March to June (P < 0.001). Mn was lowest in February (108 mg/kg) and significantly higher in March and June (155–173 mg/kg; P = 0.005). Zn was about twice as high in March (36.9 mg/kg) as in the other months (14.8–17.0 mg/kg; P < 0.001). Fe varied widely between trees (from below the detection limit to 445 mg/kg), and the month effect was not significant (P = 0.051), although the rank-based check suggested a month effect (P = 0.010).

**Table 6:** Leaf nutrient concentrations of Hass avocado at Mojo in each sampling month. Values are mean ± SE (n = 9 trees). Letters are given only for nutrients with a significant month effect; within a column, means followed by the same letter are not significantly different (Tukey’s test, P < 0.05). TN, Fe and Zn were analysed on the log scale. ND = below the detection limit in all trees (the substituted value of 0.20 mg/kg was used in the analysis).

Month	TN (%)	Ca (%)	Cu (mg/kg)	Fe (mg/kg)	Mn (mg/kg)	Zn (mg/kg)
February	1.52 ± 0.14	1.16 ± 0.06	ND b	59.5 ± 6.4	108.2 ± 13.3 b	14.84 ± 1.75 b
March	1.46 ± 0.10	1.23 ± 0.11	9.69 ± 1.14 a	58.0 ± 8.1	172.7 ± 27.3 a	36.94 ± 6.79 a
May	1.30 ± 0.07	1.27 ± 0.10	10.02 ± 1.15 a	81.2 ± 47.9	150.6 ± 18.0 ab	17.02 ± 1.02 b
June	1.36 ± 0.09	1.09 ± 0.09	10.96 ± 0.76 a	77.2 ± 9.8	155.0 ± 11.3 a	16.75 ± 2.05 b
Mean (Feb–Jun)	1.41 ± 0.05	1.19 ± 0.05	7.72 ± 0.85	69.0 ± 12.1	146.6 ± 9.7	21.39 ± 2.33
Month effect, P	F_3,24_ = 1.09; P = 0.372	F_3,24_ = 1.34; P = 0.285	F_3,24_ = 43.20; P < 0.001	F_3,24_ = 3.00; P = 0.051	F_3,24_ = 5.63; P = 0.005	F_3,24_ = 16.12; P < 0.001

**Fig 2:**
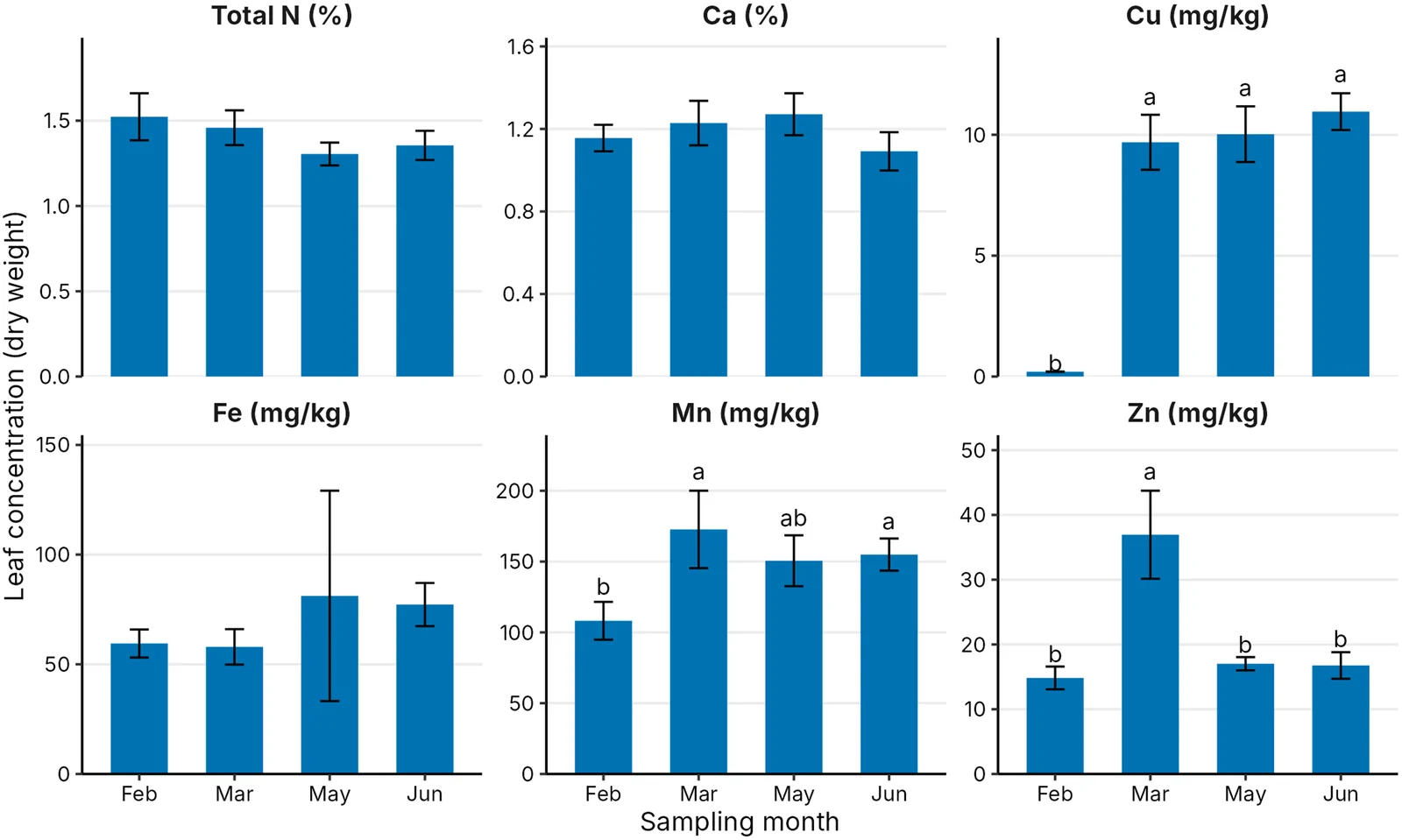
Seasonal changes in leaf nutrient concentrations of Hass avocado at Mojo (February–June). Bars are means and error bars are ± SE (n = 9 trees). Letters are shown only for nutrients with a significant month effect (F-test, P < 0.05); within a panel, months with the same letter are not significantly different (Tukey’s test, P < 0.05; Zn was tested on log-transformed data). Cu was below the detection limit in all trees in February.

### Melkassa (MARC) site: Seasonal and varietal changes in leaf nutrients

At MARC, leaf TN did not differ significantly between months (P = 0.93) or varieties (P = 0.079), and there was no variety × month interaction (P = 0.36) (Table 7; Fig 3). Averaged over the season, TN was 1.33 ± 0.06% in Hass, 1.27 ± 0.05% in Fuerte and 1.13 ± 0.03% in Ettinger. Leaf Ca increased steadily in all three varieties, from 0.79–1.00% in February to 1.61–1.65% in July (month effect, P < 0.001), with no difference between varieties (P = 0.29) and no interaction (P = 0.53).

**Table 7:** Leaf nutrient concentrations of three avocado varieties at MARC in each sampling month. Values are mean ± SE (n = 6–9 trees). Lowercase letters compare months within a variety (column); uppercase letters compare varieties within a month (row); means sharing a letter are not significantly different (Tukey’s test, P < 0.05). Letters are given only where the corresponding F-test was significant (P < 0.05). The last row of each nutrient gives the P values of the mixed-model F-tests for variety (V), month (M) and their interaction (V × M). TN, Fe and Zn were analysed on the log scale. ND = below the detection limit in all trees (the substituted value of 0.20 mg/kg was used in the analysis).

Nutrient	Month	Hass	Fuerte	Ettinger
TN (%)	February	1.34 ± 0.15	1.25 ± 0.04	1.18 ± 0.06
	March	1.32 ± 0.08	1.38 ± 0.17	1.04 ± 0.09
	May	1.48 ± 0.19	1.15 ± 0.06	1.14 ± 0.05
	June	1.20 ± 0.09	1.25 ± 0.06	1.14 ± 0.05
	July	1.29 ± 0.12	1.36 ± 0.16	1.14 ± 0.08
	Season mean	1.33 ± 0.06	1.27 ± 0.05	1.13 ± 0.03
		P: V = 0.079; M = 0.926; V × M = 0.358		
Ca (%)	February	1.00 ± 0.06 c	0.79 ± 0.06 c	1.00 ± 0.10 c
	March	1.23 ± 0.15 bc	1.03 ± 0.04 bc	1.18 ± 0.08 bc
	May	1.26 ± 0.05 bc	1.33 ± 0.12 ab	1.24 ± 0.12 bc
	June	1.51 ± 0.08 ab	1.27 ± 0.10 ab	1.37 ± 0.06 ab
	July	1.65 ± 0.06 a	1.61 ± 0.15 a	1.61 ± 0.07 a
	Season mean	1.34 ± 0.05	1.19 ± 0.06	1.28 ± 0.05
		P: V = 0.292; M < 0.001; V × M = 0.531		
Cu (mg/kg)	February	1.30 ± 0.55 cA	ND bA	0.36 ± 0.16 cA
	March	ND cB	ND bB	7.07 ± 0.80 abA
	May	7.91 ± 0.94 bA	7.71 ± 0.86 aA	7.80 ± 0.94 abA
	June	5.78 ± 1.00 bB	7.76 ± 0.64 aAB	8.84 ± 1.25 aA
	July	11.40 ± 0.86 aA	9.09 ± 1.53 aA	5.47 ± 1.02 bB
	Season mean	5.53 ± 0.72	4.80 ± 0.68	5.91 ± 0.59
		P: V = 0.223; M < 0.001; V × M < 0.001		
Fe (mg/kg)	February	74.5 ± 14.8 aA	76.7 ± 9.6 aA	115.5 ± 29.8 aA
	March	115.4 ± 11.8 aA	88.0 ± 4.9 aA	107.0 ± 3.6 aA
	May	55.8 ± 6.5 aA	43.7 ± 8.0 aA	32.6 ± 10.7 bB
	June	24.3 ± 9.5 bB	10.8 ± 6.0 bB	146.3 ± 81.6 aA
	July	128.2 ± 9.0 aA	128.4 ± 31.6 aA	109.4 ± 13.4 aA
	Season mean	78.9 ± 7.4	66.8 ± 8.2	102.2 ± 17.8
		P: V = 0.184; M < 0.001; V × M < 0.001		
Mn (mg/kg)	February	75.8 ± 7.2 bA	62.1 ± 4.3 bA	78.6 ± 12.1 cA
	March	89.3 ± 16.9 bB	64.2 ± 6.3 bB	121.2 ± 8.9 abA
	May	122.5 ± 12.3 aAB	144.3 ± 10.2 aA	93.6 ± 9.3 bcB
	June	130.4 ± 7.6 aA	116.1 ± 6.4 aA	139.2 ± 4.5 aA
	July	128.2 ± 6.6 aA	126.1 ± 9.4 aA	135.6 ± 9.2 aA
	Season mean	110.5 ± 5.7	100.9 ± 6.2	113.6 ± 5.3
		P: V = 0.358; M < 0.001; V × M < 0.001		
Zn (mg/kg)	February	15.65 ± 1.41 aA	14.63 ± 0.82 aA	12.63 ± 1.79 aA
	March	6.00 ± 1.52 bB	2.29 ± 0.89 bC	15.85 ± 3.65 aA
	May	15.94 ± 1.24 aA	22.37 ± 5.04 aA	18.51 ± 2.60 aA
	June	16.38 ± 1.52 aA	17.93 ± 2.57 aA	22.96 ± 11.18 aA
	July	13.28 ± 1.50 aA	13.06 ± 1.84 aA	11.86 ± 0.91 aA
	Season mean	13.57 ± 0.85	14.13 ± 1.62	16.36 ± 2.41
		P: V = 0.069; M < 0.001; V × M < 0.001		

**Fig 3:**
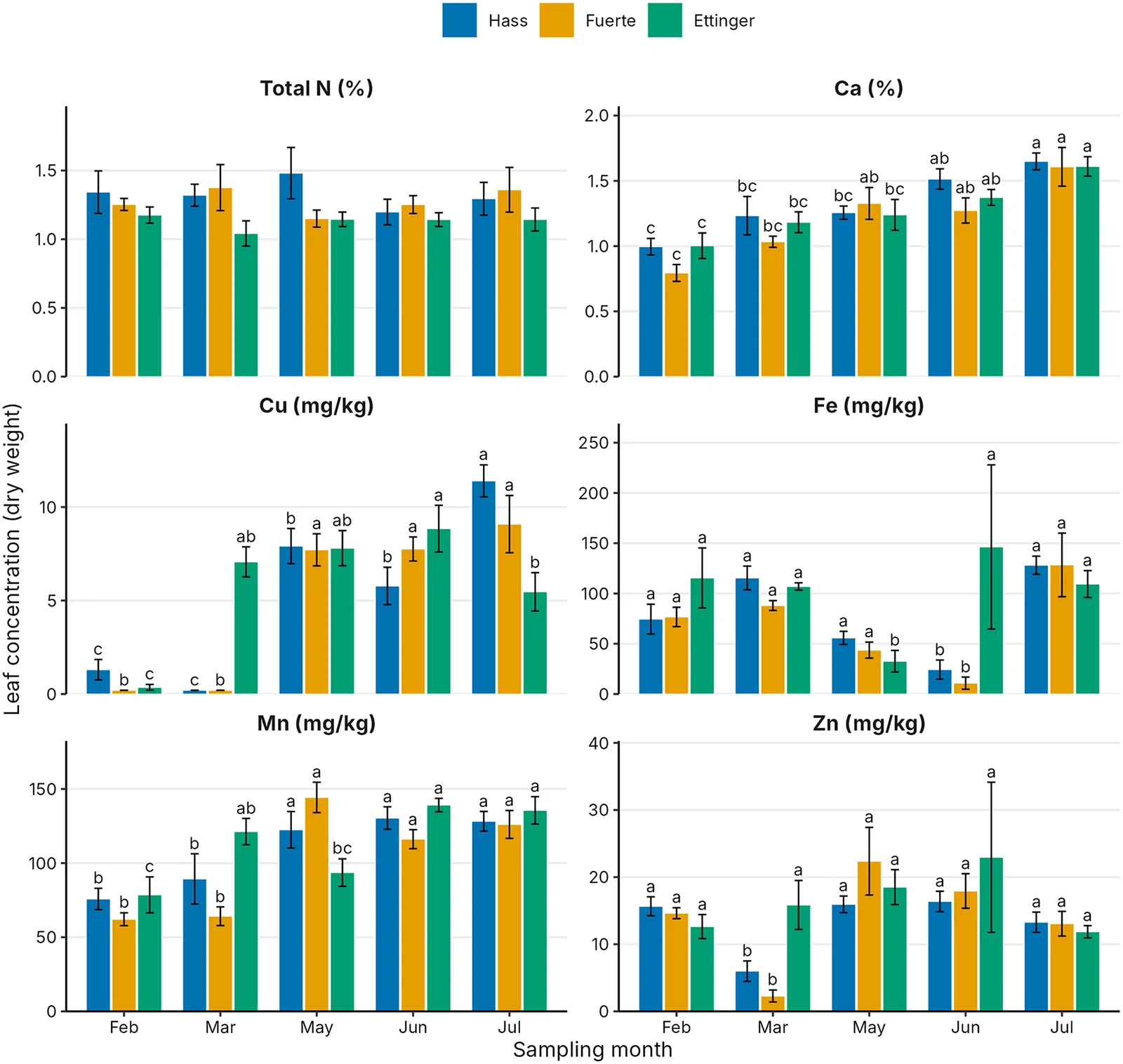
Seasonal changes in leaf nutrient concentrations of Hass, Fuerte and Ettinger avocado at MARC (February–July). Bars are means and error bars are ± SE (n = 6–9 trees). Letters are shown only where the month or variety × month effect was significant (F-test, P < 0.05); within each variety, months with the same letter are not significantly different (Tukey’s test, P < 0.05; Fe and Zn were tested on log-transformed data). Comparisons between varieties within each month are given in Table 7.

All four micronutrients showed significant variety × month interactions (P < 0.001), which means that the seasonal pattern differed between varieties (Table 7; Fig 3). Mn was lowest in February (62–79 mg/kg) and reached 116–139 mg/kg in June–July; Fuerte already peaked in May (144 mg/kg), whereas Ettinger had more Mn than the other varieties in March and less than Fuerte in May. Cu was below or close to the detection limit in February in all varieties and also in March in Hass and Fuerte, but not in Ettinger (7.1 mg/kg). From May onwards, Cu was 5.5–11.4 mg/kg, with the highest values in Hass and Fuerte in July. Fe was lowest in June in Hass (24 mg/kg) and Fuerte (11 mg/kg) and in May in Ettinger (33 mg/kg), and highest in February, March and July (74–128 mg/kg); the high mean for Ettinger in June was caused by one tree with 790 mg/kg. Zn dropped sharply in March in Hass (6.0 mg/kg) and Fuerte (2.3 mg/kg) but not in Ettinger (15.9 mg/kg), and was otherwise 11.9–23.0 mg/kg. Averaged over the season, none of the six nutrients differed significantly between the three varieties (Table 7); only for Fe did the rank-based check suggest a difference between varieties (P = 0.013).

### Hass leaf nutrients at Mojo and MARC compared

Over the four months sampled at both sites (February–June), Hass leaves at Mojo contained more Mn (146.6 ± 9.7 vs 105.8 ± 6.8 mg/kg; P = 0.029), Cu (7.7 ± 0.9 vs 4.0 ± 0.7 mg/kg; P < 0.001) and Zn (21.4 ± 2.3 vs 13.7 ± 1.0 mg/kg; P = 0.008) than Hass leaves at MARC (Fig 4). These differences were largest in March, and for Cu also in June (site × month interaction, P ≤ 0.02). TN (1.41 ± 0.05 vs 1.34 ± 0.07%; P = 0.37), Ca (P = 0.51) and Fe (P = 0.65) did not differ between the sites overall, but in June Ca was lower (1.09 vs 1.51%; P = 0.002) and Fe higher (77 vs 24 mg/kg; P < 0.001) at Mojo than at MARC. The two sites differ in tree age (planted in 2021 at Mojo and in 2001 at MARC), agroecology and soil, so these differences cannot be attributed to a single factor.

**Fig 4:**
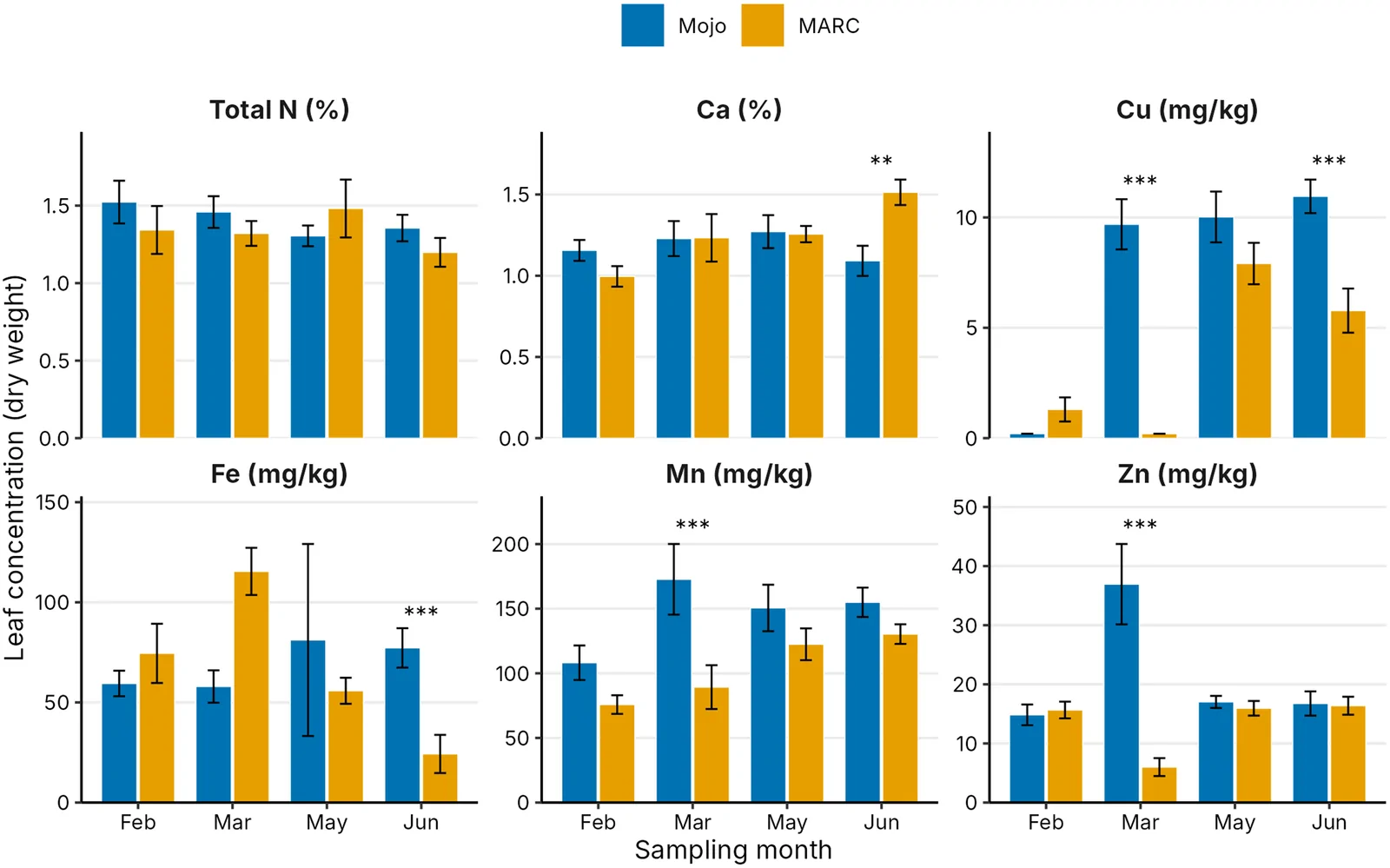
Leaf nutrient concentrations of Hass avocado at Mojo and MARC in the four months sampled at both sites. Bars are means and error bars are ± SE (n = 8–9 trees). Asterisks show months in which the two sites differed significantly (* P < 0.05, ** P < 0.01, *** P < 0.001; mixed-model contrasts; TN, Fe and Zn were tested on log-transformed data).

### Leaf nutrient levels compared with published sufficiency ranges

Mean leaf TN (1.13–1.41%) was below the optimum range of 1.6–2.2% in all varieties at both sites (Table 8), and only 17 of the 167 individual samples reached 1.6%. Mean Ca (1.19–1.34%) and Mn (101–147 mg/kg) were within the optimum ranges. Mean Fe (67–102 mg/kg) was within the 50–200 mg/kg range, but the monthly means at MARC fell below 50 mg/kg in May or June in all three varieties. Mean leaf Zn (13.6–21.4 mg/kg) was below the sufficiency range of 30–150 mg/kg [9]; 82% of the Zn values were below 20 mg/kg, and only 9 of 166 samples reached 30 mg/kg.

**Table 8:** Leaf nutrient concentrations in the present study compared with published optimum ranges. Values are mean ± SE of all tree × month observations (Mojo: February–June; MARC: February–July), with the range of the monthly means in brackets. NA = not available.

Nutrient	Unit	Optimum range	References	Hass, Mojo	Hass, MARC	Fuerte, MARC	Ettinger, MARC
TN	%	1.6–2.2	[6,9,16,43,44]	1.41 ± 0.05 (1.30–1.52)	1.33 ± 0.06 (1.20–1.48)	1.27 ± 0.05 (1.15–1.38)	1.13 ± 0.03 (1.04–1.18)
Ca	%	1–3	[6,9,16,43,44]	1.19 ± 0.05 (1.09–1.27)	1.34 ± 0.05 (1.00–1.65)	1.19 ± 0.06 (0.79–1.61)	1.28 ± 0.05 (1.00–1.61)
Fe	mg/kg	50–200	[9,16,43]	69.0 ± 12.1 (58.0–81.2)	78.9 ± 7.4 (24.3–128.2)	66.8 ± 8.2 (10.8–128.4)	102.2 ± 17.8 (32.6–146.3)
Mn	mg/kg	30–500	[9,16,43]	146.6 ± 9.7 (108.2–172.7)	110.5 ± 5.7 (75.8–130.4)	100.9 ± 6.2 (62.1–144.3)	113.6 ± 5.3 (78.6–139.2)
Cu	mg/kg	NA	–	7.72 ± 0.85 (0.20–10.96)	5.53 ± 0.72 (0.20–11.40)	4.80 ± 0.68 (0.20–9.09)	5.91 ± 0.59 (0.36–8.84)
Zn	mg/kg	30–150	[9,16,43]	21.39 ± 2.33 (14.84–36.94)	13.57 ± 0.85 (6.00–16.38)	14.13 ± 1.62 (2.29–22.37)	16.36 ± 2.41 (11.86–22.96)

### Relationships among leaf nutrients

Within trees, Mn was positively correlated with Ca in all four variety–site groups (r_rm_ = 0.50–0.77; adjusted P < 0.05), with Cu in Hass at both sites and in Fuerte (r_rm_ = 0.54–0.76), and with Zn in Hass at both sites and in Fuerte (r_rm_ = 0.47–0.52) (Fig 5; S2 Table). Cu was also positively correlated with Ca in Hass and Fuerte at MARC (r_rm_ = 0.54 and 0.61) and with Zn in Fuerte (r_rm_ = 0.53). The only significant negative correlation was between TN and Ca in Fuerte (r_rm_ = −0.40). TN was not significantly correlated with Zn in any group (r_rm_ = −0.08 to 0.11), and Fe was not significantly correlated with Zn (r_rm_ = −0.33 to 0.14).

**Fig 5:**
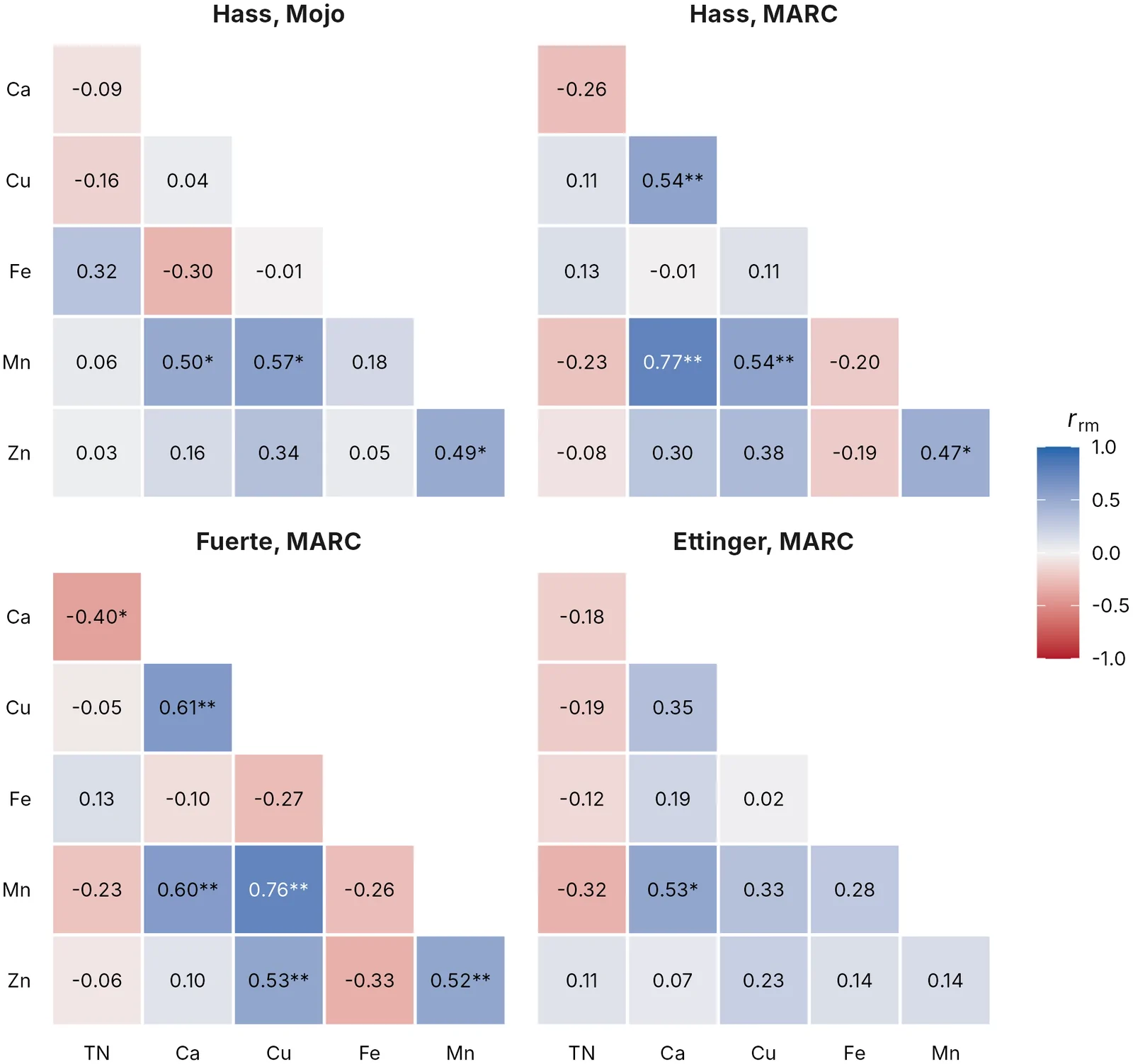
Repeated-measures correlations among leaf nutrients within trees. Coefficients (r_rm_) for Hass at Mojo (36 observations from 9 trees) and Hass, Fuerte and Ettinger at MARC (42–45 observations from 9 trees each). TN, Fe and Zn were log-transformed. * and ** show correlations that are significant at P < 0.05 and P < 0.01 after Benjamini–Hochberg adjustment within each group.

## Discussion

### Soil properties of the study sites

The soils of both sites were neutral to alkaline. At this pH, Fe, Zn and Cu form less soluble compounds and their availability to plants decreases [45,46], which is consistent with the very low (Mojo) to low (MARC) DTPA-extractable Fe and the very low Zn at Mojo. Leaf Fe was nevertheless within the optimum range on average, but it varied greatly between trees and fell below 50 mg/kg in May–June at MARC, so leaf Fe should be monitored. The clay-rich Mojo soils had a high CEC and the sandier MARC soils a lower and more variable CEC, consistent with their clay contents [47,48]; both soils were non-saline [49].

Within each site, the soil and leaf results agreed for N and Zn. Soil TN was very low to low at both sites, and leaf TN was below the sufficiency range in all varieties. Soil Zn was very low at all Mojo sampling points and at two of the three MARC points, and mean leaf Zn was below the sufficiency range at both sites (Table 8). Zn is essential for enzyme function and protein synthesis [14,15], so N and Zn supply are the most likely nutrient constraints in these orchards. Our second hypothesis, that soil nutrient status is reflected in the leaves, was therefore supported for N and Zn, but not for Fe. For Cu and Mn, a single soil sampling could not be related directly to leaf concentrations that changed strongly during the season.

### Seasonal and varietal patterns in leaf nutrients

Leaf TN was below the published sufficiency range (1.6–2.2%) in all varieties and at both sites throughout the sampling period [9,16,43], which suggests that both orchards were short of N during the study. TN did not change significantly between February and July, and the differences between varieties (Hass > Fuerte > Ettinger) and between sites were small and not statistically significant. The low N status therefore appears to be a general feature of these orchards rather than a seasonal or varietal effect. Nitrogen is mobile in the plant and is redistributed from older leaves to new growth [50,51]; part of the variation between trees may therefore reflect differences in flushing and fruit load rather than N supply.

Leaf Ca increased steadily from February to July in all three varieties at MARC. Ca moves to the leaves with the transpiration stream and is hardly remobilized, so it accumulates as leaves age [52,53]. The increase therefore mainly reflects leaf age, and this should be taken into account when leaf Ca is interpreted: a sample taken in February contained about 40–50% less Ca than a sample from the same tree in July. At Mojo, Ca did not increase between February and June, which may relate to the much younger age of the Mojo trees.

The micronutrients showed clear seasonal patterns that differed between varieties. Mn increased from February towards June–July, and Cu was below or near the detection limit in February (and in March in Hass and Fuerte at MARC) but reached 5–11 mg/kg from May onwards. The low early-season values coincided with flowering and the spring flush, when nutrients are diluted in rapidly expanding tissues and allocated to flowers [7,44]. The sharp drop in Zn in March in Hass and Fuerte at MARC, but not in Ettinger or at Mojo, and the low Fe in May–June at MARC show that a single leaf sample can give a misleading picture of micronutrient status if the sampling month is not standardized. Leaf samples for diagnosis should therefore be taken at the same phenological stage each year, as recommended for Hass avocado [6]. Overall, our first hypothesis was supported for the micronutrients, whose seasonal patterns differed between varieties, and partly for Ca, which changed with season at MARC in the same way in all varieties; it was not supported for TN.

Hass trees at Mojo had higher leaf Mn, Cu and Zn than Hass trees at MARC, mainly in March. The two sites differ in tree age, agroecology and soil, so the cause of these differences cannot be determined from this study. Because varieties were compared only at MARC and sites only for Hass, the results should be interpreted as site-specific.

### Relationships among leaf nutrients

Repeated-measures correlation showed that, within trees, Mn changed together with Ca in all variety–site groups and with Cu and Zn in Hass and Fuerte. Because Ca, Mn and Cu all increased during the season, part of these positive correlations reflects a common seasonal trend (leaf ageing and growth stage) rather than a direct physiological interaction. These nutrients may also interact during uptake and transport [54,55], but the correlations show only which nutrients change together in the leaf, not that one nutrient improves the uptake of another.

We found no evidence of a negative correlation between TN and Zn or between Zn and Fe in avocado leaves within the range of concentrations observed; neither correlation was significant in any variety or at either site. The only significant negative correlation, between TN and Ca in Fuerte (r_rm_ = −0.40), was weak and was not found in the other varieties. Because the same trees were sampled repeatedly, such relationships should be analysed within trees rather than from monthly means [38].

### Implications for nutrient management

The results have three practical implications. First, leaf TN was below the sufficiency range at both sites and soil TN was very low to low at both sites, so N fertilization should be reviewed in both orchards, with split applications timed to the main growth stages [7]. Second, leaf Zn was below the sufficiency range at both sites, and the soils at both sites were neutral to alkaline, with low DTPA-extractable Fe and mostly low Zn; foliar sprays or soil application of chelated Zn and Fe may be needed, and leaf Zn and Fe should be monitored [7,9]. Third, Ca, Mn, Cu, Fe and Zn changed strongly with season, and some patterns differed between varieties; leaf samples for diagnosis should therefore be collected at a fixed phenological stage, and fertilization should be adjusted to the variety and site rather than following a uniform strategy. Combining organic and inorganic nutrient sources can improve soil organic carbon, nutrient retention and overall soil health, particularly on fields with low organic carbon such as farm 1 at Mojo [56].

This study has some limitations. Leaf sampling covered one production season, from the flush in February to July, which spans the main phenological stages from flowering to fruit maturity; April was not sampled, and Mojo was not sampled in July because the fruit had reached maturity. The soil data were limited: the soils were sampled once, with one sample per sampling point and depth on three farms at Mojo and at three points at MARC, and the soils of the two sites were therefore described but not compared statistically. Tree age differed greatly between the sites, and varieties could be compared only at MARC because the Mojo farmers grow only Hass. Some Cu, Fe and Zn values were below the detection limit, and fruit yield and quality were not measured. Repeating the sampling in further seasons at both sites, linked to yield, would help to confirm seasonal reference values for these varieties.

## Conclusion

Leaf TN was below the sufficiency range in all varieties at both sites and did not differ significantly between months, varieties or sites, and leaf Zn was below the sufficiency range in most samples. Leaf Ca increased strongly from February to July in all varieties at MARC, whereas Cu, Fe, Mn and Zn showed variety-specific seasonal patterns. Hass at Mojo had higher leaf Mn, Cu and Zn than Hass at MARC. Within trees, Mn was positively correlated with Ca in all varieties and with Cu and Zn in Hass and Fuerte. Soils at both sites were neutral to alkaline and very low to low in TN, and within each site the soil and leaf data agreed in showing a low N and Zn status.

These findings imply that N and Zn supply should be improved in both orchards, that leaf Fe should be monitored on the alkaline soils of both sites, and that leaf samples for nutrient diagnosis must be taken at a standard time of year because several nutrients change strongly with season. Future work should cover a full year at both sites and link leaf nutrient levels with yield, fruit quality and nutrient removal at harvest to define annual nutrient requirements.

## Supporting information

S1 Table Mixed-model analysis of variance for leaf nutrients. F values, numerator and denominator degrees of freedom (Kenward–Roger) and P values for all analyses, with the residual checks on the original and log scales, the rank-based (aligned rank transform) check for Fe and Zn, and the sensitivity checks for values below the detection limit and for the farm effect at Mojo.
(XLSX)

S2 Table Correlations among leaf nutrients. Repeated-measures correlation coefficients (r_rm_) with degrees of freedom, number of observations, P values and Benjamini–Hochberg adjusted P values for each variety–site group, and Pearson correlations calculated on all tree × month observations.
(XLSX)

S1 Dataset Tree-level leaf nutrient data and soil data. Leaf TN, Ca, Cu, Fe, Mn and Zn of each tagged tree in each sampling month (ND = below detection limit), soil properties of the Mojo farms and the MARC orchard points, and the summary tables used for the figures.
(XLSX)

S1 Code R scripts. Scripts used for the mixed models, mean separation, repeated-measures correlations, the rank-based check for Fe and Zn, the checks of the substitution of values below the detection limit and of the farm effect at Mojo, the soil summaries and Figs 2–5.
(ZIP)
