# Supplementary material for: Seasonal dynamics of leaf macro- and micronutrients in three commercial avocado varieties at Mojo and Melkassa, Ethiopia
Source: PLoS One. 2026 Nov 12;21(11):e0362564. doi: 10.1371/journal.pone.0362564 (PMC13759051; doi:10.1371/journal.pone.0362564)
Supplement: Supplementary file 1 [file pone.0362564.s005.pdf]

PLOS One

Levels and seasonal patterns of selected essential macro and micronutrients in commercial avocado varieties and soil nutrient status of avocado farms at Mojo and Melkassa sites, Ethiopia

--Manuscript Draft--

**Manuscript Number:**	PONE-D-26-25421
**Article Type:**	Research Article
**Full Title:**	Levels and seasonal patterns of selected essential macro and micronutrients in commercial avocado varieties and soil nutrient status of avocado farms at Mojo and Melkassa sites, Ethiopia
**Short Title:**	Seasonal leaf nutrient pattern in avocado varieties.
**Corresponding Author:**	Zeyede Aregahegn Wondimgezahu, MSc.<br>Ethiopian Institute of Agricultural Research<br>Adama, ETHIOPIA
**Keywords:**	Avocado leaf analysis; nutrient dynamics; soil fertility; MARC and Moj; sampling months
**Abstract:**	Avocado fruit, rich in essential nutrients and highly productive, is a recommended crop for combating malnutrition. This study examines the levels of essential macro- and micronutrients, seasonal nutrient patterns in commercial avocado varieties, and the soil nutrient status of avocado farms in Mojo and Melkassa, Ethiopia. Soil samples from Mojo avocado farms revealed high cation exchange capacity (31.05–38.40 mEq/100 g soil), slightly alkaline pH (7.49–7.78), low total nitrogen (TN, 0.06–0.10%), low to medium organic carbon (0.67–1.59%), medium copper and manganese levels (0.67–0.85 ppm and 1.27–2.02 ppm, respectively), and critically low iron and zinc levels (0 ppm and 0.10–0.34 ppm, respectively). Leaf tissue analysis showed that nitrogen levels were highest in the Hass variety, followed by Fuerte and Ettinger. At the Mojo site, nitrogen levels in Hass leaf samples declined from February to July, while remaining consistent at the MARC site. Pearson correlation analysis revealed synergistic relationships among manganese, copper, and calcium, as well as antagonistic interactions between nitrogen and zinc and competition between zinc and iron. These findings underscore the importance of optimizing nutrient management to promote healthy avocado growth and productivity. Practical implications include the need for targeted interventions to address deficiencies in zinc and iron in avocado soils, coupled with continuous leaf nutrient monitoring to align fertilization strategies with seasonal and varietal nutrient dynamics. Future studies correlating nutrient data with fruit yield, quality, and shelf life will provide valuable insights for maximizing avocado productivity and sustainability.
**Order of Authors:**	Zeyede Aregahegn Wondimgezahu, MSc.
**Additional Information:**	
**Question**	**Response**
**Financial Disclosure**<br><br>Enter a financial disclosure statement that describes the sources of funding for the work included in this submission. Review the submission guidelines for detailed requirements. View published research articles from *PLOS ONE* for specific examples.<br><br>This statement is required for submission	The author(s) received no specific funding for this work.

Powered by Editorial Manager® and ProduXion Manager® from Aries Systems Corporation

and **will appear in the published article** if the submission is accepted. Please make sure it is accurate.<br><br>**Funded studies**<br>Enter a statement with the following details:<br>• Initials of the authors who received each award<br>• Grant numbers awarded to each author<br>• The full name of each funder<br>• URL of each funder website<br>• Did the sponsors or funders play any role in the study design, data collection and analysis, decision to publish, or preparation of the manuscript?<br><br>Did you receive funding for this work?	
**Competing Interests**<br><br>Use the instructions below to enter a competing interest statement for this submission. On behalf of all authors, disclose any competing interests that could be perceived to bias this work—acknowledging all financial support and any other relevant financial or non-financial competing interests.<br><br>This statement is required for submission and **will appear in the published article** if the submission is accepted. Please make sure it is accurate and that any funding sources listed in your Funding Information later in the submission form are also declared in your Financial Disclosure statement.<br><br>View published research articles from *PLOS ONE* for specific examples.	The authors have declared that no competing interests exist.

Powered by Editorial Manager® and ProduXion Manager® from Aries Systems Corporation

**NO authors have competing interests**<br><br>Enter: *The authors have declared that no competing interests exist.*<br><br>**Authors with competing interests**<br><br>Enter competing interest details beginning with this statement:<br><br>*I have read the journal's policy and the authors of this manuscript have the following competing interests: [insert competing interests here]*<br><br>* typeset	
**Ethics Statement**<br><br>Enter an ethics statement for this submission. This statement is required if the study involved:<br><br>• Human participants<br>• Human specimens or tissue<br>• Vertebrate animals or cephalopods<br>• Vertebrate embryos or tissues<br>• Field research<br><br>Write "N/A" if the submission does not require an ethics statement.<br><br>General guidance is provided below. Consult the submission guidelines for detailed instructions. **Make sure that all information entered here is included in the Methods section of the manuscript.**	Field: On-farm (farmer's field and on-station (MARC) field<br>Institute: Ethiopian Institute of Agricultural Research (EIAR), Melkassa Agricultural Research Center (MARC)

Powered by Editorial Manager® and ProduXion Manager® from Aries Systems Corporation

**Format for specific study types**<br><br>**Human Subject Research (involving human participants and/or tissue)**<br>• Give the name of the institutional review board or ethics committee that approved the study<br>• Include the approval number and/or a statement indicating approval of this research<br>• Indicate the form of consent obtained (written/oral) or the reason that consent was not obtained (e.g. the data were analyzed anonymously)<br><br>**Animal Research (involving vertebrate animals, embryos or tissues)**<br>• Provide the name of the Institutional Animal Care and Use Committee (IACUC) or other relevant ethics board that reviewed the study protocol, and indicate whether they approved this research or granted a formal waiver of ethical approval<br>• Include an approval number if one was obtained<br>• If the study involved *non-human primates*, add *additional details* about animal welfare and steps taken to ameliorate suffering<br>• If anesthesia, euthanasia, or any kind of animal sacrifice is part of the study, include briefly which substances and/or methods were applied<br><br>**Field Research**<br><br>Include the following details if this study involves the collection of plant, animal, or other materials from a natural setting:<br>• Field permit number<br>• Name of the institution or relevant body that granted permission	
**Data Availability**<br><br>Before publication, Authors are required to make fully available and without restriction all data underlying their findings. Sharing the data underlying your findings increases the visibility, credibility, and impact of your work, while supporting transparency and future scientific discovery. Please see our PLOS Data	For Study Protocols: No datasets were generated or analysed during the current study. All relevant data from this study will be made available upon study completion.

Policy page for detailed information on this policy.<br><br>A **Data Availability Statement**, detailing where the data can be accessed, is required at first submission. Insert your Data Availability Statement in the box below.<br><br>Please see the data reporting section of our submission guidelines for instructions on what you need to include in your Data Availability Statement. Examples of acceptable Data Availability Statements are included here for reference:<br>1. The minimal data set [and accompanying code, where generated] is available at [repository name] via [DOI hyperlink]<br>2. No data was generated by this study. The following existing data sources were used: [dataset name] from [dataset location] available via [web address or DOI hyperlink].<br>3. For Study Protocols: No datasets were generated or analysed during the current study. All relevant data from this study will be made available upon study completion.<br><br>*PLOS allows rare exemptions to address legal and ethical concerns. If you have legal or ethical restrictions, please use the box below to detail these in full sentences for the Journal team to consider.*<br><br>This statement will be typeset if the manuscript is accepted for publication.	

Powered by Editorial Manager® and ProduXion Manager® from Aries Systems Corporation

Manuscript

Click here to access/download;Manuscript;Seasonal avocado varieties leaf nutrients pattern....pdf

Levels and seasonal patterns of selected essential macro and micronutrients in commercial avocado varieties and soil nutrient status of avocado farms at Mojo and Melkassa sites, Ethiopia

Zeyede Aregahegn*, Edossa Etissa, Israel Bekele, Dejene Abera

Ethiopian Institute of Agricultural Research, Melkassa Agricultural Research Center

Corresponding author: Zeyede Aregahegn

Email: zeyedearegahegn@gmail.com

Orchid ID: 0000-0001-8931-9234

Abstract

Avocado fruit, rich in essential nutrients and highly productive, is a recommended crop for combating malnutrition. This study examines the levels of essential macro- and micronutrients, seasonal nutrient patterns in commercial avocado varieties, and the soil nutrient status of avocado farms in Mojo and Melkassa, Ethiopia. Soil samples from Mojo avocado farms revealed high cation exchange capacity (31.05–38.40 mEq/100 g soil), slightly alkaline pH (7.49–7.78), low total nitrogen (TN, 0.06–0.10%), low to medium organic carbon (0.67–1.59%), medium copper and manganese levels (0.67–0.85 ppm and 1.27–2.02 ppm, respectively), and critically low iron and zinc levels (0 ppm and 0.10–0.34 ppm, respectively). Leaf tissue analysis showed that nitrogen levels were highest in the Hass variety, followed by Fuerte and Ettinger. At the Mojo site, nitrogen levels in Hass leaf samples declined from February to July, while remaining consistent at the MARC site. Pearson correlation analysis revealed synergistic relationships among manganese, copper, and calcium, as well as antagonistic interactions between nitrogen and zinc and competition between zinc and iron. These findings underscore the importance of optimizing nutrient management to promote healthy avocado growth and productivity. Practical implications include the need for targeted interventions to address deficiencies in zinc and iron in avocado soils, coupled with continuous leaf nutrient monitoring to align fertilization strategies with seasonal and varietal nutrient dynamics. Future studies correlating nutrient data with fruit yield, quality, and shelf life will provide valuable insights for maximizing avocado productivity and sustainability.

Keywords*: Avocado leaf analysis; nutrient dynamics; soil fertility; MARC and Moj; sampling months*

1. Introduction

Avocado shows great potential across various agroecological zones and can significantly benefit communities throughout some parts of Ethiopia by thriving in both domestic and international markets. Specifically, avocado cultivation can enhance farmers' incomes, optimize land use, promote sustainability, and bolster food security, as well as stimulate commercial and economic activities in growing regions. Avocados are rich in essential nutrients [8], are highly productive, and are recommended for combating malnutrition in impoverished countries like Ethiopia. The avocado tree, which can grow to a height of 10 to 12 meters and bear fruit year-round in its natural subtropical to tropical climates, is particularly prolific [15].

Hass, Fuerte, and Ettinger are key avocado cultivars in Ethiopia, valued for their adaptability, high yields, and contributions to smallholder farmers' livelihoods and the emerging avocado industry [41]. In Ethiopia, those avocado varieties are highly valued for their adaptability, quality, and contributions to food security, farmer incomes, and the country's growing role in the global avocado trade [42]. The Hass, Fuerte, and Ettinger avocados are prominent commercial varieties known for their high-quality fruit, with Hass offering a long fruiting season, Fuerte ensuring production consistency when paired with Hass, and Ettinger providing early-maturing, vigorous trees with excellent fruit quality [43].

The concentration of macronutrients (K, Ca, Mg, N, P, S) and micronutrients (Fe, Mn, Cu, Zn, B, Mo) varies at different development stages of the tree, such as flowering, fruit development, fruit maturity, and post-harvest, depending on the orchard plants [5]. Avocado leaf analysis is crucial for determining the optimal nutrient requirements for the fruit during different seasons to maximize production. Good soil is essential for establishing a healthy avocado tree [7], as understanding the most effective types of soil, fertilizers, agronomic practices, irrigation schedules, and mulching frequency is vital for the success of these trees.

Through photosynthesis, avocado leaves convert carbon dioxide and water into carbohydrates, transforming light energy into chemical energy necessary for plant growth [4, 29]. Calcium is vital for managing *Phytophthora* and fruit rots in avocados, while magnesium is needed for chlorophyll production, essential for photosynthesis. Sulfur plays a key role in protein synthesis, and boron is crucial for fruit development and setting [7, 12, 17]. Nitrogen is indispensable for growth, as it contributes to the formation of amino acids, proteins, and chlorophyll, all of which are necessary for photosynthesis and carbohydrate production [16]. Additionally, micro-elements such as Fe, Mn, Zn, Cu, and Mo are required for normal plant development and function [2, 21].

Analyzing the macro- and micronutrient content in avocado leaf samples offers several key advantages: including optimal fertilizer application, improved nutrient management, sustainable farming practices, enhanced fruit quality, disease and stress management, and increased profitability [23, 29], optimizing tree health, improving

fruit yield and quality, promoting sustainable practices, and ultimately increasing the profitability of avocado farming.

The avocado tree requires an optimal balance of nutrients for maximum fruit production, and deficiencies in nitrogen, potassium, and zinc can negatively impact fruit yield [4, 6, 29]. Analyzing the seasonal nutrient levels in avocado trees is essential for determining the appropriate timing for fertilizer application. This study aims to assess the nutrient status of avocado leaves during different seasons. Consequently, this research was initiated to evaluate the seasonal variation of nutrients in avocado orchards, aiming to supplement nutrients for sustainable nutrient management and production in the study areas.

2. Materials and Methods

2.1. Sampling site

The avocado leaf samples were collected from two locations: Mojo commercial avocado farms, specifically for the Hass variety, and the Melkassa Agricultural Research Center (MARC), which included samples from the Hass, Fuerte, and Ettinger varieties. The avocado trees at MARC were planted in 2001, arranged in a 6 m by 6 m grid pattern [45]. The orchard soil is loamy, and the trees are irrigated using furrow irrigation. All trees at MARC are managed under uniform agronomic practices. Similarly, the Hass avocado trees at the Mojo site were established in 2021 and follow comparable management practices to those at MARC. Soil samples were also collected from the Mojo commercial avocado farms. The Mojo farm is located in Lome Woreda, East Shewa Zone, Oromia region, with geographic coordinates of 8°39′N latitude and 39°5′E longitude, at an elevation ranging between 1,788 and 1,825 meters above sea level. Melkassa Agricultural Research Center (MARC) avocado experimental site is located at the Center station. The Center is geographically located at a latitude of 8˚24’N, longitude of 39˚21’ E, and an altitude of 1,550 meters above sea level. It is situated about 107 km from Addis Ababa and 17 km from Adama on the way to Assela town of the Oromia Region.

2.2. Soil sampling and analysis

Soil samples were taken from the three farmer’s fields. Samples were taken in two depths 0-20 cm and 20-50 cm and samples were prepared and analyzed for pH, electrical conductivity (EC), organic carbon (OC), total nitrogen (TN), cation exchange capacity (CEC), iron (Fe), cupper (Cu), Zinc (Zn), manganese (Mn) following standard procedure [11, 28, 33].

2.3. Sampling of avocado leaves

Commercial avocado farms in Lomé Wereda were selected in mid-January through random selection and observation by horticultural and soil fertility researchers from the MARC team. The trees were chosen based on their flowering stage, as well as the specific area and landscape of the farm. For each landscape, a representative

tree was identified to ensure accurate sampling. Furthermore, the timing of sample collection (flushing stage) was carefully determined to align with the physiological condition of the trees, providing meaningful and consistent data for the study. None shaded branches from the middle part of the canopy and fully expanded, healthy, neither too young nor too old leaves which are 4-7 months old were choosen [37]. The commercial Hass avocado variety was selected and sampled due to it's the common commercial avocado variety in the area. Similarly, the sample from the three varieties of avocado trees (Hass, Fuerte and Ettinger) at MARC were selected on the same Month. After selecting the sampling sites, random avocado trees were selected and tagged for leaf sampling. Three Hass variety avocado tree from each farmer's field which a total of nine trees were tagged at Mojo site and 9 trees from each variety (Hass, Fuerte and Ettinger), a total of 27 trees were selected and tagged at MARC site. From the three farmers field at Mojo site, nine trees were selected starting from the 1st February, a continuous leaf sampling was done at each Month until July. Samples were collected following standard procedures [25, 33] from the tagged sampled avocado trees and transported to MARC Soil, Plant and Water Laboratory for sample processing and analysis of N, Fe, Mn, Cu, Zn and Ca.

2.4. Leaf sample preparation and analysis

After the collection of leaves from the selected trees, samples were air dried and grinded using an electrical blending device, and then the powder was sieved through a 0.5 mm sieve to prepare the samples for the laboratory analysis. 0.5 g of grounded and homogenized powder samples were weighed and transferred to acid-washed porcelain crucibles and transferred to a muffle furnace and the dry ashing method was carried out at 500 ^{0}C for 5 hours following the standard procedure as described by Estefan [11] and Wogi [33].

2.5. Apparatus and equipment

Benchtop pH meter and electrical conductivity meter were used to measure pH and electrical conductivity (EC) respectively. Fully automated Flame Atomic Absorption Spectrophotometer (FAAS) (Agilent 200 Series AA, USA) with fast sequential operation for multi-element analysis was used to determine micronutrients (Cu, Mn, Fe, Zn) in soil and avocado leaf sample. Kjeldahl digestion and distillation unit and digital burette were used to determine the total Kjeldahl nitrogen level of soil and avocado leaf sample. Digital furnace (Nabertherm, Germany) was used to ash the leaf samples for the extraction of minerals.

2.6. Reagents and standards

Soil microelements (Cu, Mn, Fe, and Zn) were extracted by di-ethylene tri-amine penta-acetic acid (DTPA). All the reagents and chemicals used in the present study were analytical grade. 500 ppm stock solutions of certified reference materials (Agilent Technologies, USA) for microelements (Cu, Mn, Fe, Zn) were used for the preparation of series of standards. 2 N HCl (Aldrich, A.C.S. Reagent, Germany) was used to dissolve the ash

sample of avocado leaves. Deionized water and DTPA reagent [a mixture of 0.005 M DTPA, 0.1 M triethanolamine (TEA), 0.1 M $CaCl_2$] were used for all the preparation and dilution purposes to prepare standard series of microelements of soil sample analysis. 5 % HNO_3 was used to prepare a serious of standards for leaf micronutrient analysis.

2.7. Calibration

Intermediate standard solution of 50 ppm was prepared from a standard reference stock solution of 500 ppm Agilent Certified Reference Material for each metal by diluting 10 mL of standard reference metal with 5 % HNO_3 to a 100 mL solution. From the intermediate stock solution (50 ppm), five working standard solutions were prepared for the calibration curve of each element. A series of 0, 2, 5, 7, 9 ppm standard solutions were prepared for Ca calibration, and a series of 0, 2, 4, 6, 8, 10 ppm micronutrient (Fe, Cu, Mn, Zn) standards were prepared for the calibration of micronutrients (Fe, Cu, Mn, Zn) for each batch analysis of both soil and avocado leaf analysis.

2.8. Quality assurance and quality control

Table 1: Standard conditions of the AAS (Agilent 200 series AA) used for nutrient determination for each analyte.

Metals	Wavelength	Lamp current	Slit width	Optimum working range
	nm	mA	nm	µg/mL
Mn	279.5	5	0.2	0.02–50
Cu	324.7	4	0.5	0.03–10
Zn	213.9	5	1.0	0.01–20
Fe	248.3	5	0.1	0.06–15
Ca	422.7	10	0.5	0.01–3.00

2.9. Recovery analysis of soil sample

The influence of matrix components was evaluated by determination of recovery after spike of the leaf and soil sample extract with standards of metals to be determined .

$$\%Recovery = \frac{C_s - C_{unsp}}{C_{added}} \times 100 \qquad (1)$$

Where, C_s = concentration of spiked sample, C_{unsp} = concentration of un-spiked sample and C_{added} = added concentration.

20 mL of soil and leaf sample extract was spiked with 2 mL of 8 ppm micronutrient (Cu, Mn, Fe, Zn) standard solution for the recovery analysis of both soil and leaf sample. 20 mL of leaf sample extract was spiked with 2 mL of 50 ppm Ca standard solution to analyze the recovery of leaf sample Ca analysis.

2.10. Limit of detection (LOD) and limit of quantification (LOQ)

$$LOD = \frac{3 \times \delta}{S} \quad (2)$$

$$LOQ = \frac{10 \times \delta}{S} \quad (3)$$

Where, LOD is limit of detection, LOQ is limit of quantification, δ is standard deviation and S is slope of the calibration curve equation.

Table 2: Concentration of intermediate standard, concentration of working standards, correlation coefficient of calibration curve, limit of detection, limit of quantification and recovery analysis of soil sample.

Metals	CIS	CWS	CCCC	LOD	LOQ	Recovery
		ppm				%
Mn	50	0, 0.4, 0.8, 1.2, 1.6, 2.0	0.9996	0.055	0.168	96.48
Cu	50	0, 0.4, 0.8, 1.2, 1.6, 2.0	0.9999	0.026	0.078	96.93
Zn	50	0, 0.4, 0.8, 1.2, 1.6, 2.0	0.9999	0.027	0.081	95.90
Fe	50	0, 0.4, 0.8, 1.2, 1.6, 2.0	0.9998	0.040	0.122	----------

NB: CIS = concentration of intermediate standard, CWS = concentration of working standards, CCCC = correlation coefficient of calibration curve, LOD = limit of detection, LOQ = limit of quantification

Table 3: Concentration of intermediate standard, concentration of working standards, correlation coefficient of calibration curve, limit of detection, limit of quantification and recovery analysis of leaf sample.

Metals	CIS	CWS	CCCC	LOD	LOQ	Recovery
		ppm				%
Mn	50	0, 2, 4, 6, 8, 10	0.9999	0.132	0.399	104.07
Cu	50	0, 2, 4, 6, 8, 10	0.9999	0.002	0.005	95.80
Zn	50	0, 2, 4, 6, 8, 10	0.9999	0.220	0.667	96.19
Fe	50	0, 2, 4, 6, 8, 10	0.9997	0.221	0.668	102.42
Ca	50	0, 2, 5, 7, 9	0.9998	0.210	0.636	95.49

2.11. Data analysis

The results of soil and leaf analysis were subjected to R software to analyze the data, to calculate Pearson correlation coefficient, and to draw graphs.

3. Results

3.1. Soil nutrient status at Mojo site

The analysis results revealed that the soil has a slightly alkaline pH (7.49–7.78), which does not impact avocado production. The soil provides adequate N, P, and K availability; however, its pH somewhat limits the accessibility of micro nutrients such as Fe, Zn, Cu, and B. The cation exchange capacity (CEC) values from the soil analysis were found in the range 30.7–39.8 mEq/100 g soil for all the selected fields showing that it provides excellent nutrient retention, and hence wider interval for fertilizer application. The level of soil nitrogen was found in the range between 0.05 – 0.12% very low across all sites (Table 4). The organic carbon (OC) result showed that one farmer's field (Field 1) had low OC levels (0.64% and 0.70% for 0–20 cm and 20–50 cm, respectively), while the other fields were classified as having moderate OC levels (1.1–1.74%). The EC value in the sampling area range (98.8 to 218.0 µS/cm) which is rated as a normal range. The micronutrient concentration of the analysis reviled that the copper content in the soil found to be in the medium range (0.62 – 0.90 ppm). Fe content in the soil was below detection limit. The manganese content ranges from medium to high (0.54 – 2.62 ppm), and the zinc levels range from very low to medium (0.09 – 0.42 ppm).

Table 4: Soil chemical properties of commercial avocado farms at Mojo.

**Parameters**									**Ratings**	**Reference**
**Farm**	1		2		3- Site 1		3 Site 2			
**Depth (cm)**	0-20	20-50	0-20	20-50	0-20	20-50	0-20	20-50		
**pH**	7.64	7.72	7.73	7.83	7.85	7.24	7.37	7.61	Neutral-Slightly alkaline	[30, 31]
**EC (µS/cm)**	132.2	117.9	178.2	218	98.8	142.9	132.7	188.1	Normal	[32, 33]
**Sand (%)**	25	22	31	29	24	25	15	15		
**Clay (%)**	31	38	27	33	32	33	49	49		
**Silt (%)**	44	40	38	44	44	42	36	36		
**Textural class**	CL	CL	L/CL	CL	CL	CL	C	C		
**CEC**	34.1	37.8	37.0	39.8	30.7	31.4	34.0	35.8	High	[39]
**TOC (%)**	0.70	0.64	1.56	1.62	1.74	1.10	1.56	1.57	Low-Moderate	[40]
**TN (%)**	0.06	0.05	0.10	0.09	0.12	0.08	0.09	0.09	Low	[39]
**C:N ratio**	11.7	12.8	15.6	18.0	14.5	13.75	17.33	17.44		
**Cu (ppm)**	0.90	0.79	0.90	0.73	0.74	0.66	0.72	0.62	Medium	
**Fe (ppm)**	ND	ND	ND	ND	ND	ND	ND	ND	Very low	
**Mn (ppm)**	2.08	0.54	1.66	0.88	2.49	1.54	2.62	0.78	Medium-High	[37]
**Zn (ppm)**	0.09	0.12	0.28	0.22	0.42	0.25	0.29	0.24	Very low-Medium	

EC = electrical conductivity, CEC = cation exchange capacity, TOC = total organic carbon, TN = total nitrogen, Cu = cupper, Fe = Iron, Mn = manganese, Zn = zinc, ppm= part per million, cm= centimeter, CL = clay loam, L/CL = loam/clay loam, C = clay

The results indicated that Mn consistently decrease with depth in all the farms while, Zn shows inconsistent trends, with levels slightly fluctuating between depths.

3.2. Avocado leaf nitrogen level over different sampling Months

The nitrogen levels in Hass avocado leaves varied across different months and locations. At Mojo, the highest nitrogen level (1.52%) was recorded in February, followed by a gradual decrease from February to June (Figure 4). In contrast, at MARC, the highest nitrogen level (1.48%) was recorded in May, with relatively consistent levels across the other months. The lowest nitrogen levels were recorded in May at Mojo (1.30%) (Figure 4) and in June at MARC (1.20%) (Figure 1). The mean nitrogen levels at Mojo ranged from 1.30% in May to 1.52% in February, while at MARC, the mean level ranged from 1.20% in June to 1.48% in May. Overall, the mean analysis of nitrogen levels were generally lower at MARC compared to Mojo (Figure 5).

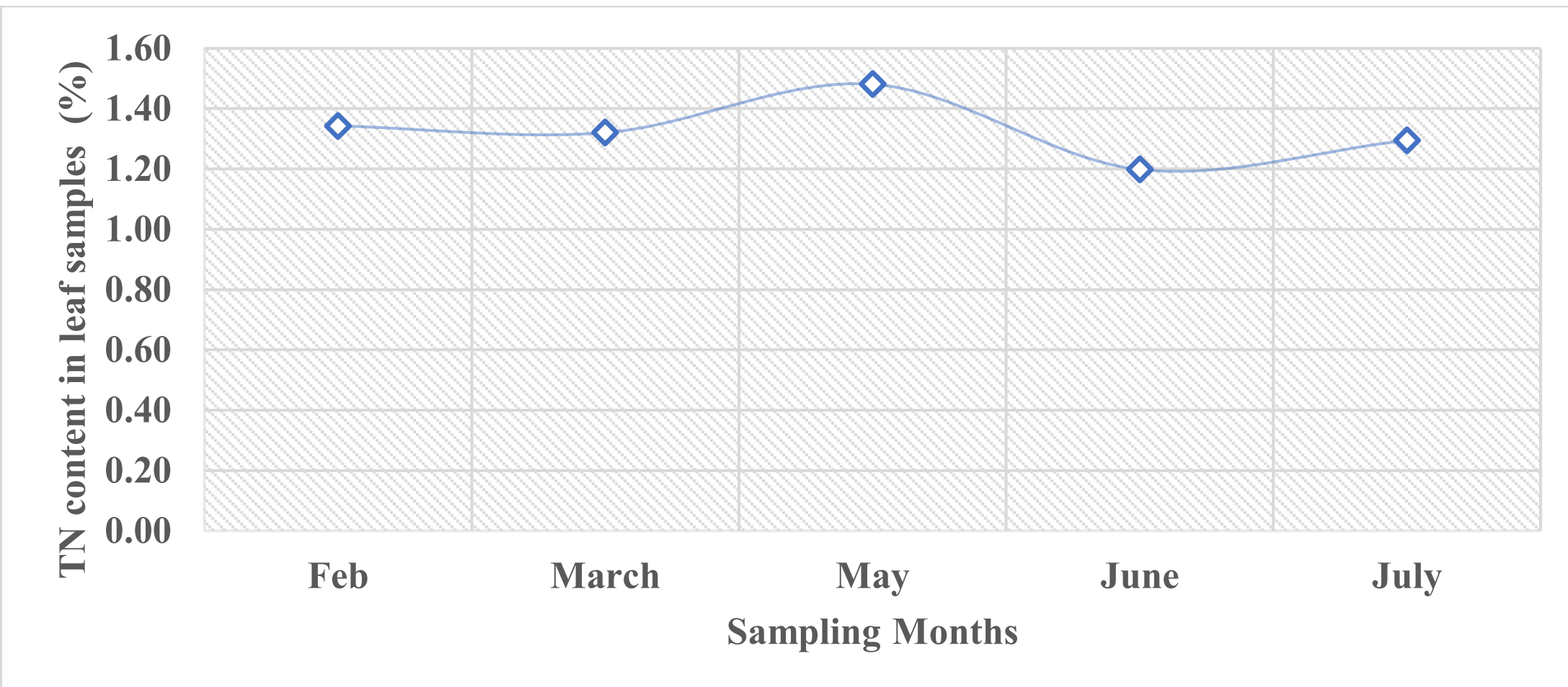

Figure 1: Leaf total nitrogen concentration of Hass avocado variety over different sampling months at MARC.

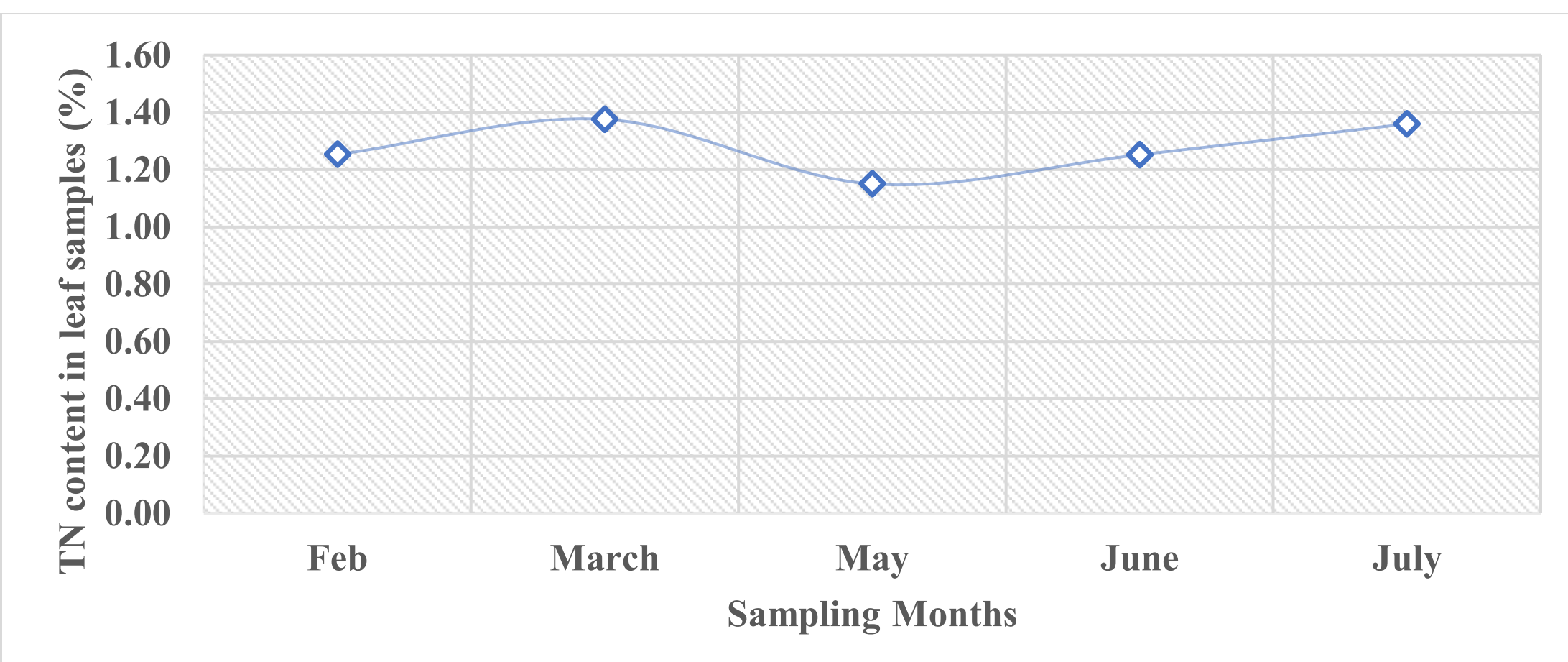

Figure 2: Leaf total nitrogen concentration of Fuerte avocado variety over different sampling months at MARC.

The TN level for the Fuerte variety is comparatively lower than that of the Hass variety over the sampling Months. TN analysis of leaf samples from the three varieties (Hass, Fuerte, and Ettinger) reveals that the Hass variety has the highest nitrogen level, followed by Fuerte and Ettinger. The mean TN values of leaf samples across different

sampling seasons were 1.32, 1.27, and 1.13 for the Hass, Fuerte, and Ettinger varieties, respectively. These findings are consistent with the literature reported by Bender and Faber [4]. Analysis of the Ettinger variety leaf samples shows fluctuations in nitrogen content across different sampling months. However, the nitrogen content in all the selected avocado tree leaf samples of the Ettinger variety is more consistent compared to the other varieties of selected trees (Figure 3).

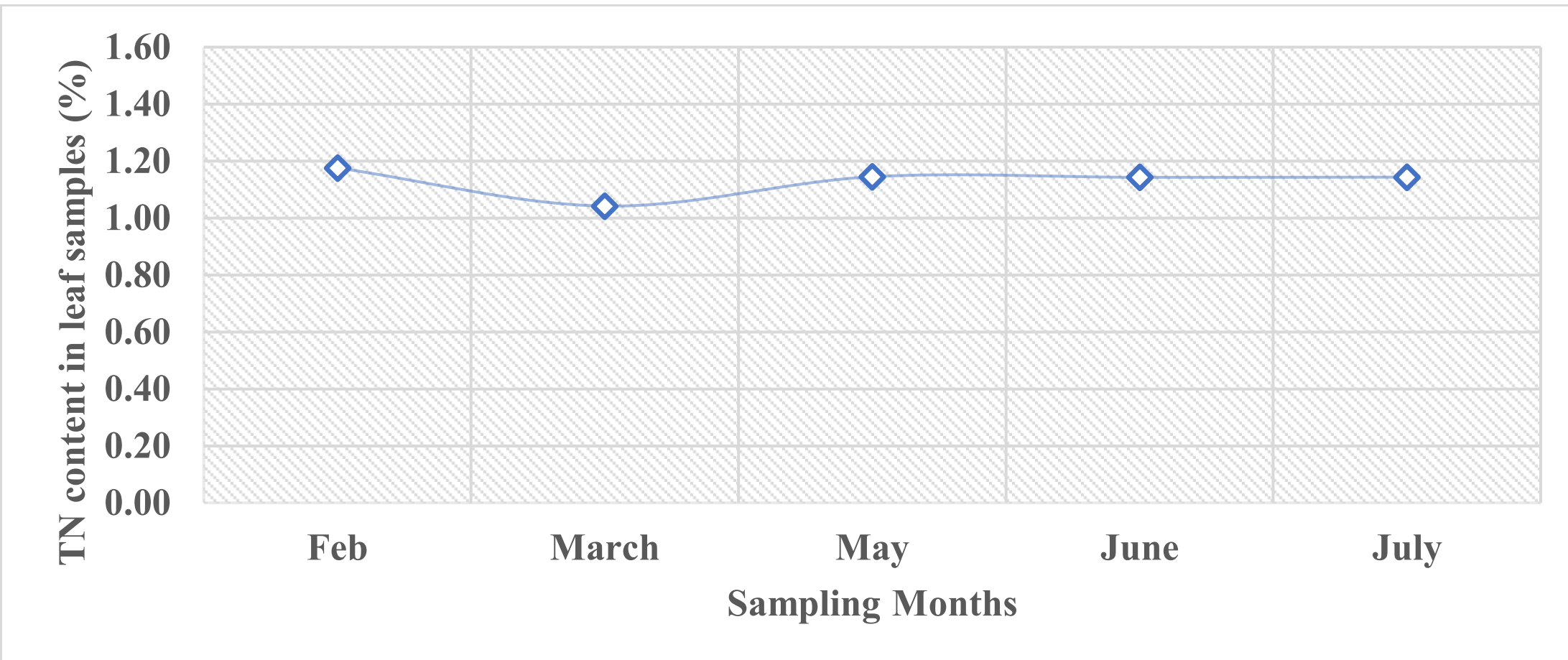

Figure 3: Leaf total nitrogen concentration of Ettinger avocado variety over different sampling months at MARC.

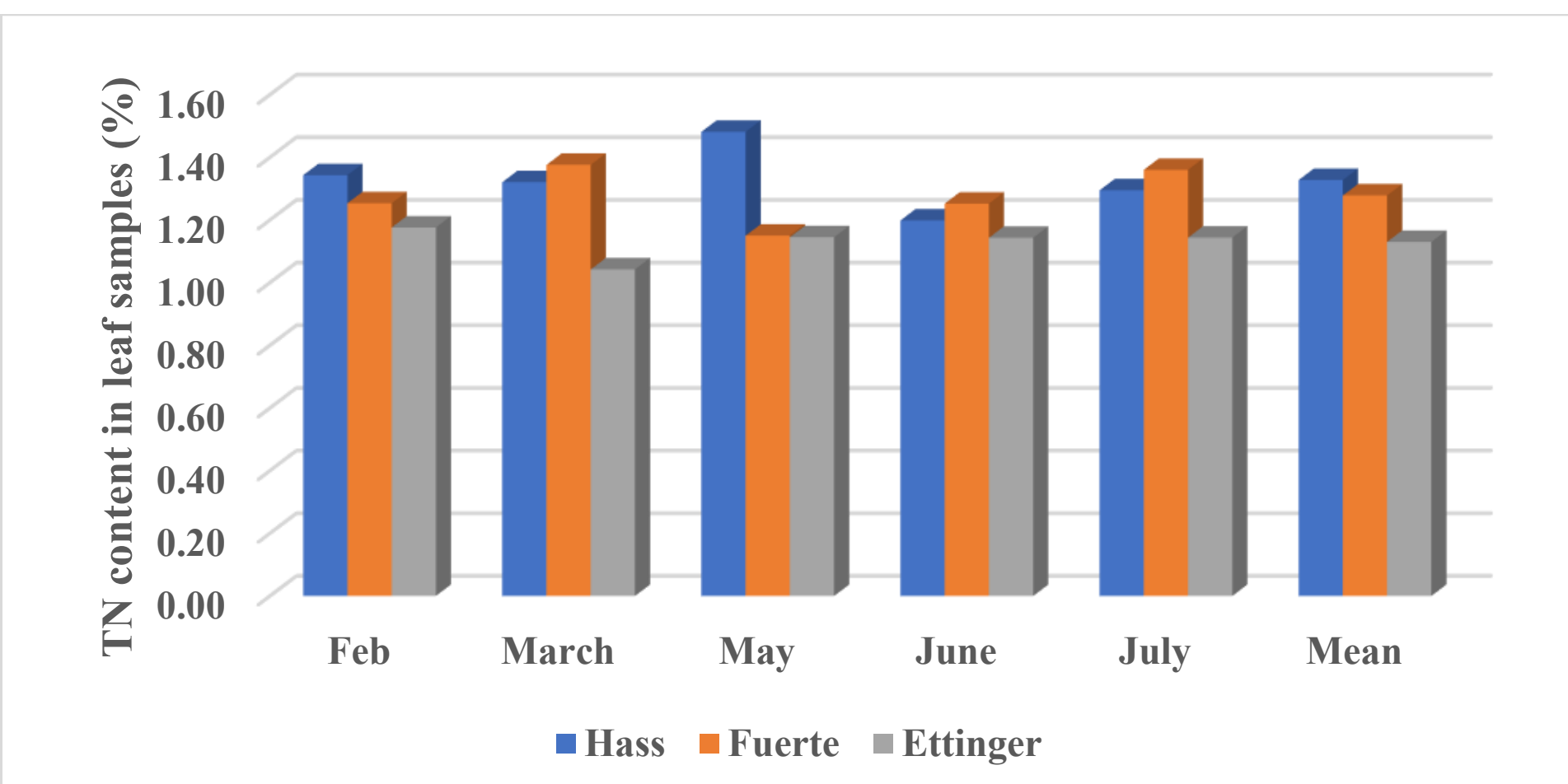

Figure 4: Leaf total nitrogen concentration dynamic in avocado varieties over different sampling months at MARC.

Figure 4 shows that Hass variety consistantly shows higher mean N content (1.33) compared to Fuerte (1.28) and (Ettinger (1.13), suggesting that it accumulates more N in its leaves for fruit growth. The highest nutrient content in Hass avocado was occured in May (1.48), suggesting peak nitrogen accumulation during this peored, potentially corresponding to critical growth stages. Furite displays its highest nutrient content in March (1.38),

while Ettinger demonstrates consistently low and stable nutrient levels, minimal fluctuation around its mean. This stability may indicate less dynamic nutrient uptake or utilization compared to the other varieties.

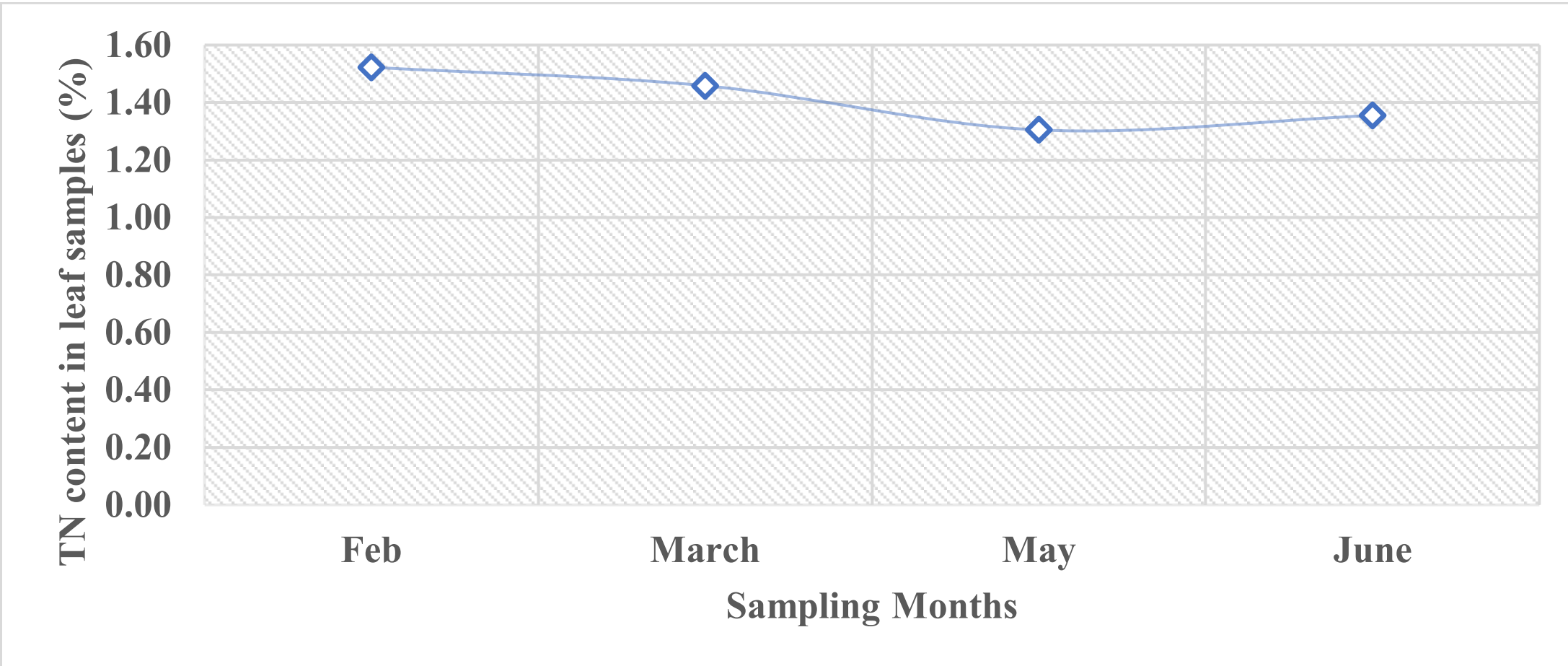

Figure 5: Mean TN concentration in Hass avocado variety leaf samples at Mojo over different sampling months.

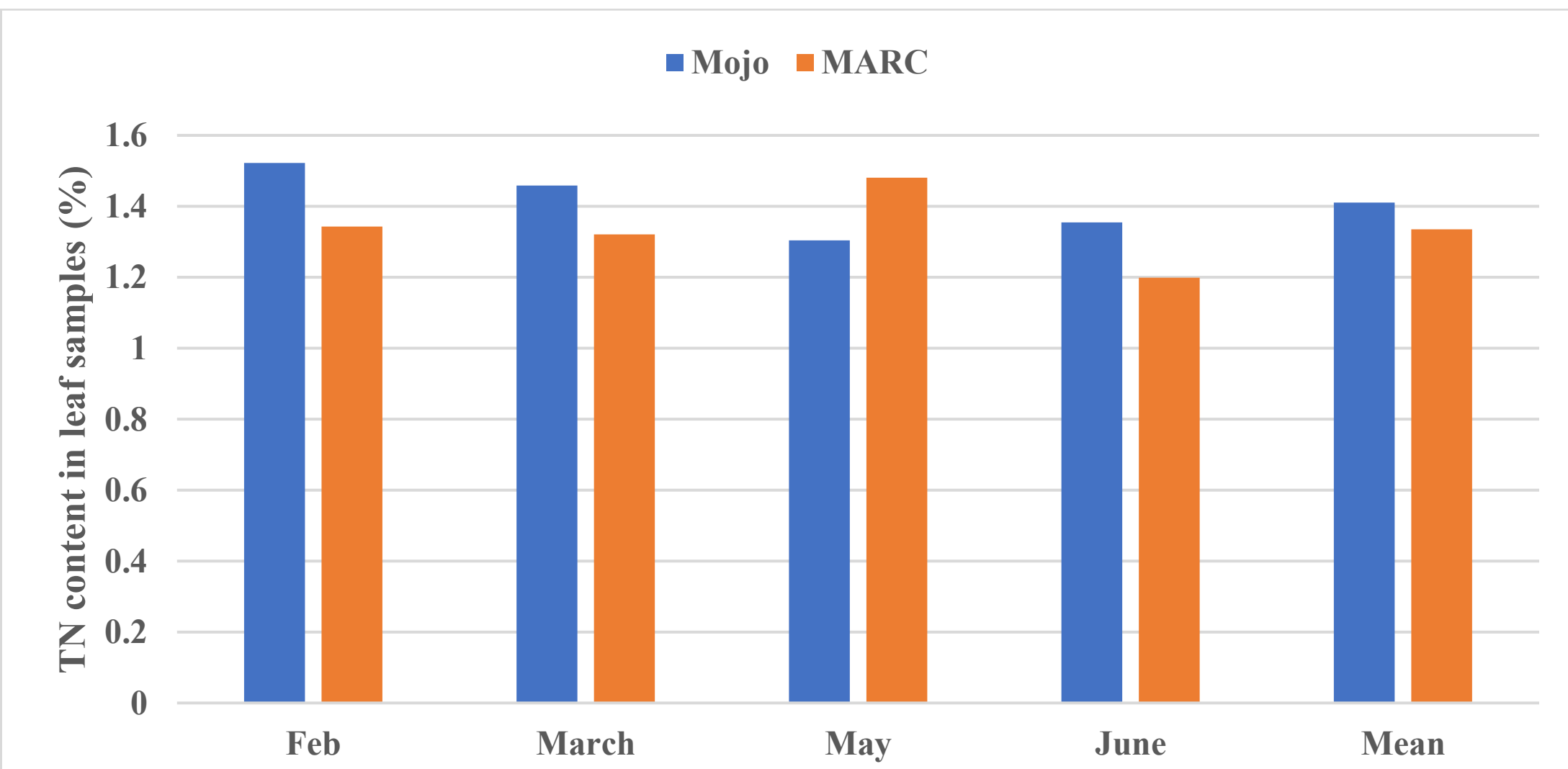

Figure 6: Concentration of TN in Hass avocado leaf at Mojo and MARC site at different sampling Month.

3.3. Mean level of micronutrients in avocado varieties leaf sample at different sampling Months

The overall mean value for the analysis of micronutrients (Fe, Cu, Zn, Mn) in leaf samples from the three avocado varieties across different sampling months at the MARC site shows that the Ettinger variety has higher micronutrient concentrations (Figure 7). The leaf analysis results reveal that the Fuerte and Hass varieties have comparable levels of micronutrients. The highest level of zinc (Zn) was recorded in the Ettinger variety in June, and in the Fuerte variety in May. Elevated levels of copper (Cu) and iron (Fe) were observed in July compared to the other sampling months.

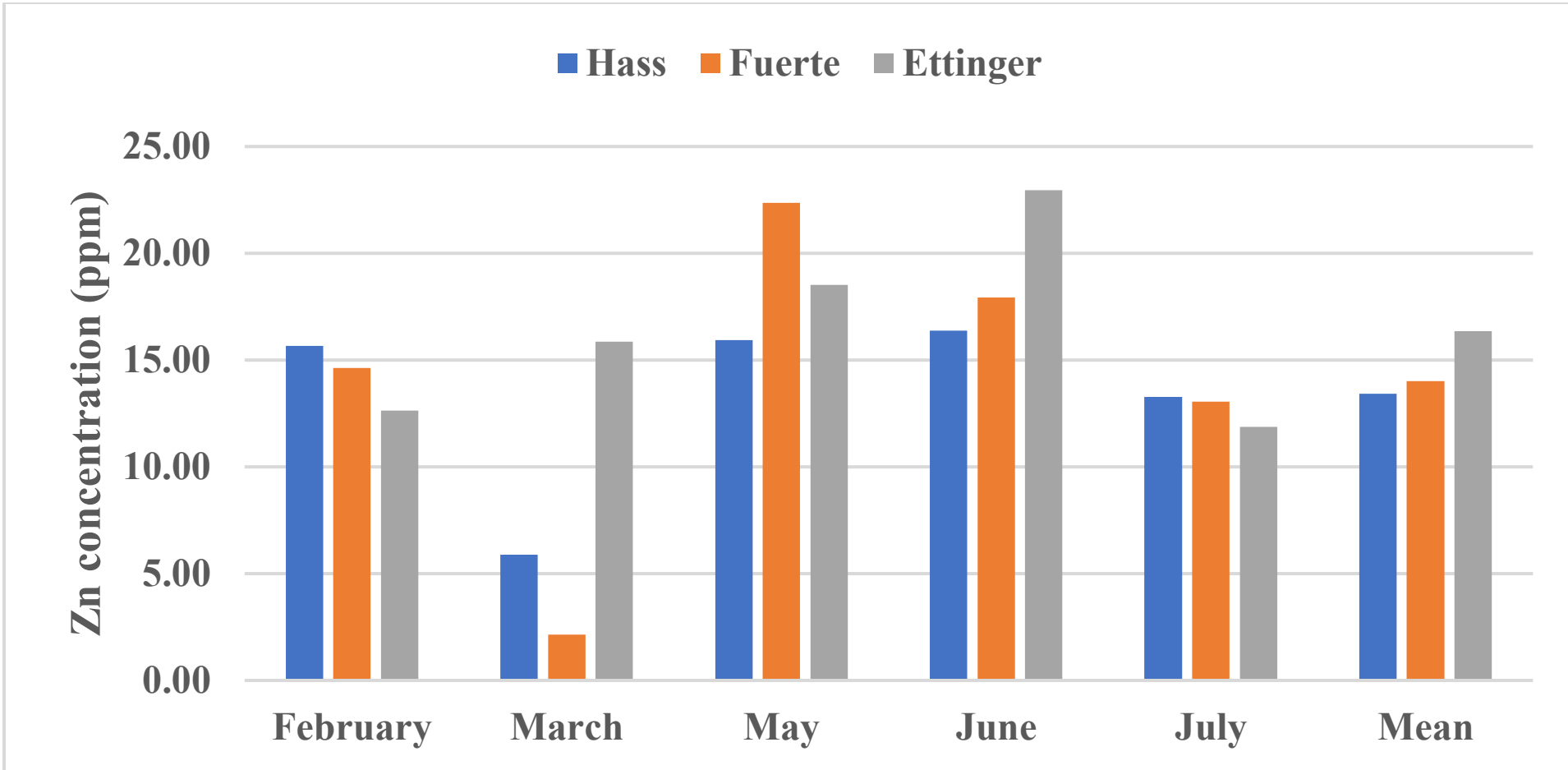
Hass Fuerte Ettinger
Zn concentration (ppm)
25.00
20.00
15.00
10.00
5.00
0.00
February March May June July Mean
(a)

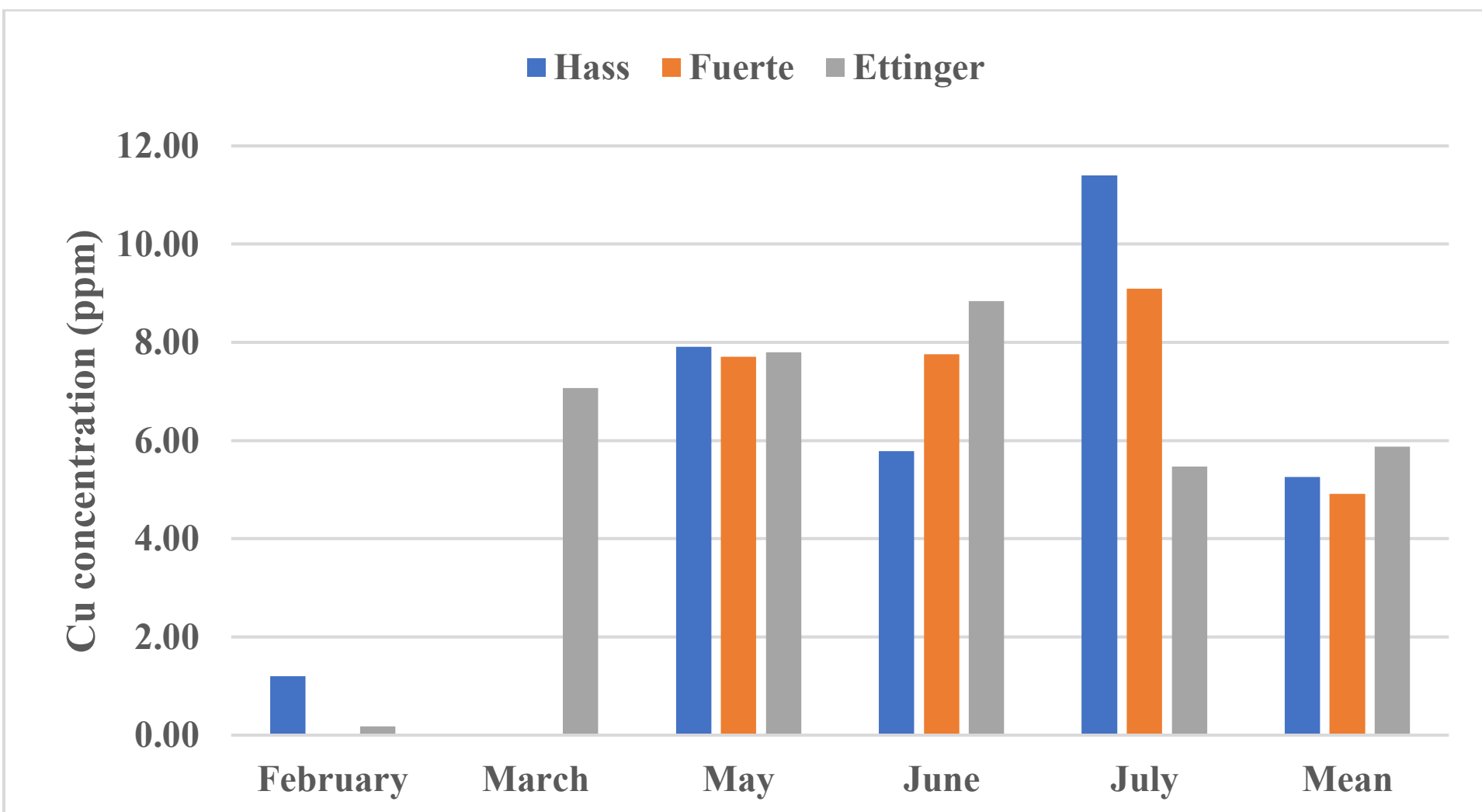
Hass Fuerte Ettinger
Cu concentration (ppm)
12.00
10.00
8.00
6.00
4.00
2.00
0.00
February March May June July Mean
(b)

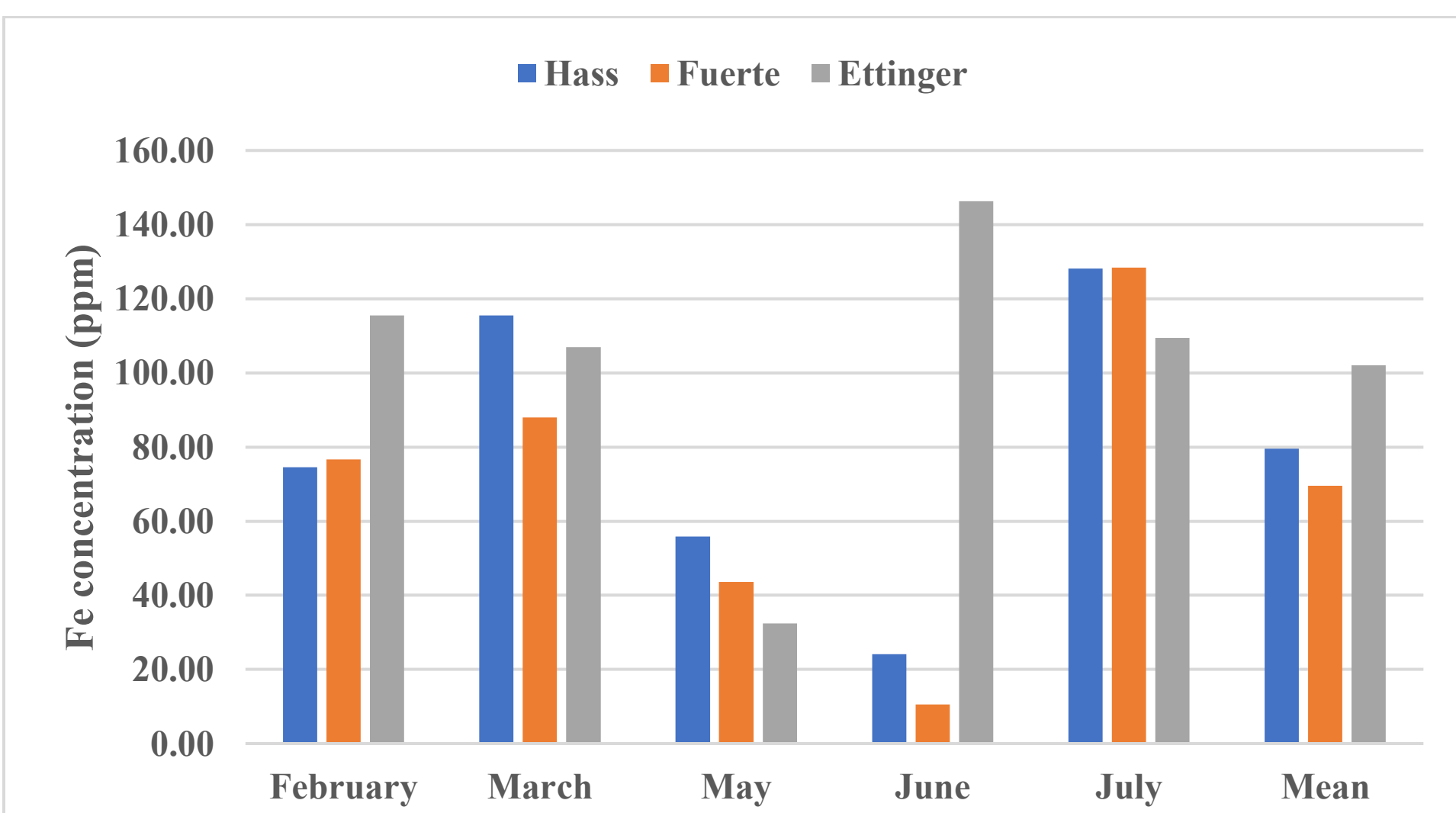
Hass Fuerte Ettinger
Fe concentration (ppm)
160.00
140.00
120.00
100.00
80.00
60.00
40.00
20.00
0.00
February March May June July Mean
(c)

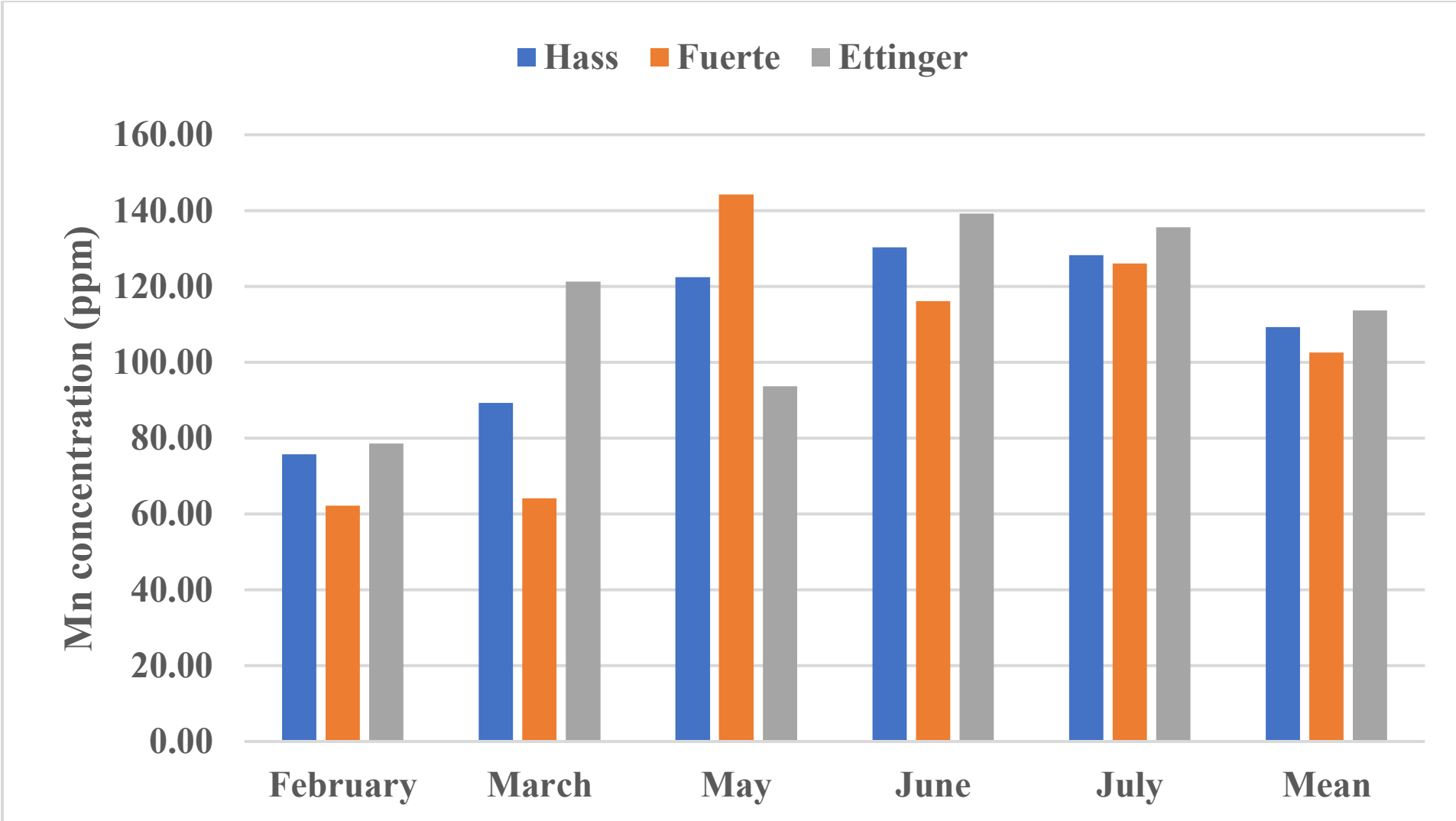

(d)

Figure 7: Mean dry-weight micronutrient concentration (a = Zn, b = Cu, c = Fe, and d = Mn in ppm) over different sampling months for three avocado varieties at MARC.

Table 5: Concentration of macro and micro nutrients in avocado leaves from different sources as compared to the present study.

Macro and micro elements	Measurement unit	Optimum range	References	Concentration of leaf nutrient in present study		
				Hass	Fuerte	Ettinger
N	%	1.6-2.2	[4, 18, 19, 23, 24, 27]	1.32-1.41	1.06-1.65	0.88-1.29
Ca	%	1-3	[4, 18, 19, 23, 24, 27]	1.19-1.33	0.79-1.61	1.00-1.61
Fe	ppm	50-200	[18, 23]	71.48-79.62	10.62-128.43	32.44-146.34
Mn	ppm	30-500	[18, 23]	109.23-146.61	62.13-144.29	78.58-139.16
Cu	ppm	NA	[18, 26, 27]	5.26-7.67	0.00-9.09	0.18-8.84
Zn	ppm	NA	[18, 23, 27]	13.43-16.04	2.14-22.37	11.86-22.96

The levels of all micronutrients were minimal in February and March. Among the micronutrients, Cu and Fe concentrations during these months were extremely low compared to Zn and manganese (Mn). For the Hass variety, Mn levels showed a consistent increase from February to July. The highest level of Mn in the Fuerte variety was recorded in May.

3.4. Level of micronutrients in different avocado varieties and locations at different sampling Months

The concentration of micronutrients in avocado leaves varied significantly across different varieties, locations, and sampling months. Below, we present the observed trends for each micronutrient (Mn, Zn, Cu, and Fe) in Hass, Fuerte, and Ettinger avocado varieties at the Mojo and MARC sites. Mn concentrations in Hass avocado leaves showed a general increase from February to July, with the highest values observed in May. At the Mojo site, the mean Mn concentration was 146.61 ppm, significantly higher than at the MARC site, where the mean was 109.23 ppm. In Fuerte avocado leaves, Mn concentrations increased from February to July, with the highest mean value observed at MARC (102.56 ppm). Similarly, Ettinger avocado leaves showed a steady increase in Mn, reaching

a mean of 113.63 ppm at MARC. Zn levels exhibited variability across the sampling months, with notable increases in May and June. The mean Zn concentration at Mojo was 16.04 ppm, compared to 13.43 ppm at MARC. Zn levels in Fuerte avocado leaves were relatively stable, with a mean of 14.03 ppm at MARC. In contrast, Ettinger avocado leaves had the highest mean Zn concentration (16.36 ppm) among all varieties. Cu levels were initially low or absent in February but increased steadily from May onwards. The highest mean Cu concentration was observed at Mojo (7.67 ppm), while at MARC, it was 5.26 ppm. Cu levels in Fuerte avocado leaves increased over time, with a mean of 4.91 ppm at MARC. Ettinger avocado leaves showed a similar trend, with a mean Cu concentration of 5.87 ppm. Fe concentrations fluctuated significantly across the sampling months, with peaks typically observed in July. Fe levels were higher at MARC (79.62 ppm) compared to Mojo (71.48 ppm). Fe concentrations in Fuerte avocado leaves were relatively low, with a mean of 69.49 ppm at MARC. In contrast, Ettinger avocado leaves had the highest mean Fe concentration (102.12 ppm).

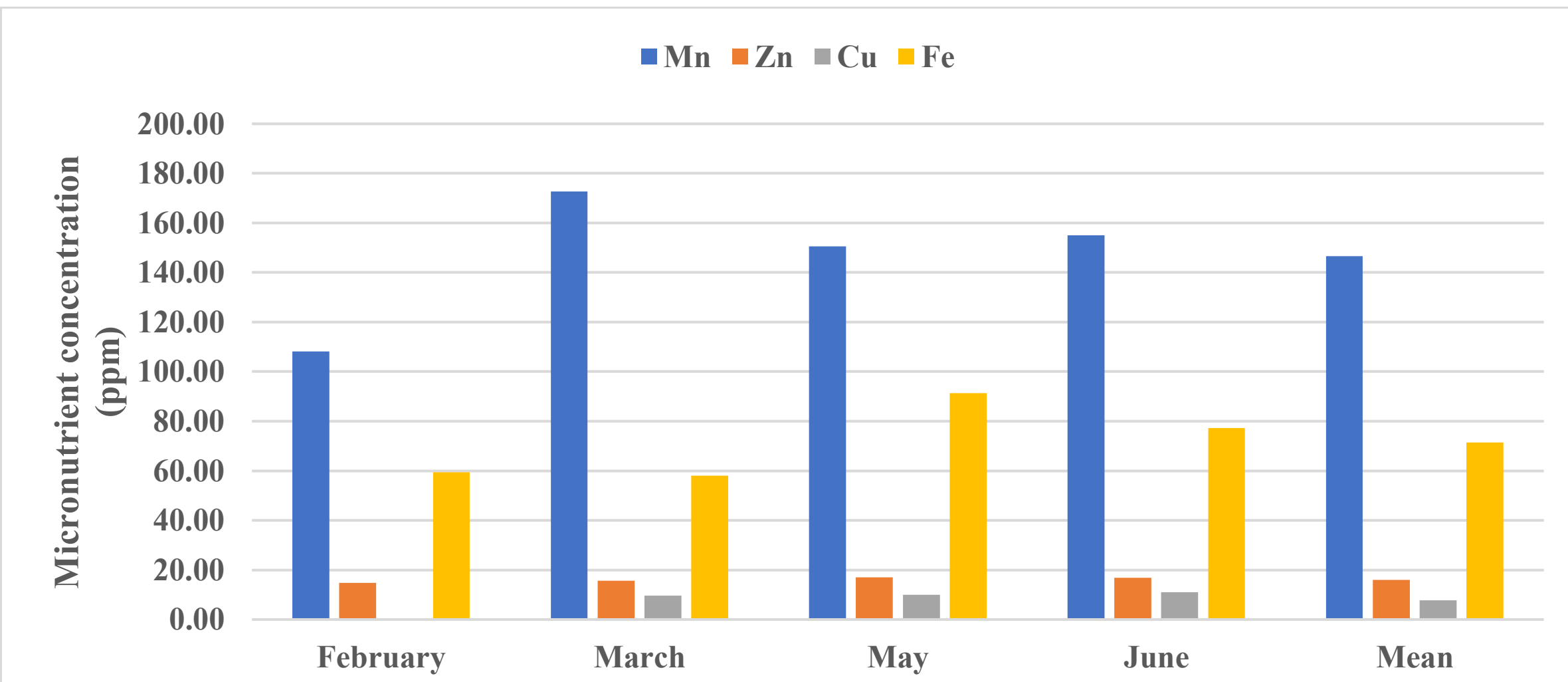

Figure 8: Micronutrient concentration in Hass avocado leaves over different sampling Months at Mojo site.

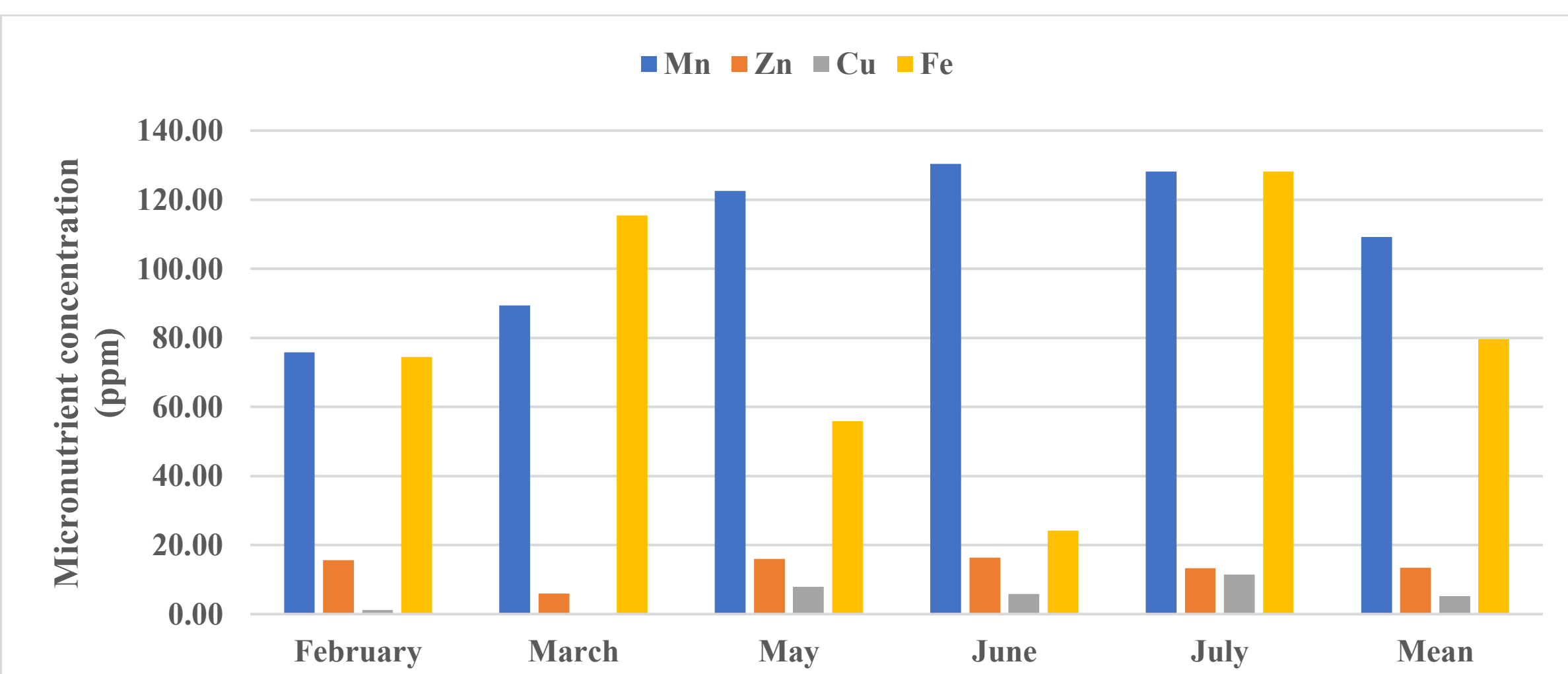

Figure 9: Micronutrient concentration in Hass avocado leaves over different sampling Months at MARC site.

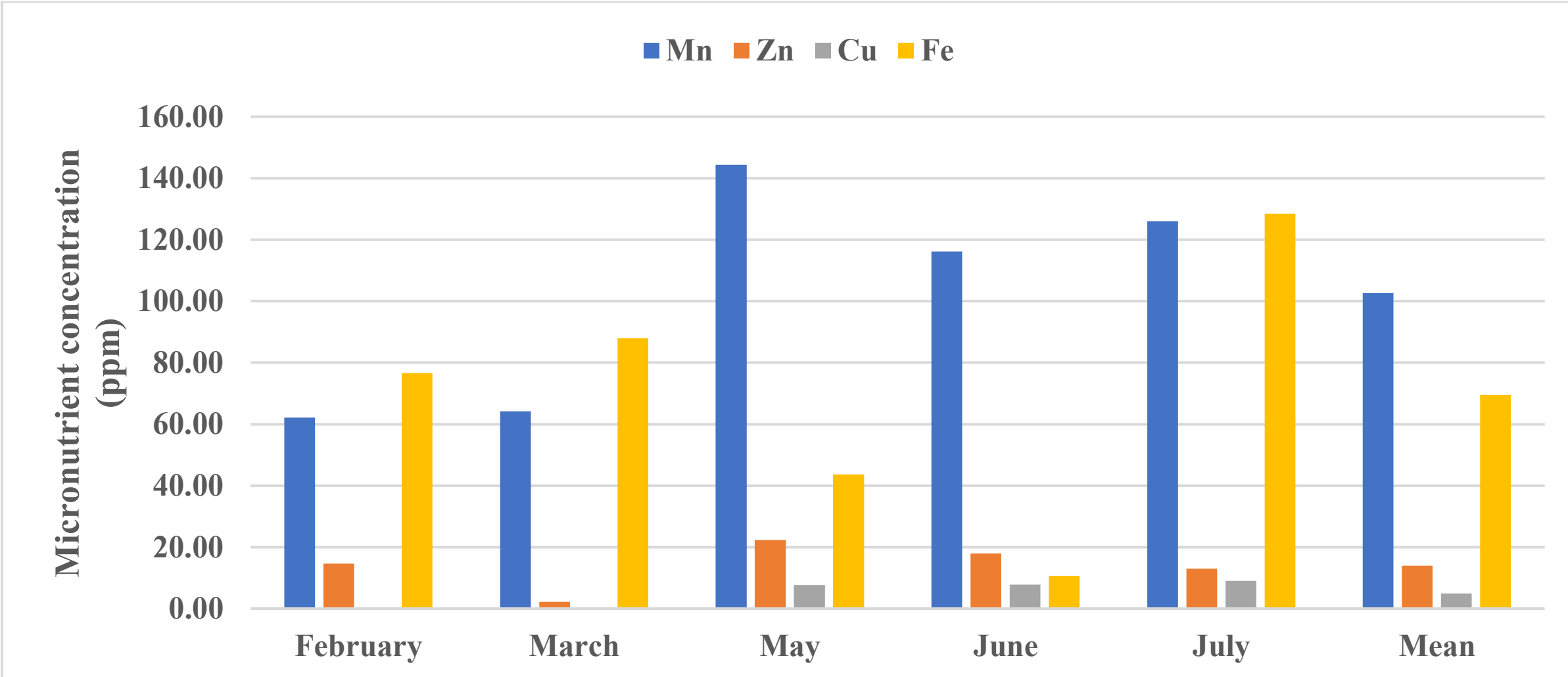

Figure 10: Micronutrient concentration in Fuerte avocado leaves over different sampling Months at MARC site.

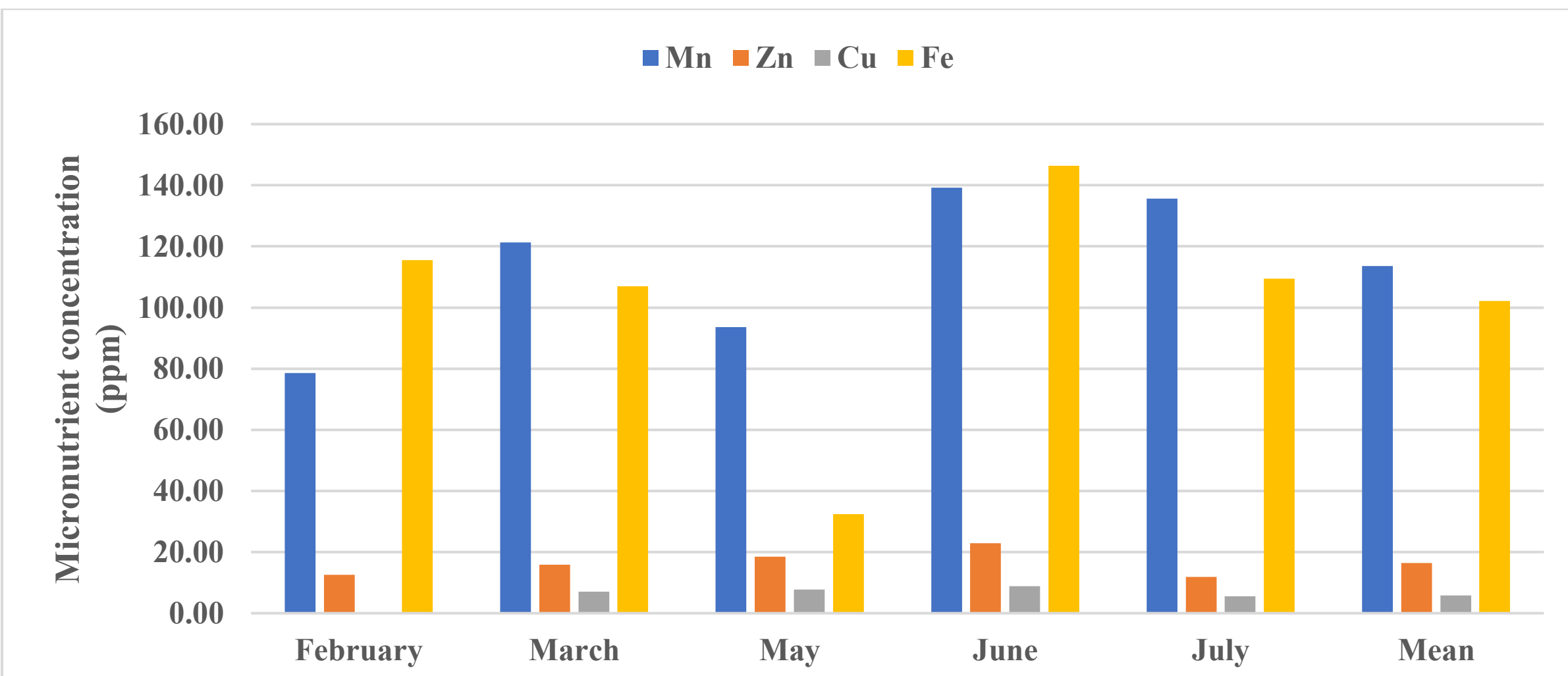

Figure 11: Micronutrient concentration in Ettinger avocado leaves over different sampling Months at MARC site.

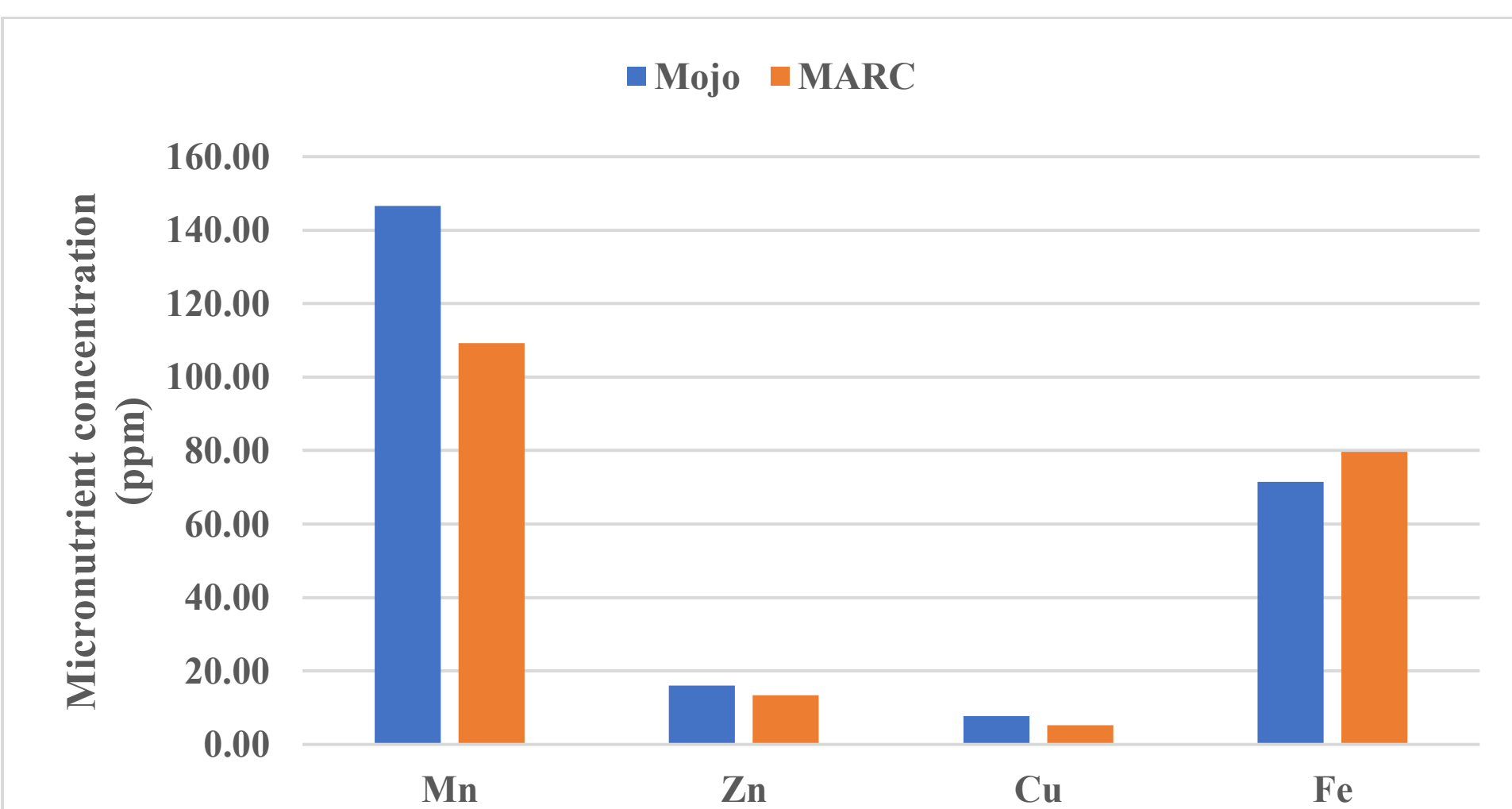

Figure 12: Micronutrient concentration in Hass avocado avocado leaves at the two locations (Mojo and MARC).

3.5. Level of Ca concentration in different avocado varieties and locations at different sampling season

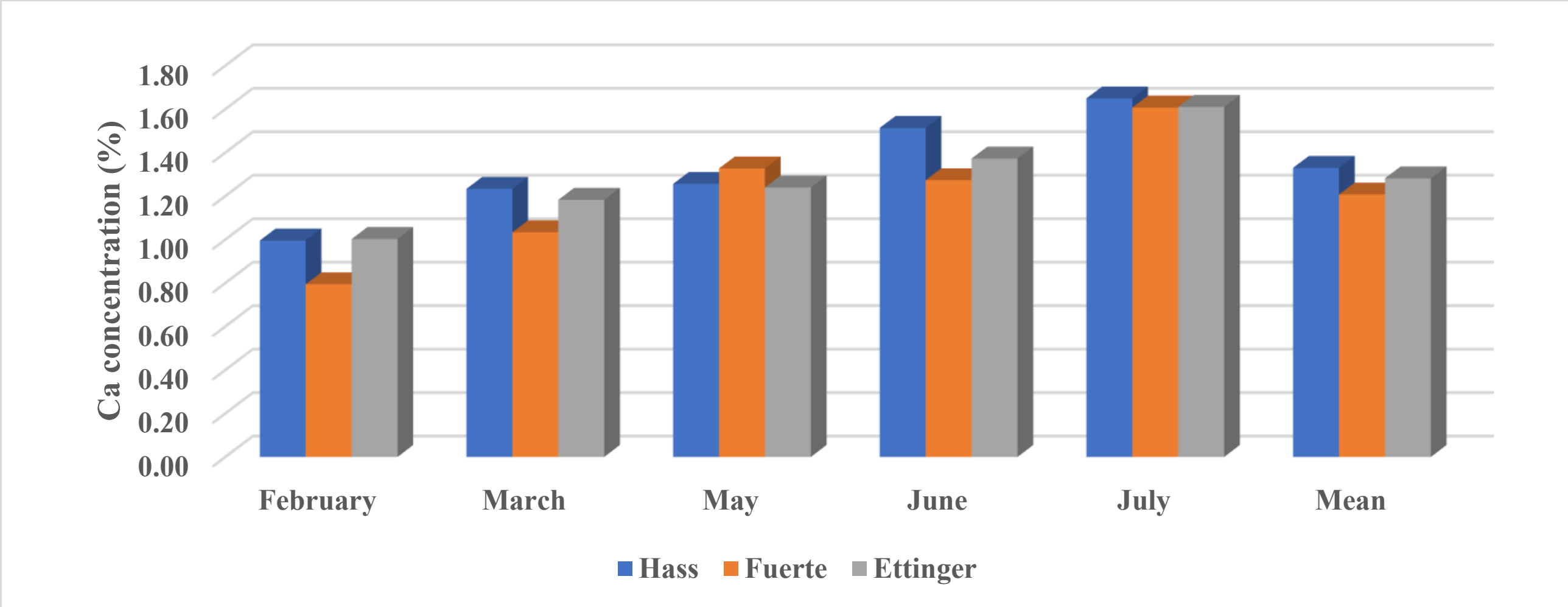

Figure 13: Mean Ca concentration of leaf at different sampling Months of the three-avocado variety at MARC.

The mean calcium (Ca) concentration in leaf samples from the MARC site indicates that the Hass avocado variety had the highest Ca content, followed by the Ettinger variety, with the Fuerte variety showing the lowest overall concentration of Ca. Ca levels peaked in July, followed by June and May, with the lowest concentrations observed in February for all three avocado varieties. Comparing the two locations, the mean Ca content in Hass avocado leaves was higher at the MARC site than at the Mojo site.

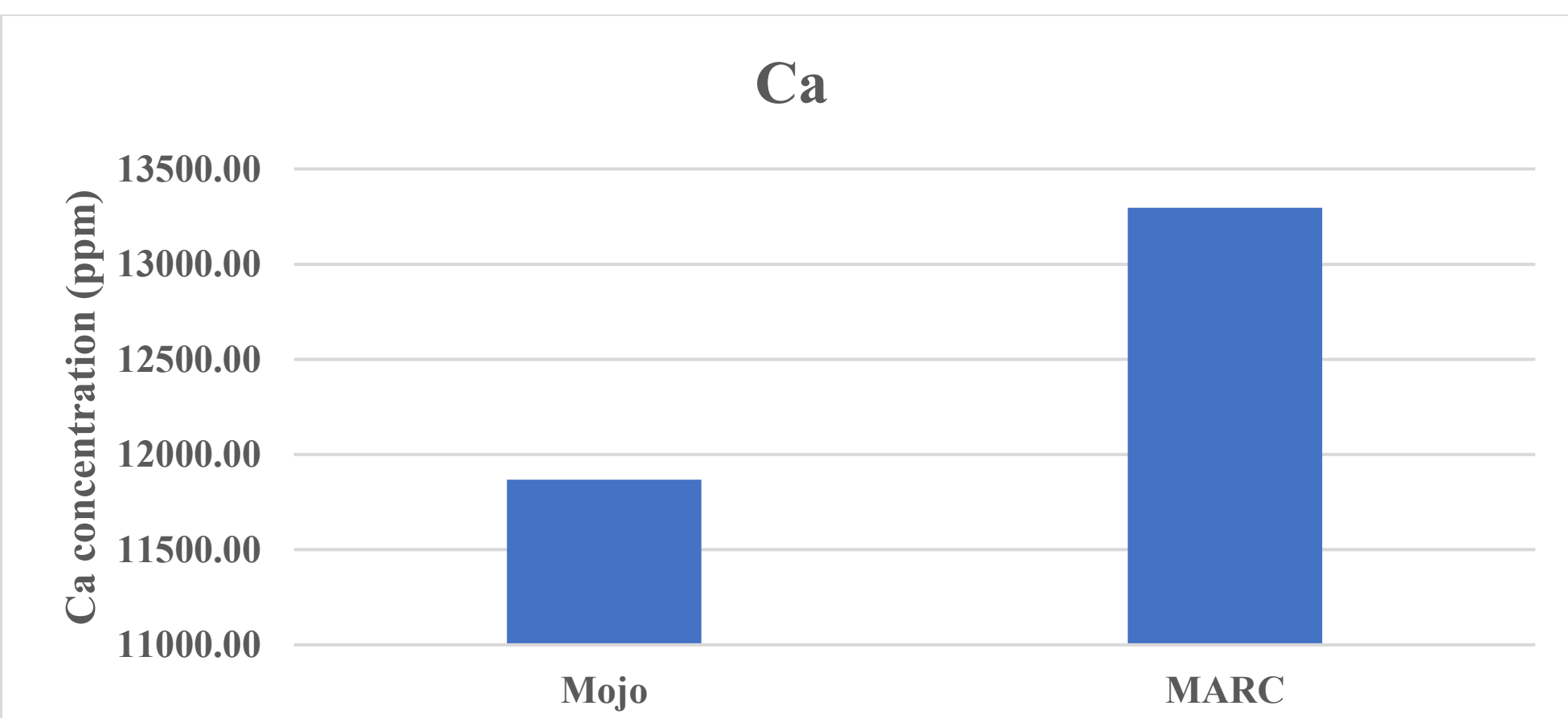

Figure 14: Level of Ca in Hass avocado variety leaves at Mojo and MARC.

3.6. Correlation analysis and antagonistic properties of macro and micronutrients

The Pearson correlation analysis among macro and micronutrients in avocado variety leaves across different sites revealed key relationships and interactions. The strongest correlations were observed between Mn and Cu (0.91), Zn and Fe (0.91), and Cu and Zn (0.82) in Hass avocado leaves at the Mojo site, indicating a tendency for these nutrients to increase together at that particular area. Conversely, the weakest correlations involved calcium with

the other nutrients which showed minimal interaction (Table 6 a), suggesting that calcium levels in Hass avocado leaves at the Mojo site are largely independent of the other nutrients. These results align with findings by authors [10], highlighting how soil chemistry and nutrient interactions influence uptake.

For the MARC site, Mn in Hass avocado leaves showed strong positive correlations with Cu (0.85) and Ca (0.86), indicating that Mn levels were linked to increases in these nutrients. Zn and Fe had a negative relationship (-0.68), which may imply antagonistic effects (Table 6 b). TN had a strong negative correlation with Zn (-0.95) in Fuerte avocado leaves at MARC, and Mn had positive relationships with both Cu (0.91) and Ca (0.84) (Table 6 c). Ettinger avocado leaves at MARC also exhibited positive correlations between Mn and Cu (0.65), Mn and Fe (0.50), and Mn and Ca (0.80), underscoring Mn's pivotal role in nutrient dynamics (Table 6 d).

Overall, across the four correlation matrices, the most consistent finding was the negative correlation between TN and Zn, suggesting nitrogen may suppress zinc uptake. Mn consistently showed positive correlations with Cu and Ca, indicating that adequate Mn levels are crucial for nutrient interactions in avocado leaves.

Table 6: Pearson correlation matrix of leaf nutrients (TN, Mn, Zn, Cu, Fe, Ca) in (a) Hass avocado at the Mojo site, (b) Hass avocado at MARC, (c) Fuerte avocado at MARC, and (c) Ettinger avocado at MARC.

(a)

Nutrients	TN	Mn	Zn	Cu	Fe	Ca
TN	1.00	-0.47	-0.99*	-0.77	-0.95*	-0.25
Mn		1.00	0.52	0.91	0.18	0.28
Zn			1.00	0.82	0.91	0.13
Cu				1.00	0.53	0.17
Fe					1.00	0.25
Ca						1.00

(b)

Nutrients	TN	Mn	Zn	Cu	Fe	Ca
TN	1.00	-0.05	0.19	0.19	0.02	-0.39
Mn		1.00	0.38	0.85*	-0.26	0.86*
Zn			1.00	0.46	-0.68	0.07
Cu				1.00	0.05	0.77
Fe					1.00	0.12
Ca						1.00

(c)

Nutrients	TN	Mn	Zn	Cu	Fe	Ca
TN	1.00	-0.63	-0.95*	-0.45	0.55	-0.12
Mn		1.00	0.72	0.95*	-0.22	0.84
Zn			1.00	0.63	-0.53	0.29
Cu				1.00	-0.15	0.91*
Fe					1.00	0.19
Ca						1.00

(d)

Nutrients	TN	Mn	Zn	Cu	Fe	Ca
TN	1.00	-0.36	-0.08	-0.44	-0.02	0.02
Mn		1.00	0.32	0.65	0.50	0.80
Zn			1.00	0.76	0.08	0.03
Cu				1.00	-0.13	0.49
Fe					1.00	0.13
Ca						1.00

*. Correlation is significant at the 0.05 level (2-tailed).

4. Discussion

4.1. Soil Nutrient Status at Mojo Site

The pH of the soil at the Mojo site which is slightly alkaline allows for the avocado cultivation. The CEC values at the Mojo site are consistently high, ranging from 30.72 to 39.84 meq/100g soil (mean: 34.14–38.40 meq/100g soil), indicating a strong capacity to retain and supply cationic nutrients such as calcium (Ca^{2+}), magnesium (Mg^{2+}), potassium (K^{+}), and ammonium (NH_4^{+}). These high CEC values are likely due to the presence of clay minerals

and moderate organic matter content, which provide abundant negatively charged sites for cation exchange [14, 38, 41]. However, the organic matter (OM) content at the site is rated as low to moderate, with values ranging from 0.64–1.74% TOC and 1.10–2.99% OM [33, 35]. Despite the moderate OM levels, the high CEC ensures a steady supply of nutrients to plants, reducing the risk of nutrient leaching and improving long-term soil fertility [43, 44]. Recent studies have also highlighted the role of CEC in buffering against nutrient imbalances and enhancing the availability of micronutrients such as manganese (Mn) and copper (Cu) [22, 46, 47]. The high CEC at the Mojo site likely contributes to the observed higher concentrations of these micronutrients in Hass avocado leaves compared to the MARC site. The low nitrogen content across all sites could limit plant growth, as nitrogen is essential for plant development. The moderate OC levels in most fields suggest good soil structure, fertility, and water-holding capacity [36, 39, 40], while the low OC levels in one field may indicate limited organic matter content, which could impact soil health and fertility.

The EC and CEC values increase with depth, which may be due to the accumulation of soluble salts in the soil. The EC values in the sampling areas found in the normal range indicate no risk of salinity as a result total soluble salts in the soil does not affect crop growth, making the soil suitable for avocado cultivation [1]. The medium copper content in the soil was found to be adequate for plant growth, as copper is essential for enzyme activity and reproductive growth in plants. To further contextualize these findings, the relationship between copper availability, soil pH, and electrical conductivity (EC) was examined. Copper availability is strongly influenced by soil pH, with acidic conditions typically enhancing its solubility and alkaline conditions reducing it. In this study, the soil pH ranged from 7.24 to 7.85, indicating a slightly alkaline environment. While alkaline conditions can limit copper availability, the observed pH range remains within a suitable level for adequate copper uptake by plants, as supported by recent studies [48, 49]. Additionally, the electrical conductivity (EC) of the soil, which reflects salinity levels, ranged from 98.8 to 218 µS/cm. These EC values are well below the threshold that could induce salinity stress, ensuring that copper availability and plant growth are not adversely affected. The combination of optimal pH, low EC, and adequate copper content creates a favorable environment for plant growth and development. Copper is a vital micronutrient for plants, playing a critical role in various physiological processes. It is an essential component of several enzymes, including cytochrome c oxidase, superoxide dismutase, and plastocyanin, which are involved in electron transport, antioxidant defense, and photosynthesis, respectively [50]. Copper is also crucial for reproductive growth, as it contributes to pollen viability, flower development, and seed formation [50]. In this study, the adequate copper content in the soil supports these enzymatic and reproductive functions, ensuring optimal plant growth and development.

Soil pH plays a critical role in iron availability, with alkaline conditions ($pH > 7$) typically reducing iron solubility and uptake by plants [49, 51]. In this study, the soil pH ranged from 7.24 to 7.85, which may explain the limited

iron availability. Alkaline soils can cause iron to form insoluble compounds, making it inaccessible to plants [41, 48]. These findings suggest that the observed iron deficiency is likely linked to the slightly alkaline soil pH, highlighting the need for targeted interventions, such as iron fertilization or soil acidification, to improve iron availability and prevent chlorosis. However, the nitrogen content in the sample appears to be lower than optimal, which may limit the efficiency of Mn in supporting these processes, particularly nitrogen assimilation [1, 2]. This contrast suggests that while Mn levels are adequate, the availability of nitrogen could be a limiting factor for overall plant growth and metabolic functions [3, 4]. Additionally, the observed decrease in manganese and copper concentrations with soil depth is consistent with the typical distribution of micronutrients, as topsoil generally contains higher organic matter and nutrient levels [9]. The very low to medium zinc levels further highlight potential nutrient imbalances, as zinc is essential for enzyme function and protein synthesis [34]. These findings underscore the importance of considering nutrient interactions and their collective impact on plant health.

Avocado Leaf Sample Analysis

The fluctuation of nitrogen levels in Hass avocado leaves across different months and locations indicates that nitrogen availability may be influenced by seasonal changes, environmental conditions, and management practices. The lower nitrogen levels at MARC compared to Mojo suggest that the MARC site may require additional fertilizer application to meet the nutritional needs of the avocado trees. Furthermore, the lower total nitrogen (TN) levels in the Ettinger variety suggest that this variety may have a lower nitrogen demand or that it is less efficient in nitrogen uptake compared to Hass.

In addition to these factors, the redistribution of nitrogen within the plant should be considered. Nitrogen is a mobile nutrient, and its redistribution from older to younger leaves or other growing tissues is a critical physiological process that supports growth during periods of limited nitrogen availability [52, 53]. This redistribution mechanism may explain the observed differences in nitrogen levels between varieties and locations, as some varieties may be more efficient in remobilizing nitrogen to meet metabolic demands.

Therefore, while external factors such as fertilization and environmental conditions play a significant role, intrinsic factors like nitrogen redistribution efficiency should also be considered when evaluating nitrogen dynamics in avocado trees. This holistic approach can help optimize nitrogen management strategies to improve tree health and productivity.

The variation in micronutrient concentrations in the leaf samples of the three avocado varieties across different sampling months indicates that nutrient availability is influenced by seasonal changes, soil conditions, and management practices. The higher micronutrient concentrations in the Ettinger variety suggest that this variety may have a higher demand for these nutrients or that it is more efficient in micronutrient uptake. The minimal levels of micronutrients in this study on February and March may be due to lower nutrient availability in the soil during these months or reduced uptake by the plants. The micronutrient levels found in the soil also indicate that very low Fe, very low to medium Zn, medium Cu, and medium to high lovel of Mn were observed (Table 4).

Overall, the leaf analysis results indicate that nutrient concentrations in avocado leaves fluctuate based on the season, environmental conditions, and the specific variety of the plant. Therefore, there are no consistent trends in the nutrient status of avocado leaves unless the varieties and soil environments are similar. This suggests that site-specific management practices may be required to optimize nutrient availability and uptake for different avocado varieties.

4.2. Correlation analysis

The results from the Pearson correlation analysis indicate several important trends in the nutrient interactions of avocado leaves, which have significant implications for nutrient management in avocado cultivation. The strong positive correlations observed between Mn and Cu, Zn and Fe, and Cu and Zn suggest that these nutrients have synergistic relationships. This implies that when one of these nutrients increases, the others tend to rise as well, which could be advantageous in designing fertilization programs aimed at boosting multiple micronutrient levels simultaneously.

In contrast, the negative correlation between TN and Zn observed across multiple avocado varieties points to a potential antagonism, where increased nitrogen levels may inhibit zinc uptake. This antagonism could have critical consequences for plant health, as zinc is essential for various physiological processes, including enzyme activation and protein synthesis. Excessive nitrogen fertilization could, therefore, negatively impact zinc availability, requiring careful nutrient management to avoid deficiencies.

The consistent finding that Mn positively correlates with both Cu and Ca across different avocado varieties suggests that manganese plays a central role in the nutrient dynamics of avocado leaves. Mn appears to facilitate the uptake of these essential micronutrients [3, 22, 34], making it crucial for maintaining the balance of nutrients in the plant. The positive correlation between Mn and Ca is particularly noteworthy, as calcium is vital for cell wall stability and other physiological processes [32]. Ensuring adequate levels of Mn may help enhance calcium availability, promoting better overall plant health.

The competition between Zn and Fe, as suggested by their negative correlation, indicates that these nutrients may compete for uptake or transport within avocado plants. This antagonistic relationship could lead to imbalances if one nutrient is present in excessive amounts, underscoring the importance of balanced fertilization strategies.

The strong correlation between Cu and Ca, especially in Fuerte and Ettinger avocado varieties, suggests that Cu availability may influence calcium levels, which is crucial for maintaining proper physiological functions such as cell wall integrity [13, 32]. Managing copper levels could therefore play a role in optimizing calcium availability.

In conclusion, this study highlights several important nutrient interactions in avocado leaves, including the antagonistic relationship between TN and Zn, the synergistic effects of Mn with Cu and Ca, and the competition

between Zn and Fe. Understanding these correlations is essential for optimizing nutrient management practices, ensuring balanced nutrient availability, and promoting healthy avocado plant growth.

4.3. Implications for Nutrient Management in Avocado Cultivation

The findings of this study underscore the importance of targeted nutrient management strategies for avocado cultivation, particularly in specific locations characterized by nutrient deficiencies. The critically low levels of Fe and Zn observed in Mojo soil need for supplementation. Foliar applications or soil amendments with chelated forms of Fe and Zn could be effective in enhancing their availability and uptake by avocado plants [44]. Furthermore, the consistently low TN levels across the study sites highlight the importance of applying nitrogen-rich fertilizers, especially during critical growth stages, to support optimal plant development. The observed nutrient variability across locations and seasons suggests that a one-size-fits-all approach to fertilization may not be effective. Tailored fertilization schedules should consider site-specific soil nutrient status, variety-specific nutrient demands, and seasonal dynamics. Regular monitoring through leaf nutrient analysis and soil testing is vital for tracking seasonal nutrient fluctuations. Such practices enable timely adjustments to fertilization programs, ensuring that nutrient availability is maintained throughout the growing season. Additionally, integrating organic and inorganic nutrient sources can offer multiple benefits, such as enhancing soil organic carbon levels, improving nutrient retention, and promoting overall soil health. These practices are particularly crucial for fields with low organic carbon content, where soil structure and fertility could be improved through organic matter amendments.

5. Conclusion

The soil analysis of Mojo avocado farms revealed very low iron (Fe) and zinc (Zn) levels, moderate manganese (Mn), copper (Cu), and organic carbon (OC), and low total nitrogen (TN). Leaf analysis across Hass, Fuerte, and Ettinger varieties showed TN levels in the order: Hass > Fuerte > Ettinger, with a declining trend at the Mojo site from February to June, while TN levels at the MARC site remained stable. Pearson correlation analysis identified key nutrient interactions, including synergistic relationships (Mn, Cu, Ca) and antagonistic interactions (TN-Zn, Zn-Fe). These findings highlight the need for improved nutrient management strategies in avocado cultivation.

To optimize nutrient management, continuous leaf sampling over at least one year is recommended to capture seasonal variations. Future studies should correlate leaf nutrient levels with agronomic outcomes like fruit yield, shelf life, and tree productivity, and evaluate nutrient removal through harvest to determine precise annual nutrient requirements. These steps will enhance targeted nutrient application and improve orchard productivity and fruit quality.

Acknowledgment

Ethiopian Institute of Agriculture Research is highly acknowledged for the provision of financial support for the Integrated Fruits Research Project through the Integrated Research Program from 2021-2023. Integrated Fruit Research Program is also highly acknowledged for facilitating activities and Melkassa Agricultural Research Center for assisting in implementing this research activity.

Author contributions: Mr. Zeyede Aregahegn: Collected field data, conducted laboratory analysis, performed data analysis and interpretation, wrote the manuscript, and prepared it for publication. **Dr. Edossa Etissa**: Proposed the research plan and supervised the project activities. **Dr. Dejene Abera** and **Mr. Israel Bekele**: Provided feedback on the manuscript.

Funding Not applicable.

Data availability Data produced during this study can be obtained from corresponding author on reasonable request.

Declarations

Ethical approval and consent to participate This article does not contain any studies with human participants or animals performed by any of the authors. All the plants used in our study were cultivars, and the material complied with national guidelines.

Consent for publication The authors have the consent to use the data for the publication of the article.

Competing interests The author have no competing interests to declare that are relevant to the content of this article.

References

1. Acosta-Rangel AM, Li R, Celis N, Suarez DL, Santiago LS, Arpaia ML, Mauk PA. The physiological response of 'Hass' avocado to salinity as influenced by rootstock. *Scientia horticulturae*. 2019;256:108629
2. Ahmed N, Zhang B, Chachar Z, Li J, Xiao G, Wang Q, Hayat F, Deng L, Bozdar B, Tu P. Micronutrients and their effects on horticultural crop quality, productivity and sustainability. *Scientia Horticulturae*. 2024;323:112512.
3. Alejandro S, Höller S, Meier B, Peiter E. Manganese in plants: from acquisition to subcellular allocation. *Frontiers in plant science*. 2020;*11*:300.
4. Bender GS, Faber B. Avocado fertilization. *University California Cooperative Extension. San Diego, CA*. 1999;https://ucanr.edu/sites/alternativefruits/files/121293
5. Benou K, Ioannou D, Moustakas N. Seasonal variations in leaf nutrient concentrations in three fig (Ficus carica L.) varieties. *Journal of Elementology*. 2020;25(4):563-1579.
6. Bibi S, Irshad M, Mohiuddin M, Sher S, Tariq MA, Ng AW. Distribution of phosphorus fractions in orchard soils in relation to soil properties and foliar P contents. Sustainability. 2022;14(7):3966.
7. Campisi-Pinto S, Zheng Y, Rolshausen PE, Crowley DE, Faber B, Bender G, Bianchi M, Khuong T, Lovatt CJ. Optimal nutrient concentration ranges of 'Hass' avocado cauliflower stage inflorescences—potential diagnostic tool to optimize tree nutrient status and increase yield. *HortScience*. 2017;52(12): 1707-1715.
8. Cazetta JO, Zanata RMP, Barbosa PWG. Seasonal nutrient content variation in avocado leaves of 'Geada','Fortuna'and 'Quintal'genotypes. *Revista Brasileira de Fruticultura*. 2023;45:e-425.
9. Dhaliwal SS, Sharma V, Kaur J, Shukla AK, Hossain A, Abdel-Hafez SH, Gaber A, Sayed S, Singh VK. The pedospheric variation of DTPA-extractable Zn, Fe, Mn, Cu and other physicochemical characteristics in major soil orders in existing land use systems of Punjab, India. *Sustainability*. 2021;14(1):29.
10. Dobrin A, Zugravu M, Moț A, Mușat IB, Ciceoi R. Macro and micronutrients distribution in calcaric aluviosoil. 2020;63(1)https://agronomyjournal.usamv.ro/pdf/2020/issue_1/Art8
11. Estefan G, Sommer R, Ryan J. Methods of soil, plant, and water analysis. *A manual for the West Asia and North Africa region*. 2013;3: 65-119.
12. Farooq QUA, McComb J, Hardy GE, Burgess TI. Soil amendments for management of Phytophthora root rot in avocado and their impact on the soil microbiome. *Journal of Plant Pathology*. 2024;1-17.

13. Gilliham M, Dayod M, Hocking BJ, Xu B, Conn SJ, Kaiser BN, Leigh RA, Tyerman SD. Calcium delivery and storage in plant leaves: exploring the link with water flow. *Journal of experimental botany*. 2011;62(7): 2233-2250.
14. Hazelton P, Murphy B. Interpreting soil test results: What do all the numbers mean? 3rd ed. CSIRO Publishing; **2016**.
15. Jalata Z. Current status, potentials and opportunities of avocado production as an alternative crop: The case of Ethiopia: A review. *Agricultural Reviews*. 2021;42(3): 336-341.
16. Joyce DC. Improved fruit robustness and quality in avocado supply chains (mineral nutrition). Department of Agriculture and Fisheries Queensland (DAF). 2021;4-13.
17. Lovatt C. Optimizing 'Hass' avocado tree nutrient status to increase grower profit-an overview. In *Actas del VIII Congreso Mundial de la Palta*. 2015;17-23.
18. Malik AR, Ahmad MF, Ganai MA, Bangroo SA, Nazir N, Khalik A, Raja RHS. Seasonal variation in leaf mineral nutrient and optimization of sampling dates for better indexing of pear leaf. *Int. J. Curr. Microbiol. Appl. Sci*. 2019;8:1152-1157.
19. Newett S, Rigden P, Carr B (2018) Avocado Plant Nutrition Review. *Nambour: Department of Agriculture and Fisheries*, Queensland, Australia. 2018.
20. Pansu M, Gautheyrou J. Mineralogical separation by selective dissolution. *Handbook of soil analysis: mineralogical, organic and inorganic* methods. 2006;561-569.
21. Prashar N, Bakshi M, Shilpa C, Ughareja A. Role of micro-nutrients in fruit production: A review. *The Pharma Innov. J*. 2022;*11*(6):1158-1164.
22. Rashed MH, Hoque TS, Jahangir MMR, Hashem MA. Manganese as a Micronutrient in Agriculture: Crop Requirement and Management. *J. Environ. Sci. & Natural Resources*. 2019;12(1&2):225-242.
23. Rebolledo-Roa A, Burbano-Diaz RA. Nutrient balance in hass avocado trees as a tool to optimize crop fertilization management. *Agronomy*. 2023;*13*(8):1956.
24. Richard R, Carol JL. Seasonal patterns of nutrient uptake and partitioning as a function of crop load of the "Hass" avocado. California Department of Food and Agriculture Fertilizer Research and Education Program (FREP). 2004.
25. Salazar-García S, González-Durán IJ, Ibarra-Estrada ME. Identification of the appropriate leaf sampling period for nutrient analysis in 'Hass' avocado. *HortScience*. 2015;50(1):130-136.
26. Salvatore C, Yusheng Z, Philippe ER, David EC, Ben F, Gary B, Mery B, Toan K, Carol JL. Optimal Nutrient Concentration Ranges of 'Hass' Avocado Cauliflower Stage Inflorescences—Potential Diagnostic Tool to Optimize Tree Nutrient Status and Increase Yield. *HORTSCIENCE*. 2017;52(12):1707–1715.

27. Samuel S, Isidro G. Identification of the Appropriate Leaf Sampling Period for Nutrient Analysis in 'Hass' Avocado. *HORTSCIENCE*. 2015;50(1):130–136.
28. Sikora FJ, Moore KP. Soil test methods from the southeastern United States. *Southern cooperative series bulletin*. 2014;419:54-58.
29. Silber A, Naor A, Cohen H, Bar-Noy Y, Yechieli N, Levi M, Noy M, Peres M, Duari D, Narkis K, Assouline S. Avocado fertilization: Matching the periodic demand for nutrients. *Scientia Horticulturae*. 2018;241:231-240.
30. Soil quality – Agronomy technical note No. 8. Liming To Improve Soil Quality in Acid Soils. USDA, Natural Resources Conservation Service. **1999**.
31. Soil Quality Information Sheet: Soil Quality Indicators: pH. USDA, Natural Resources Conservation Service. **1998**.
32. Thor K. Calcium—nutrient and messenger. *Frontiers in plant science*. 2019;(*10*):440.
33. Wogi L, Dechassa N, Haileselassie B, Mekuria F, Abebe A, Tamene LD. A guide to standardized methods of analysis for soil, water, plant, and fertilizer resources for data documentation and sharing in Ethiopia. *CIAT Publication*. **2021**.
34. Yomso and Baharli. Roles of manganese in plants at physiological and biochemical perspectives. *Pharma Innovation*. 2021;10(2):53-66.
35. Hazelton PA and Murphy BW. Interpreting Soil Test Results: What Do All the Numbers Mean? CSIRO Publishing, Australia. **2007**;1-152.
36. Charman PEV and Roper MM. Soil Organic Matter. In: Charman, P.E.V. and Murphy, B.W., Eds., Soils—Their Properties and Management, 3rd Edition, Oxford University Press, Melbourne. **2007**;276-285.
37. Gonzalez-Vences R, Cajuste-Bontemps L, Sanchez-Escudero J, Gomez-Merino FC, Trejo-Tellez LI. Efficient detection of nutrient deficiencies and development of corrections in avocado through the Compositional Nutrient Diagnosis (CND). *Notulae Botanicae Horti Agrobotanici Cluj-Napoca*. 2024;*52*(1): 13118-13118.
38. Yadav SPS, Bhandari S, Bhatta D, Poudel A, Bhattarai S, Yadav P, Ghimire N, Paudel P, Paudel P, Shrestha J, Oli B. Biochar application: A sustainable approach to improve soil health. *Journal of Agriculture and Food Research 11*. 2023;100498.
39. Manns HR, Parkin GW, Martin RC. Evidence of a union between organic carbon and water content in soil. *Canadian Journal of Soil Science*. 2016;*96*(3): 305-316.

40. Ramírez PB, Machado S, Singh S, Plunkett R, Calderón FJ. Addressing the effects of soil organic carbon on water retention in US Pacific Northwest wheat–soil systems. *Frontiers in Soil Science*. 2023;*3*:1233886.
41. Selladurai R, Awachare CM. Nutrient management for avocado (Persea americana miller). *Journal of plant nutrition*. 2019;*43*(1):138-147.
42. Gebrekirstos A, Hadgu K, Mokria M, Hagazi N, Dubale W. Adaptation and growth performance of five avocado cultivars in Ethiopia. *Heliyon*. 2023;*9*(12): 1-11.
43. Shumeta Z. Avocado Production and Marketing in Southwestern Ethiopia. *Trends Agric Econ*. 2010;3:190–206.
44. Cruz-Álvarez O, Sánchez-Chávez E, Benavides-Mendoza A, Hernández-Rodríguez OA, Parra-Quezada RA, Ciscomani-Larios JP, Martínez-Damián MT, Ojeda-Barrios DL. Foliar applications of zinc oxide nanoparticles and boric acid affect leaf oxidative metabolism and productivity in young pecan trees. *Heliyon*. 2024;10(15):1-12.
45. Edossa E, Lemma A, Asmare D, Merkebu A, Girma K, Agarnesh M, Wegayehu A. Phenological Growth Patterns of Commercial Avocado (*Persea americana* M.) Varieties for Agronomic Managements Practices at Melkassa, Central Rift Valley of Ethiopia; Paper Presented on the Crop Science Society of Ethiopia (*CSSE*). 19th Biannual Conference of *CSSE* 24-24 Feb. 2023, Addis Ababa, Ethiopia.
46. Ćirić V, Prekop N, Šeremešić S, Vojnov B, Pejić B, Radovanović D, Marinković D. The implication of cation exchange capacity (CEC) assessment for soil quality management and improvement. *Agriculture and Forestry*. 2023;69 (4): 113-133.
47. Landon JR. Booker tropical soil manual: a handbook for soil survey and agricultural land evaluation in the tropics and subtropics. *Routledge*. 2014.
48. Mohiuddin M, Irshad M, Sher S, Hayat F, Ashraf A, Masood S, Bibi S, Ali J, Waseem M. Relationship of selected soil properties with the micronutrients in salt-affected soils. *Land*. 2022; 11(6):845.
49. Barrow NJ, Hartemink AE. The effects of pH on nutrient availability depend on both soils and plants. *Plant and Soil*. 2023; 487(1):21-37.
50. Yruela I. Copper in plants: acquisition, transport and interactions. *Functional Plant Biology*. 2009; 36(5):409-30.
51. Kobayashi T, Nishizawa NK. Iron uptake, translocation, and regulation in higher plants. *Annual review of plant biology*. 2012; 63(1):131-52.
52. Zayed O, Hewedy OA, Abdelmoteleb A, Ali M, Youssef MS, Roumia AF, Seymour D, Yuan ZC. Nitrogen journey in plants: From uptake to metabolism, stress response, and microbe interaction. *Biomolecules*. 2023;13(10):1443.

53. Plett DC, Ranathunge K, Melino VJ, Kuya N, Uga Y, Kronzucker HJ. The intersection of nitrogen nutrition and water use in plants: new paths toward improved crop productivity. *Journal of experimental botany*. 2020; 71(15):4452-68.
