# Supplementary material for: Seasonal dynamics of leaf macro- and micronutrients in three commercial avocado varieties at Mojo and Melkassa, Ethiopia
Source: PLoS One. 2026 Nov 12;21(11):e0362564. doi: 10.1371/journal.pone.0362564 (PMC13759051; doi:10.1371/journal.pone.0362564)
Supplement: Supplementary file 3 [file pone.0362564.s006.docx]

General

The paper addresses an assessment of selected essential macro and micronutrients in commercial avocado varieties and soil nutrient status of avocado farms. The study is based on orchard experimentation nutrient status of avocado varieties across locations and fruit tree age. It also assess soil nutrient status in the farm.

However, the paper needs major revision to improve data analysis, result presentation, the coherence, and language improvement. The results need to be rewritten separately for Mojo and Melkassa sites without redundancy/repetition of results and discussion sections. Check and correct figures and Tables in the results.

Introduction

1. 1^st^ paragraph, insert citation
2. Thorough language revision

Materials and methods

1. A revision is required mainly for data analysis section.
2. Please indicate the year of data collection.
3. Data collection and analysis. The first sentence is very fragmented, please move to second paragraph. Please rework this section so that the soil sampling and agronomy data collection can be presented in a separate section. Is the soil analyzed before planting?

Results

1. Under the results section, the authors present Correlation analysis. But, it should not be tittle.
2. Figures. Reanalysis and generate all graphs
3. To ensure coherent flow of results, I suggest to present separately for for Mojo and Melkassa sites.

Discussion

Refine tittles and make coherent among sections.

Conclusion

It seems summary and need to add implications of the study.
