# Supplementary material for: Seasonal dynamics of leaf macro- and micronutrients in three commercial avocado varieties at Mojo and Melkassa, Ethiopia
Source: PLoS One. 2026 Nov 12;21(11):e0362564. doi: 10.1371/journal.pone.0362564 (PMC13759051; doi:10.1371/journal.pone.0362564)
Supplement: Supplementary file 4 [file pone.0362564.s007.docx]

**Response to Reviewers**

**Manuscript PONE-D-26-25421**

Original title: “Levels and seasonal patterns of selected essential macro and micronutrients in commercial avocado varieties and soil nutrient status of avocado farms at Mojo and Melkassa sites, Ethiopia”

Revised title: “Seasonal dynamics of leaf macro- and micronutrients in three commercial avocado varieties at Mojo and Melkassa, Ethiopia”

Dear Dr. Raed Abduljabbar Haleem, Academic Editor,

Thank you for the opportunity to revise our manuscript, and thank you to both reviewers for their careful and helpful comments. We have addressed every point. Most importantly, following Reviewer #2, we re-analysed all leaf data from the tree-level raw data, and this reanalysis changed some of our conclusions. Below we first summarize the main changes and then respond point by point. Reviewer comments are in italics and our responses in blue. All changes are shown in the file “Revised Manuscript with Track Changes”. Section names refer to the revised manuscript, which now uses unnumbered headings.

# Summary of the main changes

- Statistical analysis completely redone (Reviewer #2). The tagged tree is now the experimental unit. We fitted linear mixed models with tree as a random effect (repeated sampling of the same trees), checked the model assumptions, used Tukey’s test on estimated marginal means for mean separation, and report mean ± SE for every group (Tables 4–8 and Figs 2–4).
- Correlations re-analysed with repeated-measures correlation on all tree × month observations (36–45 per group) with Benjamini–Hochberg adjustment, instead of Pearson correlations on four or five monthly means (new Fig 5 and S2 Table).
- Errors found and corrected while re-checking the data: one monthly mean had been transcribed wrongly (Zn at Mojo in March: 15.56 mg/kg used instead of the correct 36.94 mg/kg).
- Conclusions revised to match the reanalysis: leaf TN was below the sufficiency range at both sites, but the differences between varieties, months and sites were not statistically significant. Leaf Ca increased strongly during the season; micronutrients showed variety-specific seasonal patterns; Mn was positively correlated within trees with Ca in all varieties and with Cu and Zn in Hass and Fuerte.
- Results restructured by site: soil at Mojo and at MARC, Mojo Hass leaves, MARC three varieties, comparison of Hass between sites, sufficiency ranges and relationships among nutrients. The 14 earlier figures were replaced by 4 new multi-panel figures (Figs 2–5), and a map of the study area was added as Fig 1.
- Language revised throughout following Reviewer #1, and redundant text removed from the Introduction, Methods and Discussion. A limitations paragraph and the practical implications were added.
- Soil data for MARC added (new Table 5): the leaf samples at MARC can now be interpreted against soil data from three points in the MARC avocado orchard, measured with the same methods and at the same depths (0–20 and 20–50 cm) as at Mojo. Because the two sites lie in different agroecological settings, the soils of each site are rated against the standard classes used in Ethiopia (the Ethiopian guide of Wogi et al. 2021, Landon 2014, and Lindsay and Norvell 1978) instead of being compared statistically, and the same rating system is now used for both soil tables. The agroecology of both sites (rainfall, temperature and rainy seasons) is described briefly in “Study sites”. As the main focus of the study is the leaf nutrients, the soil is no longer named in the title.
- Journal requirements: PLOS ONE style (title page, line numbers, captions, Vancouver references renumbered in order of citation), field permit and ethics statement in Methods, funding removed from the Acknowledgments, data and R code provided as Supporting Information (S1 Dataset, S1 Code, S1 and S2 Tables).

# Response to the Journal Requirements

**Comment 1:** *Please ensure that your manuscript meets PLOS ONE’s style requirements, including those for file naming.*

**Response:** We reformatted the manuscript following the PLOS ONE templates: title page with full and short titles. We also follow the journal style including; keywords and the author contributions, funding, data availability, competing interests and declaration statements removed from the manuscript file (they are given in the submission form, and the ethics statement is in Methods); table and figure captions in the “Table 1.” / “Fig 1.” format placed after the paragraph where they are first cited; figures removed from the manuscript file and uploaded separately as Fig1.tif–Fig5.tif; a supporting information section with captions at the end. We added references for the statistical and analytical methods and for two recent Ethiopian studies on avocado and soil. We also replaced the reference column of the soil table, with a footnote giving the sources of the soil ratings (Table 4), corrected the author names of two references after checking them against the original sources (Seid et al. 2023; Rosecrance and Lovatt 2004).

**Comment 2:** *Please note that PLOS One has specific guidelines on code sharing ...*

**Response:** All R code used for the statistical analyses and figures is provided without restriction as S1 Code (a zip file with the scripts, the input data and a README). The scripts reproduce all tables, statistics and figures from S1 Dataset.

**Comment 3:** *In your Methods section, please provide additional information regarding the permits you obtained for the work ...*

**Response:** We added a new subsection, “Field permits and ethics statement”: “Sampling at MARC was carried out in the Center’s own experimental orchard with the approval of the Melkassa Agricultural Research Center, Ethiopian Institute of Agricultural Research (EIAR). Leaf and soil samples on the privately owned farms at Mojo were collected with the oral consent of the farm owners, who volunteered to take part in the study and are familiar with the research activities of MARC and EIAR. No other permits were required because the work was done on cultivated land and did not involve protected areas or protected or endangered species.”

**Comment 4:** *Please remove any funding-related text from the manuscript and let us know how you would like to update your Funding Statement.*

**Response:** The funding sentence was removed from the Acknowledgments, and the “Funding” section was removed from the manuscript. The amended Funding Statement is given in the cover letter and below (Comment 5).

**Comment 5:** *Please clarify the sources of funding (a–d) ...*

**Response:** Amended Funding Statement: “This study was funded by the Ethiopian Institute of Agricultural Research (EIAR) through the Integrated Fruit Research Program (2021–2023). All authors were employees of EIAR and received salaries from EIAR during the study. Apart from employing the authors, the funder had no additional role in study design, data collection and analysis, decision to publish, or preparation of the manuscript. No specific funding was received for the preparation or publication of this manuscript.”

**Comment 6:** *Please include your full ethics statement in the ‘Methods’ section ...*

**Response:** The ethics statement is now in Methods (section “Field permits and ethics statement”): the study did not involve human participants or animals, so approval by an ethics committee (IRB) was not required and informed consent for research participation was not applicable; the farm owners gave oral consent for access to their orchards.

**Comment 7:** *Data availability ...*

**Response:** All data underlying the findings are now provided as Supporting Information: S1 Dataset contains the tree-level leaf data (every tree in every month, with values below the detection limit marked ND), the soil data, and the summary tables used for the figures. The Data Availability Statement is now: “All relevant data are within the manuscript and its Supporting Information files.”

**Comment 8:** *Please amend the manuscript submission data (via Edit Submission) to include authors Edossa Etissa, Israel Bekele, Dejene Abera.*

**Response:** Done. The three co-authors were added to the submission data in Editorial Manager.

**Comment 9:** *If the reviewer comments include a recommendation to cite specific previously published works ...*

**Response:** We read the works suggested by Reviewer #1. They deal with GIS, remote sensing and geostatistical mapping of soil quality, salinity and land degradation. Our study did not use spatial or remote sensing data, so we did not find these works directly relevant and did not cite them (see also our response to Reviewer #1).

# Response to Reviewer #1 (Dr. Mohamed A. E. AbdelRahman)

We thank the reviewer for the detailed language and clarity suggestions. We accepted nearly all of them. Some sentences addressed by the reviewer were rewritten or removed during the reanalysis; this is stated where relevant.

## Title, Abstract and Keywords

**Comment 1:** *Title: “Levels and seasonal patterns…” → consider “Seasonal dynamics of macro- and micronutrients…”; “soil nutrient status of avocado farms” → “soil nutrient profiles in avocado orchards”.*

**Response:** Accepted in part. New title: “Seasonal dynamics of leaf macro- and micronutrients in three commercial avocado varieties at Mojo and Melkassa, Ethiopia”. We adopted “Seasonal dynamics”, as suggested. We did not keep the soil in the title, because the main aim of the study is the seasonal leaf nutrient status; the soil properties of both sites are presented in the Results (Tables 4 and 5) as a basis for interpreting the leaf data.

**Comment 2:** *Abstract: remove “highly productive” (redundant).*

**Response:** Done; the first sentence of the abstract was rewritten.

**Comment 3:** *Abstract: “0 ppm” → “below detection limit”.*

**Response:** Done. “0 ppm” was replaced by “below the detection limit” throughout; the Abstract now states that the soils of both sites were mostly very low to low in DTPA-extractable Zn and Fe.

**Comment 4:** *Abstract: break the Pearson correlation sentence into two sentences.*

**Response:** Done. The long correlation sentence was replaced by one short sentence based on the new repeated-measures correlation results (Mn was positively correlated with Ca in all varieties and with Cu and Zn in Hass and Fuerte).

**Comment 5:** *Abstract: final sentence (“Future studies…”) is speculative; move to Conclusion.*

**Response:** Done. The sentence was removed from the Abstract; future work is described in the Conclusion.

**Comment 6:** *Keywords: “Moj” typo; avoid mixing site names with general terms.*

**Response:** Thank you. PLOS ONE does not use keywords, so the keyword line was removed from the manuscript; the subject areas are selected in the submission form instead.

## Introduction

**Comment 7:** *“shows great potential” → “is well adapted”.*

**Response:** Done.

**Comment 8:** *Hass, Fuerte and Ettinger described twice; merge.*

**Response:** Done; merged into one short paragraph.

**Comment 9:** *“particularly prolific” → “highly productive”.*

**Response:** Done.

**Comment 10:** *Recast the sentence on macro- and micronutrient concentrations across phenological stages.*

**Response:** Done: “Macronutrient … and micronutrient … concentrations in fruit-tree leaves vary across phenological stages (flowering, fruit development, fruit maturity and post-harvest).”

**Comment 11:** *Photosynthesis explanation is basic; condense to one sentence.*

**Response:** Done.

**Comment 12:** *Remove “including” after the colon.*

**Response:** Done; the sentence was shortened and the repeated list removed.

**Comment 13:** *“appropriate timing” → “optimal fertilization schedule”.*

**Response:** Done.

**Comment 14:** *Last two paragraphs overlap; merge.*

**Response:** Done. They were merged into one paragraph that ends with three explicit objectives and the hypotheses tested.

**Comment 15:** *Overall: use “nutrient dynamics” or “nutrient status” consistently; avoid promotional phrases; replace vague terms (“good soil”, “healthy tree”).*

**Response:** Done. “Nutrient status” is used for the soil and leaf diagnosis and “seasonal dynamics/changes” for the time pattern. The phrase on stimulating commercial and economic activities was removed, and “good soil” was replaced by “soil conditions, such as adequate organic matter and a suitable pH”.

## Materials and Methods

**Comment 16:** *2.1: “specifically for the Hass variety” → “restricted to Hass variety”; simplify the MARC location sentence; use “coordinates”; merge location details concisely.*

**Response:** Done. The site description was rewritten into one concise paragraph with coordinates and altitudes for both sites.

**Comment 17:** *2.2: “cupper” → “copper”; depth sentence; “following standard protocols [11, 28, 33]”.*

**Response:** Done (section “Soil sampling and analysis”). Each method is now named with its reference: pH and EC in 1:2.5 soil:water, Walkley–Black organic carbon, Kjeldahl total N, ammonium acetate CEC, Bouyoucos hydrometer texture and DTPA micronutrients.

**Comment 18:** *2.3: “None shaded” → “Non-shaded”; “choosen” → “chosen”; “as it is the predominant commercial variety”; condense the repetitive sampling description.*

**Response:** Done (section “Sampling of avocado leaves”). The section was rewritten as one structured paragraph that also gives the year and months of sampling, the number of trees, the composite leaf sample per tree (about 16 leaves from four canopy directions, analysed in three replicates) and the missing samples.

**Comment 19:** *2.4: “grinded” → “ground”; avoid repeated “transferred”; “500 °C”.*

**Response:** Done (section “Leaf sample preparation and analysis”).

**Comment 20:** *2.5: simplify the FAAS sentence; “Digital furnace” → “Programmable furnace”.*

**Response:** Done.

**Comment 21:** *2.6: “serious of standards” → “series of standards”; “analytical-grade reagents”.*

**Response:** Done.

**Comment 22:** *2.7: revise calibration sentences; “Fe, Cu, Mn, and Zn standards”.*

**Response:** Done. While revising we also corrected the description: the soil standards were 0–2.0 mg/L (as in Table 2) and the leaf standards 0–10 mg/L (Table 3).

**Comment 23:** *2.8: refer to Table 1 in the text; clarify units (mg/L or µg/mL).*

**Response:** Done. A sentence now introduces Tables 1–3, and all solution concentrations are now given in mg/L (instead of ppm or µg/mL) and soil and leaf concentrations in mg/kg.

**Comment 24:** *2.9: “recovery determined by spiking leaf and soil extracts”; “spiking”.*

**Response:** Done; the heading is now “Recovery analysis”.

**Comment 25:** *2.10: equations (2) and (3) misaligned; remove comma after “Where”.*

**Response:** Done. The equations are now set with a centre tab and a right-aligned equation number, and “where” follows without a comma. We also stated that δ is the standard deviation of the blank measurements.

**Comment 26:** *2.11: simplify the data analysis sentence; “analyzed using R software”.*

**Response:** This section (“Statistical analysis”) was completely rewritten in response to Reviewer #2; see below.

## Results

**Comment 27:** *3.1: “reviled” → “revealed”; “within acceptable range for avocado growth”; “copper”; condense soil texture to one sentence.*

**Response:** Done. The soil texture is summarized in one sentence (“mainly clay loam, with clay texture at point P2 of farm 3”). We also removed the statement that the soil provides adequate P and K, because P and K were not measured.

**Comment 28:** *3.2: “occured”, “peored”; use “Total nitrogen (TN)” consistently; Figures 1–6 descriptions are repetitive.*

**Response:** Done. The six TN figures were replaced by Figs 2–4, which show all nutrients together, and TN is used consistently.

**Comment 29:** *3.3: “Cu and Fe peaked in July”; “minimal” → “lowest”; combine with the comparison table instead of repeating ranges.*

**Response:** Done. The two original micronutrient sections (3.3 and 3.4 of the first submission) were rewritten after the reanalysis; the text now gives the main patterns and refers to Table 7 for the values.

**Comment 30:** *3.4: “Zn increased in May–June”; “initially low or absent” → “below detection limit”; Figures 8–12 overlap.*

**Response:** Done. Figures 8–12 were replaced by Figs 2–4, and “below the detection limit” is used throughout.

**Comment 31:** *3.5: Ca wording; use “Ca concentration (%)” consistently.*

**Response:** Done. Ca is reported in % dry weight throughout (the former Figure 14 used ppm).

**Comment 32:** *3.6: “authors [10]” → “previous studies [10]”; “minimal interaction” → “weak correlation”; summarize matrices in text; uniform reporting of correlation strength.*

**Response:** Done. The correlation section was rewritten with the new repeated-measures correlations. The text summarizes the significant correlations and the full matrices are shown in Fig 5 and S2 Table, with the strength reported as r_rm values.

**Comment 33:** *Overall: replace vague terms (“adequate”, “normal range”) with thresholds; standardize units (ppm vs %); streamline figure captions.*

**Response:** Done. New Table 8 compares the leaf values with numerical optimum ranges, and the text gives thresholds (e.g., TN 1.6–2.2%, Zn 30–150 mg/kg). TN and Ca are given in % and micronutrients in mg/kg throughout. Captions were shortened.

## Discussion and Conclusion

**Comment 34:** *4.1: simplify the pH sentence; “classified as”; copper functions described twice; use “organic carbon (OC)” consistently; condense EC and CEC; “lovel” → “level”.*

**Response:** Done. The section, now “Soil properties of the study sites”, was shortened to two paragraphs: one on the soils of both sites and one relating soil and leaf nutrient status within each site.

**Comment 35:** *Leaf analysis: simplify the nitrogen sentence; “lower nitrogen uptake efficiency”; condense redistribution to one sentence.*

**Response:** Done. Because the reanalysis showed that the TN differences between varieties and sites were not significant, we no longer claim lower N uptake efficiency in Ettinger. Redistribution is now covered in one sentence (section “Seasonal and varietal patterns in leaf nutrients”).

**Comment 36:** *4.2: “may impair zinc-dependent processes”; “is pivotal”; use “positive/negative correlation” consistently; merge Cu–Ca with Mn–Ca.*

**Response:** Done. With the corrected analysis, the TN–Zn and Zn–Fe negative correlations were not significant, so the antagonism paragraphs were removed. The section now uses “positive/negative correlation” and discusses Mn, Ca, Cu and Zn together.

**Comment 37:** *4.3: “require supplementation”; “uniform fertilization strategy”; “leaf nutrient analysis” consistently; condense organic amendments.*

**Response:** Done (section “Implications for nutrient management”).

**Comment 38:** *Overall (Discussion): replace vague terms with thresholds or references; standardize units; streamline descriptive paragraphs.*

**Response:** Done. Soil and leaf values are now judged against stated classes and published ranges (soil ratings from Wogi et al., Landon and Lindsay and Norvell; leaf optimum ranges in Table 8), units are mg/kg or % throughout, and the Discussion paragraphs were shortened and linked to the observed data.

**Comment 39:** *Conclusion: suggested rewording; use TN consistently; condense future-study sentences.*

**Response:** Done. The Conclusion was rewritten according to the new results and now also states the practical implications.

**Comment 40:** *Recommended related works for citation (GIS, remote sensing and geostatistics of soil quality and land degradation).*

**Response:** Thank you for these suggestions. These articles focus on spatial mapping and remote sensing of soil quality, salinity and land degradation. Our study is a plot-level leaf and soil analysis without spatial or remote sensing data, so we decided not to cite them, in line with the editor’s note that citing them is not required.

# Response to Reviewer #2

**Comment 1:** *The data analysis section required substantial revision; data analysis did not adequately capture the dataset. A rigorous recheck of statistical assumptions, model specifications, and computational outputs revealed critical discrepancies. Mean separation or standard error is required whether variabilities are statistically significant or not.*

**Response:** We thank the reviewer for this important comment and fully agree. We re-checked the raw data and redid the whole analysis. What we found and what we changed:

(1) Data and experimental unit. In the original manuscript, figures, tables and correlations were based on monthly means of the nine trees. We now use the tree-level values (each value is the mean of the three replicate analyses of the composite leaf sample of one tagged tree in one month; 36 observations for Hass at Mojo and 43–45 for each variety at MARC). The tree is the experimental unit. At Mojo, three Hass trees were sampled on each of three farms (9 trees per month; the farmers grow only Hass, which they prefer for export and local markets), and at MARC nine trees of each of the three varieties (27 trees per month). Mojo was not sampled in July because the fruit had reached harvest maturity. The full data are in S1 Dataset.

(2) Discrepancies corrected. The recheck showed (a) a transcription error: the March mean for Zn at Mojo was entered as 15.56 mg/kg, whereas the nine trees give 36.94 mg/kg; (b) values below the detection limit were treated as zero in some means and omitted in others (e.g., Fe at Mojo in May); they are now handled with one rule (half of the lowest quantified value, a common substitution) and counted in S1 Dataset, and a sensitivity check (ND set to a very low value or to the lowest quantified value) gave the same conclusions (S1 Table, sheet “ND_sensitivity”); (c) several values in the text did not match the data (e.g., Fuerte Mn and Fe); (d) the correlations had been calculated from four (Mojo) or five (MARC) monthly means.

(3) Model specification. Because the same tagged trees were sampled repeatedly, we used linear mixed models with tree as a random effect: variety × month for MARC, month (with farm and tree random; treating farm as fixed gave the same results, S1 Table) for Mojo, and site × month for Hass at the two sites over the four common months. Fixed effects were tested with type III F-tests and Kenward–Roger degrees of freedom (lme4/lmerTest). The F and P values are in S1 Table.

(4) Assumptions. Residual normality (Q–Q plots, Shapiro–Wilk) and homogeneity of variance (Levene’s test) were checked on the original and log scales. TN, Fe and Zn were right-skewed and analysed on the log scale; Ca, Cu and Mn on the original scale, and the same scale was used for each nutrient in all analyses (S1 Table, sheet “Residual_checks”). Because a few very high Fe and Zn values remained after log transformation, the Fe and Zn F-tests were repeated with the aligned rank transform (Wobbrock et al. 2011), which does not assume normal residuals. Every effect that was significant in the mixed models was also significant in this check (S1 Table, sheet “Robustness_ART”).

(5) Mean separation and standard errors. Means were separated with Tukey’s HSD test on estimated marginal means. All results are now given as mean ± SE with letters: Table 6 (Mojo), Table 7 (MARC, letters for months within each variety and for varieties within each month, plus the F-test P values), Table 8 (comparison with sufficiency ranges) and the soil data, where Tables 4 and 5 give the values of each Mojo farm and MARC sampling point together with the mean ± SE of each depth and the rating of each property. Letters are given wherever the corresponding F-test was significant (P < 0.05). Figs 2–4 show error bars (± SE) and Tukey letters or significance stars. Means and SE are reported whether or not the differences are significant, as the reviewer requested.

(6) Correlations. We replaced Pearson correlations on monthly means with repeated-measures correlation (Bakdash and Marusich 2017), which uses all tree × month observations and accounts for repeated sampling, and adjusted P values for multiple testing (Benjamini–Hochberg). Fig 5 shows the matrices and S2 Table gives r, df, N and raw and adjusted P values, with conventional pooled Pearson correlations for comparison.

(7) Effect on the conclusions. The corrected analysis changed some conclusions, and we revised the text accordingly: TN was below the sufficiency range everywhere, but the differences between varieties (P = 0.079), months and sites were not significant, and the “declining TN at Mojo” is not supported (P = 0.37); leaf Ca increased strongly from February to July (P < 0.001); Cu, Fe, Mn and Zn showed significant variety × month interactions; Hass at Mojo had higher Mn, Cu and Zn than at MARC; and the TN–Zn and Zn–Fe antagonisms reported earlier (based on correlations of four or five monthly means) are not supported by the tree-level data (r_rm = −0.08 to 0.11 and −0.33 to 0.14, not significant). Robust findings were positive correlations of Mn with Ca (all groups) and with Cu and Zn (Hass and Fuerte), and of Cu with Ca (Hass and Fuerte at MARC).

(8) Reproducibility. All R scripts (S1 Code) and data (S1 Dataset) are provided, so every table, statistic and figure can be reproduced.

# Response to the attached reviewer report

**General**

**Comment 1:** *The paper needs major revision to improve data analysis, result presentation, the coherence, and language improvement. The results need to be rewritten separately for Mojo and Melkassa sites without redundancy/repetition of results and discussion sections. Check and correct figures and Tables in the results.*

**Response:** Thank you. The data analysis was redone (see the response to Reviewer #2). The Results are now presented separately by site: soil at Mojo and at MARC, Mojo Hass leaves, then Melkassa (MARC) three varieties, followed by the comparison of Hass between sites, sufficiency ranges and relationships among nutrients. Repetition between Results and Discussion was removed. All figures and tables were checked against the raw data and regenerated; the 14 previous figures were replaced by 4 multi-panel figures (Figs 2–5), a map of the study area was added (Fig 1), and Tables 4–8 give means ± SE, with mean separation for the leaf data.

**Introduction**

**Comment 2:** *1st paragraph, insert citation.*

**Response:** Done; citations [1, 2] (Jalata 2021; Seid et al. 2023) now support the first sentence.

**Comment 3:** *Thorough language revision.*

**Response:** Done. The Introduction was shortened and rewritten in simple, clear language; it now also summarizes earlier Ethiopian avocado–soil studies to show the knowledge gap, and the objectives and hypotheses are stated explicitly.

**Materials and methods**

**Comment 4:** *A revision is required mainly for data analysis section.*

**Response:** Done; the section “Statistical analysis” was completely rewritten (see the response to Reviewer #2).

**Comment 5:** *Please indicate the year of data collection.*

**Response:** Done. Soil samples were collected in 2021 (December 2021 at Mojo) and leaf samples in February, March, May, June and July of the 2022 production season (sections “Soil sampling and analysis” and “Sampling of avocado leaves”).

**Comment 6:** *Data collection and analysis. The first sentence is very fragmented, please move to second paragraph. Please rework this section so that the soil sampling and agronomy data collection can be presented in a separate section. Is the soil analyzed before planting?*

**Response:** The fragmented sentences were rewritten, and leaf sampling (section “Sampling of avocado leaves”), leaf analysis (section “Leaf sample preparation and analysis”) and soil sampling (section “Soil sampling and analysis”) are now in separate sections. The soil section describes both sites: the three Mojo farms used for leaf sampling (two sampling points on farm 3) and three points in the MARC avocado orchard, all at 0–20 and 20–50 cm and analysed with the same methods.

The soil was not analysed before planting. The soil samples were collected in December 2021 from the established orchards, before the leaf sampling season; this is now stated in the section “Soil sampling and analysis”, and no pre-planting soil data are available.

**Results**

**Comment 7:** *Under the results section, the authors present Correlation analysis. But, it should not be title.*

**Response:** Agreed. The heading “Correlation analysis and antagonistic properties of macro and micronutrients” was replaced by “Relationships among leaf nutrients” in the Results, and the same heading is used in the Discussion.

**Comment 8:** *Figures. Reanalysis and generate all graphs.*

**Response:** Done. All graphs were regenerated in R from the tree-level data (Figs 2–5), with error bars (± SE) and mean separation letters or significance marks. The R code is provided as S1 Code. A map of the study area was also added (Fig 1).

**Comment 9:** *To ensure coherent flow of results, I suggest to present separately for Mojo and Melkassa sites.*

**Response:** Done, as described in Comment 1.

**Discussion**

**Comment 10:** *Refine titles and make coherent among sections.*

**Response:** Done. The Discussion now has four sections whose titles follow the Results: Soil properties of the study sites; Seasonal and varietal patterns in leaf nutrients; Relationships among leaf nutrients; Implications for nutrient management (with a limitations paragraph).

**Conclusion**

**Comment 11:** *It seems summary and need to add implications of the study.*

**Response:** Done. The Conclusion now has a short summary of the main findings and a second paragraph with the implications: N and Zn supply should be improved in both orchards, leaf Fe should be monitored on the alkaline soils of both sites, and leaf samples for diagnosis must be collected at a standard time of year; future work needs a full year at both sites, linked with yield and fruit quality.

**Comment 12:** *The study is based on orchard experimentation nutrient status of avocado varieties across locations and fruit tree age.*

**Response:** Thank you. We now state clearly that the two sites differ in tree age (planted in 2021 at Mojo and in 2001 at MARC), agroecology and soil, so the differences between sites cannot be attributed to one factor (Results and Discussion, and the limitations paragraph). We also explain that only Hass could be sampled at Mojo because the farmers grow only Hass, so the three varieties were compared at MARC (section “Sampling of avocado leaves”).

We hope the revised manuscript is now acceptable for publication in PLOS ONE.

Sincerely,

Zeyede Aregahegn (corresponding author), on behalf of all authors
