# Supplementary material for: Seasonal dynamics of leaf macro- and micronutrients in three commercial avocado varieties at Mojo and Melkassa, Ethiopia
Source: PLoS One. 2026 Nov 12;21(11):e0362564. doi: 10.1371/journal.pone.0362564 (PMC13759051; doi:10.1371/journal.pone.0362564)
Supplement: Peer review history

# Peer review: Decision Letter 0

**DOI:** 10.1371/journal.pone.0362564.r001

**Authors:** Raed Abduljabbar Haleem

17 Aug 2026

Dear Dr. Wondimgezahu,

Thank you for submitting your manuscript to PLOS ONE. After careful consideration, we feel that it has merit but does not fully meet PLOS ONE’s publication criteria as it currently stands. Therefore, we invite you to submit a revised version of the manuscript that addresses the points raised during the review process.

Please submit your revised manuscript by Oct 16 2026 11:59PM. If you will need more time than this to complete your revisions, please reply to this message or contact the journal office at plosone@plos.org. When you're ready to submit your revision, log on to https://www.editorialmanager.com/pone/ and select the 'Submissions Needing Revision' folder to locate your manuscript file.

- A letter that responds to each point raised by the academic editor and reviewer(s). You should upload this letter as a separate file labeled 'Response to Reviewers'.
- A marked-up copy of your manuscript that highlights changes made to the original version. You should upload this as a separate file labeled 'Revised Manuscript with Track Changes'.
- An unmarked version of your revised paper without tracked changes. You should upload this as a separate file labeled 'Manuscript'.

If you would like to make changes to your financial disclosure, please include your updated statement in your cover letter. Guidelines for resubmitting your figure files are available below the reviewer comments at the end of this letter.

If applicable, we recommend that you deposit your laboratory protocols in protocols.io to enhance the reproducibility of your results. Protocols.io assigns your protocol its own identifier (DOI) so that it can be cited independently in the future. For instructions see: https://journals.plos.org/plosone/s/submission-guidelines#loc-laboratory-protocols. Additionally, PLOS ONE offers an option for publishing peer-reviewed Lab Protocol articles, which describe protocols hosted on protocols.io. Read more information on sharing protocols at https://plos.org/protocols?utm_medium=editorial-email&utm_source=authorletters&utm_campaign=protocols.

As the corresponding author, your ORCID iD is verified in the submission system and will appear in the published article. PLOS supports the use of ORCID, and we encourage all coauthors to register for an ORCID iD and use it as well. Please encourage your coauthors to verify their ORCID iD within the submission system before final acceptance, as unverified ORCID iDs will not appear in the published article. *Only*  the individual author can complete the verification step; PLOS staff *cannot*  verify ORCID iDs on behalf of authors.

We look forward to receiving your revised manuscript.

Kind regards,

Raed Abduljabbar Haleem, Ph.D

Academic Editor

PLOS One

Journal Requirements:

When submitting your revision, we need you to address these additional requirements.

1. Please ensure that your manuscript meets PLOS ONE's style requirements, including those for file naming. The PLOS ONE style templates can be found at

https://journals.plos.org/plosone/s/file?id=wjVg/PLOSOne_formatting_sample_main_body.pdf and

https://journals.plos.org/plosone/s/file?id=ba62/PLOSOne_formatting_sample_title_authors_affiliations.pdf

2. Please note that PLOS One has specific guidelines on code sharing for submissions in which author-generated code underpins the findings in the manuscript. In these cases, we expect all author-generated code to be made available without restrictions upon publication of the work. Please review our guidelines at https://journals.plos.org/plosone/s/materials-and-software-sharing#loc-sharing-code and ensure that your code is shared in a way that follows best practice and facilitates reproducibility and reuse.

3. In your Methods section, please provide additional information regarding the permits you obtained for the work. Please ensure you have included the full name of the authority that approved the field site access and, if no permits were required, a brief statement explaining why.

4. Thank you for stating the following in the Acknowledgments Section of your manuscript:

"Ethiopian Institute of Agriculture Research is highly acknowledged for the provision of financial support for the

Integrated Fruits Research Project through the Integrated Research Program from 2021-2023. Integrated Fruit

Research Program is also highly acknowledged for facilitating activities and Melkassa Agricultural Research

Center for assisting in implementing this research activity."

We note that you have provided additional information within the Acknowledgements Section that is not currently declared in your Funding Statement. Please note that funding information should not appear in the Acknowledgments section or other areas of your manuscript. We will only publish funding information present in the Funding Statement section of the online submission form.

Please remove any funding-related text from the manuscript and let us know how you would like to update your Funding Statement. Currently, your Funding Statement reads as follows:

"The author(s) received no specific funding for this work."

Please include your amended statements within your cover letter; we will change the online submission form on your behalf.

5. Thank you for stating the following financial disclosure:

"The author(s) received no specific funding for this work."

At this time, please address the following queries:

a)        Please clarify the sources of funding (financial or material support) for your study. List the grants or organizations that supported your study, including funding received from your institution.

b)        State what role the funders took in the study. If the funders had no role in your study, please state: “The funders had no role in study design, data collection and analysis, decision to publish, or preparation of the manuscript.”

c)        If any authors received a salary from any of your funders, please state which authors and which funders.

d)        If you did not receive any funding for this study, please state: “The authors received no specific funding for this work.”

Please include your amended statements within your cover letter; we will change the online submission form on your behalf.

6. Please include your full ethics statement in the ‘Methods’ section of your manuscript file. In your statement, please include the full name of the IRB or ethics committee who approved or waived your study, as well as whether or not you obtained informed written or verbal consent. If consent was waived for your study, please include this information in your statement as well.

7. When completing the data availability statement of the submission form, you indicated that you will make your data available on acceptance. We strongly recommend all authors decide on a data sharing plan before acceptance, as the process can be lengthy and hold up publication timelines. Please note that, though access restrictions are acceptable now, your entire data will need to be made freely accessible if your manuscript is accepted for publication. This policy applies to all data except where public deposition would breach compliance with the protocol approved by your research ethics board. If you are unable to adhere to our open data policy, please kindly revise your statement to explain your reasoning and we will seek the editor's input on an exemption. Please be assured that, once you have provided your new statement, the assessment of your exemption will not hold up the peer review process.

8. Please amend the manuscript submission data (via Edit Submission) to include authors Edossa Etissa, Israel Bekele, Dejene Abera.

9. If the reviewer comments include a recommendation to cite specific previously published works, please review and evaluate these publications to determine whether they are relevant and should be cited. There is no requirement to cite these works unless the editor has indicated otherwise.

[Note: HTML markup is below. Please do not edit.]

Reviewers' comments:

Reviewer's Responses to Questions

**Comments to the Author**

1. Is the manuscript technically sound, and do the data support the conclusions?

Reviewer #1: Yes

Reviewer #2: Partly

**********

2. Has the statistical analysis been performed appropriately and rigorously?

Reviewer #1: Yes

Reviewer #2: No

**********

3. Have the authors made all data underlying the findings in their manuscript fully available?

The PLOS Data policy

Reviewer #1: Yes

Reviewer #2: Yes

**********

4. Is the manuscript presented in an intelligible fashion and written in standard English?

Reviewer #1: Yes

Reviewer #2: Yes

**********

Reviewer #1: Title

[Word choice]: “Levels and seasonal patterns…” → Consider “Seasonal dynamics of macro- and micronutrients…” for scientific precision.

[Clarity]: “soil nutrient status of avocado farms” → Revise to “soil nutrient profiles in avocado orchards” for conciseness.

Abstract

[Sentence clarity]: “Avocado fruit, rich in essential nutrients and highly productive, is a recommended crop…” → Remove “highly productive” (redundant).

[Word choice]: “0 ppm” → Replace with “below detection limit” for scientific accuracy.

[Sentence structure]: “Pearson correlation analysis revealed synergistic relationships…” → Break into two sentences for readability: one for synergistic, one for antagonistic.

: Final sentence (“Future studies correlating…”) is speculative; move to “Conclusion” section instead of Abstract.

Keywords

[Typo]: “Moj” → Correct to “Mojo.”

[Consistency]: Use “soil fertility; nutrient dynamics; avocado leaf analysis” — avoid mixing site names with general terms.

Introduction

[Sentence clarity]: “Avocado shows great potential across various agroecological zones…” → Replace “shows great potential” with “is well adapted.”

[Redundancy]: Hass, Fuerte, and Ettinger described twice (lines 2–3 and 6–7). Merge into one concise paragraph.

[Word choice]: “particularly prolific” → Replace with “highly productive” for scientific tone.

[Sentence structure]: “The concentration of macronutrients… varies at different development stages…” → Recast as “Macronutrient and micronutrient concentrations vary across phenological stages (flowering, fruiting, maturity, post-harvest).”

: Photosynthesis explanation is basic; condense to one sentence linking leaf physiology to nutrient demand.

[Word choice]: “offers several key advantages: including…” → Remove “including” after colon; redundant.

[Sentence clarity]: “Analyzing the seasonal nutrient levels… is essential for determining the appropriate timing…” → Replace “appropriate timing” with “optimal fertilization schedule.”

: Last two paragraphs overlap (nutrient balance, deficiencies, seasonal variation). Merge into one streamlined paragraph.

Overall Notes

[Consistency]: Use either “nutrient dynamics” or “nutrient status” consistently.

[Scientific tone]: Avoid promotional phrases (“stimulate commercial and economic activities”) in Introduction; focus on agronomic relevance.

[Precision]: Replace vague terms (“good soil,” “healthy tree”) with measurable descriptors (e.g., “soil with adequate organic matter and balanced pH”).

Materials and Methods section

2.1 Sampling site

[Word choice]: “specifically for the Hass variety” → Replace with “restricted to Hass variety.”

[Sentence clarity]: “Melkassa Agricultural Research Center (MARC) avocado experimental site is located at the Center station” → Redundant; simplify to “MARC avocado experimental site is located at the Center.”

[Consistency]: Use “coordinates” instead of “geographic coordinates” (already implied).

: Merge location details into a concise table or sentence; current form is verbose.

2.2 Soil sampling and analysis

[Typo]: “cupper (Cu)” → Correct to “copper (Cu).”

[Sentence clarity]: “Samples were taken in two depths 0-20 cm and 20-50 cm” → Revise to “Samples were collected at two depths: 0–20 cm and 20–50 cm.”

[Word choice]: “following standard procedure” → Specify “following standard protocols [11, 28, 33].”

2.3 Sampling of avocado leaves

[Grammar]: “None shaded branches” → Correct to “Non-shaded branches.”

[Word choice]: “choosen” → Correct to “chosen.”

[Sentence clarity]: “due to it’s the common commercial avocado variety” → Revise to “as it is the predominant commercial variety.”

: Sampling description is repetitive (tree selection, tagging, random sampling). Condense into one structured paragraph.

2.4 Leaf sample preparation and analysis

[Word choice]: “grinded” → Correct to “ground.”

[Sentence clarity]: “transferred to acid-washed porcelain crucibles and transferred to a muffle furnace” → Avoid repetition; revise to “placed in acid-washed porcelain crucibles and ashed in a muffle furnace.”

[Precision]: “500 0C” → Correct to “500 °C.”

2.5 Apparatus and equipment

[Sentence clarity]: “Fully automated Flame Atomic Absorption Spectrophotometer (FAAS) (Agilent 200 Series AA, USA) with fast sequential operation for multi-element analysis” → Simplify to “FAAS (Agilent 200 Series AA, USA) was used for multi-element analysis.”

[Word choice]: “Digital furnace” → Replace with “Programmable furnace.”

2.6 Reagents and standards

[Sentence clarity]: “to prepare a serious of standards” → Correct to “to prepare a series of standards.”

[Consistency]: Use “analytical-grade reagents” instead of “analytical grade.”

2.7 Calibration

[Sentence clarity]: “five working standard solutions were prepared for the calibration curve of each element” → Revise to “five working standards were prepared to construct calibration curves for each element.”

[Word choice]: “micronutrient (Fe, Cu, Mn, Zn) standards” → Replace with “Fe, Cu, Mn, and Zn standards.”

2.8 Quality assurance and quality control

[Sentence clarity]: “Table 1: Standard conditions…” → Add “shown in Table 1” within text instead of standalone.

[Precision]: “Optimum working range µg/mL” → Clarify units consistently (mg/L or µg/mL).

2.9 Recovery analysis

[Sentence clarity]: “determination of recovery after spike of the leaf and soil sample extract” → Revise to “recovery determined by spiking leaf and soil extracts.”

[Word choice]: “spike” → Replace with “spiking.”

2.10 LOD and LOQ

[Formatting]: Equations (2) and (3) misaligned; ensure proper LaTeX formatting.

[Sentence clarity]: “Where, LOD is limit of detection…” → Remove comma after “Where.”

2.11 Data analysis

[Sentence clarity]: “to analyze the data, to calculate Pearson correlation coefficient, and to draw graphs” → Simplify to “for statistical analysis, Pearson correlation, and graphing.”

[Word choice]: Replace “subjected to R software” with “analyzed using R software.”

Results section

3.1 Soil nutrient status

[Word choice]: “reviled” → Correct to “revealed.”

[Sentence clarity]: “which does not impact avocado production” → Revise to “which is within acceptable range for avocado growth.”

[Consistency]: “cupper (Cu)” → Correct to “copper (Cu).”

: Soil texture data in Table 4 is overly detailed; condense into one sentence summarizing dominant classes.

3.2 Avocado leaf nitrogen

[Word choice]: “occured” → Correct to “occurred.”

[Sentence clarity]: “suggesting peak nitrogen accumulation during this peored” → Correct “peored” to “period.”

[Consistency]: Use “Total nitrogen (TN)” consistently instead of alternating “nitrogen level” and “TN.”

: Figures 1–6 descriptions are repetitive; merge into one comparative narrative.

3.3 Micronutrients mean levels

[Sentence clarity]: “Elevated levels of copper (Cu) and iron (Fe) were observed in July compared to the other sampling months” → Simplify to “Cu and Fe peaked in July.”

[Word choice]: “minimal” → Replace with “lowest” for precision.

: Combine narrative with Table 5 comparison instead of repeating ranges in text.

3.4 Micronutrients by variety and location

[Sentence clarity]: “Zn levels exhibited variability across the sampling months, with notable increases in May and June” → Condense to “Zn increased in May–June.”

[Word choice]: “initially low or absent” → Replace with “below detection limit.”

: Figures 8–12 descriptions overlap; integrate into one comparative paragraph.

3.5 Calcium concentration

[Sentence clarity]: “Ca levels peaked in July, followed by June and May” → Revise to “Ca peaked in July, with secondary peaks in June and May.”

[Word choice]: “lowest overall concentration of Ca” → Replace with “lowest mean Ca concentration.”

[Consistency]: Use “Ca concentration (%)” consistently; avoid switching to ppm.

3.6 Correlation analysis

[Sentence clarity]: “authors [10]” → Replace with “previous studies [10].”

[Word choice]: “minimal interaction” → Replace with “weak correlation.”

: Summarize correlation matrices in text; avoid repeating table values verbatim.

[Consistency]: Ensure uniform reporting of correlation strength (e.g., “strong positive,” “weak negative”).

Overall Notes

[Precision]: Replace vague terms (“adequate,” “normal range”) with quantitative thresholds or references.

[Consistency]: Standardize units (ppm vs %) across tables and figures.

[Clarity]: Figures are over-described; streamline captions and integrate key findings into text.

Discussion section

4.1 Soil Nutrient Status at Mojo Site

[Sentence clarity]: “The pH of the soil at the Mojo site which is slightly alkaline allows for the avocado cultivation” → Revise to “The slightly alkaline soil pH at Mojo supports avocado cultivation.”

[Word choice]: “rated as low to moderate” → Replace with “classified as low to moderate.”

[Redundancy]: Copper functions described twice (enzyme activity, reproductive growth); condense into one concise statement.

[Consistency]: Use “organic carbon (OC)” consistently instead of alternating “OM” and “TOC.”

: EC and CEC discussion is overly detailed; summarize in one sentence linking values to salinity risk.

[Word choice]: “lovel of Mn” → Correct to “level of Mn.”

Avocado Leaf Sample Analysis

[Sentence clarity]: “The fluctuation of nitrogen levels… indicates that nitrogen availability may be influenced…” → Simplify to “Nitrogen fluctuations suggest influence of seasonal and management factors.”

[Word choice]: “less efficient in nitrogen uptake” → Replace with “lower nitrogen uptake efficiency.”

: Redistribution explanation is lengthy; condense to one sentence linking mobility to observed varietal differences.

4.2 Correlation Analysis

[Sentence clarity]: “This antagonism could have critical consequences for plant health…” → Revise to “This antagonism may impair zinc-dependent processes.”

[Word choice]: “plays a central role” → Replace with “is pivotal.”

[Consistency]: Use “positive correlation” and “negative correlation” consistently instead of mixing “synergistic” and “antagonistic.”

: Merge Cu–Ca discussion with Mn–Ca to avoid repetition.

4.3 Implications for Nutrient Management

[Sentence clarity]: “need for supplementation” → Revise to “require supplementation.”

[Word choice]: “one-size-fits-all approach” → Replace with “uniform fertilization strategy.”

[Consistency]: Use “leaf nutrient analysis” consistently instead of alternating “leaf analysis” and “nutrient analysis.”

: Final sentences on organic amendments overlap; condense into one statement emphasizing soil health improvement.

Overall Notes

[Precision]: Replace vague terms (“adequate,” “optimal”) with quantitative thresholds or literature references.

[Consistency]: Standardize reporting of nutrient ranges (use ppm or % consistently).

[Clarity]: Several paragraphs are overly descriptive; streamline by integrating physiological explanations with observed data.

5. Conclusion

[Sentence clarity]: “The soil analysis of Mojo avocado farms revealed very low iron (Fe) and zinc (Zn) levels…” → Revise to “Soil analysis at Mojo revealed critically low Fe and Zn.”

[Word choice]: “Leaf analysis across Hass, Fuerte, and Ettinger varieties showed TN levels in the order…” → Replace with “Leaf analysis indicated TN ranking: Hass > Fuerte > Ettinger.”

[Consistency]: Use “TN” consistently instead of alternating “total nitrogen” and “TN.”

[Sentence clarity]: “Pearson correlation analysis identified key nutrient interactions…” → Condense to “Correlation analysis revealed synergistic (Mn–Cu–Ca) and antagonistic (TN–Zn, Zn–Fe) interactions.”

[Word choice]: “highlight the need for improved nutrient management strategies” → Replace with “underscore the requirement for targeted nutrient management.”

: Final sentences on future studies overlap; condense into one statement linking nutrient monitoring with agronomic outcomes.

[Sentence clarity]: “evaluate nutrient removal through harvest to determine precise annual nutrient requirements” → Revise to “assess nutrient removal via harvest to define annual requirements.”

Recommended Related Works for Citation

Here are selected publications that align with your manuscript’s focus on soil nutrient status, leaf nutrient analysis, and management strategies:

Soil Fertility & Nutrient Assessment

“Estimating soil fertility status in physically degraded land using GIS and remote sensing techniques” (Egyptian Journal of Remote Sensing and Space Science, 2016) – Relevant for linking soil nutrient mapping with fertility evaluation.

“Deciphering soil spatial variability through geostatistics and interpolation techniques” (Sustainability, 2020) – Useful for discussing variability in soil nutrients across sites.

Soil Quality & Land Degradation

“Assessment of land degradation using comprehensive geostatistical approach and remote sensing data in GIS-model builder” (2018) – Supports your soil nutrient imbalance discussion.

“Comparison of two soil quality indices using GIS-based methods” (2018) – Can be cited when discussing soil fertility indicators and nutrient management.

Remote Sensing & Nutrient Mapping

“Integrating active and passive remote sensing data for mapping soil salinity using machine learning” (Remote Sensing, 2023) – Relevant for methodological parallels in soil nutrient monitoring.

“GIS model-builder based on comprehensive geostatistical approach to assess soil quality” (Remote Sensing Applications: Society and Environment, 2019) – Strengthens your methodological framework.

Sustainable Land Management

“An overview of land degradation, desertification and sustainable land management using GIS and remote sensing applications” (Rendiconti Lincei, 2023) – Provides a broader sustainability context for your nutrient management recommendations.

“Land degradation vulnerability mapping in a newly-reclaimed desert oasis in a hyper-arid agro-ecosystem using AHP and geospatial techniques” (Agronomy, 2021) – Relevant for site-specific nutrient management strategies.

Reviewer #2: The data analysis section required substantial revision; data analysis did not adequately capture the dataset . A rigorous recheck of statistical assumptions, model specifications, and computational outputs revealed critical discrepancies. Mean separation or standard error is required whether variabilities are statistically significant or not.

**********

what does this mean?). If published, this will include your full peer review and any attached files.

If you choose “no”, your identity will remain anonymous but your review may still be made public.

**Do you want your identity to be public for this peer review?**  For information about this choice, including consent withdrawal, please see our Privacy Policy

Reviewer #1: **Yes:**  Mohamed A. E. AbdelRahman

Reviewer #2: No

**********

[NOTE: If reviewer comments were submitted as an attachment file, they will be attached to this email and accessible via the submission site. Please log into your account, locate the manuscript record, and check for the action link "View Attachments". If this link does not appear, there are no attachment files.]

To ensure your figures meet our technical requirements, please review our figure guidelines: https://journals.plos.org/plosone/s/figures

You may also use PLOS’s free figure tool, NAAS, to help you prepare publication quality figures: https://journals.plos.org/plosone/s/figures#loc-tools-for-figure-preparation.

NAAS will assess whether your figures meet our technical requirements by comparing each figure against our figure specifications.

Attachment Submitted filename: PONE-D-26-25421.pdf

Attachment Submitted filename: Review.docx

# Author comment: Author response to Decision Letter 1

**DOI:** 10.1371/journal.pone.0362564.r002

13 Oct 2026

Dear Editor,

Thank you and the reviewers for the careful and helpful comments. We have addressed every comment from the editor, Reviewer #1, Reviewer #2 and the attached reviewer report. Our point-by-point responses are provided in the uploaded file "Response to Reviewers", and all changes are shown in the file "Revised Manuscript with Track Changes".

In summary, following Reviewer #2, all leaf data were re-analysed from the tree-level data with linear mixed models, Tukey's test (mean ± SE for every group) and repeated-measures correlation; the results are presented separately for Mojo and Melkassa; soil data for both sites were added and rated against standard classes; and the manuscript was revised for language and PLOS ONE style. The R code and full data are provided as S1 Code and S1 Dataset.

Sincerely,

Zeyede Aregahegn, on behalf of all authors

Attachment Submitted filename: Response_to_Reviewers.docx

# Editor report: Decision Letter 1

**DOI:** 10.1371/journal.pone.0362564.r003

**Authors:** Raed Abduljabbar Haleem

28 Oct 2026

Seasonal dynamics of leaf macro- and micronutrients in three commercial avocado varieties at Mojo and Melkassa, Ethiopia

PONE-D-26-25421R1

Dear Dr. Wondimgezahu,

We’re pleased to inform you that your manuscript has been judged scientifically suitable for publication and will be formally accepted for publication once it meets all outstanding technical requirements.

Within one week, you’ll receive an e-mail detailing the required amendments. When these have been addressed, you’ll receive a formal acceptance letter and your manuscript will be scheduled for publication.

An invoice will be generated when your article is formally accepted. Please note, if your institution has a publishing partnership with PLOS and your article meets the relevant criteria, all or part of your publication costs will be covered. Please make sure your user information is up-to-date by logging into Editorial Manager at Editorial Manager® and clicking the ‘Update My Information' link at the top of the page. For questions related to billing, please contact billing support.

If your institution or institutions have a press office, please notify them about your upcoming paper to help maximize its impact. If they’ll be preparing press materials, please inform our press team as soon as possible -- no later than 48 hours after receiving the formal acceptance. Your manuscript will remain under strict press embargo until 2 pm Eastern Time on the date of publication. For more information, please contact onepress@plos.org.

Kind regards,

Raed Abduljabbar Haleem, Ph.D

Academic Editor

PLOS One

# Editor report: Acceptance letter

**DOI:** 10.1371/journal.pone.0362564.r004

**Authors:** Raed Abduljabbar Haleem

PONE-D-26-25421R1

PLOS One

Dear Dr. Aregahegn,

I'm pleased to inform you that your manuscript has been deemed suitable for publication in PLOS One. Congratulations! Your manuscript is now being handed over to our production team.

At this stage, our production department will prepare your paper for publication. This includes ensuring the following:

* All references, tables, and figures are properly cited

* All relevant supporting information is included in the manuscript submission,

* There are no issues that prevent the paper from being properly typeset

You will receive further instructions from the production team, including instructions on how to review your proof when it is ready. Please keep in mind that we are working through a large volume of accepted articles, so please give us a few days to review your paper and let you know the next and final steps.

Lastly, if your institution or institutions have a press office, please let them know about your upcoming paper now to help maximize its impact. If they'll be preparing press materials, please inform our press team within the next 48 hours. Your manuscript will remain under strict press embargo until 2 pm Eastern Time on the date of publication. For more information, please contact onepress@plos.org.

You will receive an invoice from PLOS for your publication fee after your manuscript has reached the completed accept phase. If you receive an email requesting payment before acceptance or for any other service, this may be a phishing scheme. Learn how to identify phishing emails and protect your accounts at https://explore.plos.org/phishing.

If we can help with anything else, please email us at customercare@plos.org.

Thank you for submitting your work to PLOS One and supporting open access.

Kind regards,

PLOS One Editorial Office Staff

on behalf of

Dr. Raed Abduljabbar Haleem

Academic Editor

PLOS One
